# Supplementary material for: High-throughput target trial emulation for Alzheimer’s disease drug repurposing with real-world data
Source: Nat Commun. 2023 Dec 11;14:8180. doi: 10.1038/s41467-023-43929-1 (PMC10713627; doi:10.1038/s41467-023-43929-1)
Supplement: Supplementary file 1 — Supplementary Information [file 41467_2023_43929_MOESM1_ESM.pdf]

Supplementary Materials for

High-Throughput Target Trial Emulation for Alzheimer's Disease Drug Repurposing with Real-World Data

Chengxi Zang[1,2], Hao Zhang[1], Jie Xu[3], Hansi Zhang[3], Sajjad Fouladvand[4], Shreyas Havaldar[5], Feixiong Cheng[6,7,8], Kun Chen[9], Yong Chen[10], Benjamin S. Glicksberg[5], Jin Chen[4], Jiang Bian[3], and Fei Wang[1,2,*]

[1]Department of Population Health Sciences, Weill Cornell Medicine, New York, NY, USA

[2] Institute of Artificial Intelligence for Digital Health, Weill Cornell Medicine, New York, NY, USA

[3]Department of Health Outcomes & Biomedical Informatics, University of Florida, Gainesville, FL, USA

[4]Institude for Biomedical Informatics (IBI) and Department of Computer Science, University of Kentucky, Lexington, KY, USA

[5]Hasso Plattner Institute for Digital Health at Mount Sinai, Icahn School of Medicine at Mount Sinai, New York, New

York, USA

[6]Genomic Medicine Institute, Lerner Research Institute, Cleveland Clinic, Cleveland, OH, USA

[7]Department of Molecular Medicine, Cleveland Clinic Lerner College of Medicine, Case Western Reserve University, Cleveland, OH, USA

[8]Case Comprehensive Cancer Center, Case Western Reserve University School of Medicine, Cleveland, OH, USA

[9]Department of Statistics, University of Connecticut, Storrs, CT, USA

[10]Department of Biostatistics, Epidemiology and Informatics (DBEI), the Perelman School of Medicine, University of Pennsylvania, Philadelphia, PA, USA

[*]corresponding author: Fei Wang (few2001@med.cornell.edu)

Table of Content

- **Supplementary Table S1.** Population characteristics of the two real-world healthcare databases for high-throughput trial emulation.
- **Supplementary Table S2.** Balance performance of different ML-PS models, including LR, GBM, MLP, and LSTM, under the best model selection practice, OneFlorida, 2012-2020.
- **Supplementary Table S3.** Selected ICD-9/10 diagnosis codes for Mild Cognitive Impairment (MCI) and Alzheimer's Disease (AD).
- **Supplementary Table S4.** Baseline comorbidity ICD codes (at the end due to size)
- **Supplementary Table S5.** Trial characteristics and estimated treatment effects of drug candidates selected from the OneFlorida, 2012-2020.
- **Supplementary Table S6.** Trial characteristics and estimated treatment effects of drug candidates from the MarketScan, 2009-2020.
- **Supplementary Table S7**. Simulation study results summary.
- **Supplementary Fig. S1.** Balance performance of Gradient Boosting Machine-based Propensity Score models (GBM-PS) selected by different model selection strategies, OneFlorida database, 2012-2020.
- **Supplementary Fig. S2.** Balance performance of Multi-Layer Lerceptron-based Propensity Score models (MLP-PS) selected by different model selection strategies, OneFlorida database, 2012-2020.
- **Supplementary Fig. S3.** Balance performance of Long Short Term Memory neural networks-based Propensity Score models (LSTM-PS) selected by different model selection strategies, OneFlorida database, 2012-2020.
- **Supplementary Fig. S4.** Balance performance of the regularized Logistic Regression-based Propensity Score (LR-PS) models selected by different model selection strategies under the Nested Cross-Validation framework, OneFlorida, 2012-2020.
- **Supplementary Fig. S5.** Balance performance of the regularized Logistic Regression-based Propensity Score models (LR-PS) selected by different model selection strategies, MarketScan database, 2009-2020.
- **Supplementary Fig. S6.** Balance performance of Long Short Term Memory neural networks-based Propensity Score models (LSTM-PS) selected by different model selection strategies, MarketScan database, 2009-2020.
- **Supplementary Fig. S7.** Generated drug repurposing hypotheses for AD with adjusted hazard ratios within the two-year follow-up period.
- **Supplementary Fig. S8.** Data generation diagram and generated time-to-event samples in the simulation study.
- **Supplementary Fig. S9.** Balance performance and outcome estimates after IPTW in the simulation study, Part I.
- **Supplementary Fig. 10.** Balance performance and outcome estimates after IPTW in the simulation study, Part II.
- **Supplementary Method**. Causal Discovery of DAGs for emulated trials.

Supplementary Table S1. Population characteristics of the two real-world healthcare databases for high-throughput trial emulation.

OneFlorida, January 2012 to April 2020, 14,883,388 patients				
	**MCI**	**AD**	**MCI \ AD**	**P-value[a]**
No. of patients	73,927 (100%)	10,530 (14.24%)	63,397 (85.76%)	–
MCI age, median (IQR) [c]	66 (50, 77)	78 (71, 85)	64 (46, 75)	0.000
Sex-female	40,654 (54.99%)	6,681 (63.45%)	33,973 (53.59%)	0.000[b]
Sex-male	33,273 (45.01%)	3,849 (36.55%)	29,424 (46.41%)	–
Antidiabetic medication	19,307 (26.12%)	2,563 (24.34%)	16,744 (26.41%)	0.000
Antihypertensives medication	10,909 (14.76%)	1,208 (11.47%)	9,701 (15.30%)	0.000
Alcohol Use Disorders	7,728 (10.45%)	709 (6.73%)	7,019 (11.07%)	0.000
Anxiety Disorders	38,602 (52.22%)	6,009 (57.07%)	32,593 (51.41%)	0.000
Depression	41,763 (56.49%)	6,979 (66.28%)	34,784 (54.87%)	0.000
Diabetes	29,761 (40.26%)	5,640 (53.56%)	24,121 (38.05%)	0.000
Heart Failure	17,842 (24.13%)	4,019 (38.17%)	13,823 (21.80%)	0.000
Hyperlipidemia	45,902 (62.09%)	8,185 (77.73%)	37,717 (59.49%)	0.000
Hypertension	52,834 (71.47%)	9,474 (89.97%)	43,360 (68.39%)	0.000
Ischemic Heart Disease	27,793 (37.60%)	6,001 (56.99%)	21,792 (34.37%)	0.000
Obesity	22,767 (30.80%)	2,739 (26.01%)	20,028 (31.59%)	0.000
Stroke/Transient Ischemic Attack	20,487 (27.71%)	4,143 (39.34%)	16,344 (25.78%)	0.000
Tobacco Use	16,344 (22.11%)	1,708 (16.22%)	14,636 (23.09%)	0.000
Traumatic Brain Injury	5,980 (8.09%)	720 (6.84%)	5,260 (8.30%)	0.000
Sleep disorders	29,691 (40.16%)	4,323 (41.05%)	25,368 (40.01%)	0.045
Periodontitis	1,024 (1.39%)	97 (0.92%)	927 (1.46%)	0.000
Menopause	289 (0.39%)	13 (0.12%)	276 (0.44%)	0.000
**MarketScan, January 2009 to June 2020, 164,148,434 patients**				
	**MCI**	**AD**	**MCI \ AD**	**P-value**
No. of patients	424,961 (100%)	67,973 (16.00%)	356,988 (84.00%)	–
MCI age, median (IQR)[c]	64 (49, 79)	80 (73, 86)	61 (45, 76)	0.000
Sex-female	230,732 (54.29%)	39,424 (58.00%)	191,308 (53.59%)	0.000[b]
Sex-male	194,229 (45.71%)	28,549 (42.00%)	165,680 (46.41%)	–
Antidiabetic medication	65,093 (15.32%)	11,840 (17.42%)	53,253 (14.92%)	0.000
Antihypertensives medication	161,904 (38.10%)	33,403 (49.14%)	128,501 (36.00%)	0.000
Alcohol Use Disorders	27,112 (6.38%)	3,430 (5.05%)	23,682 (6.63%)	0.000
Anxiety Disorders	180,771 (42.54%)	29,012 (42.68%)	151,759 (42.51%)	0.412
Depression	210,388 (49.51%)	37,986 (55.88%)	172,402 (48.29%)	0.000
Diabetes	124,903 (29.39%)	25,232 (37.12%)	99,671 (27.92%)	0.000
Heart Failure	81,582 (19.20%)	21,745 (31.99%)	59,837 (16.76%)	0.000
Hyperlipidemia	266,840 (62.79%)	53,061 (78.06%)	213,779 (59.88%)	0.000
Hypertension	282,484 (66.47%)	59,938 (88.18%)	222,546 (62.34%)	0.000
Ischemic Heart Disease	135,972 (32.00%)	33,501 (49.29%)	102,471 (28.70%)	0.000
Obesity	84,530 (19.89%)	9,638 (14.18%)	74,892 (20.98%)	0.000
Stroke/Transient Ischemic Attack	122,531 (28.83%)	27,784 (40.88%)	94,747 (26.54%)	0.000
Tobacco Use	46,010 (10.83%)	5,145 (7.57%)	40,865 (11.45%)	0.000
Traumatic Brain Injury	42,635 (10.03%)	6,255 (9.20%)	36,380 (10.19%)	0.000
Sleep disorders	157,272 (37.01%)	23,566 (34.67%)	133,706 (37.45%)	0.000
Periodontitis	2,202 (0.52%)	361 (0.53%)	1,841 (0.52%)	0.629
Menopause	4,184 (0.98%)	328 (0.48%)	3,856 (1.08%)	0.000

[a] Two-sided T-test for the null hypothesis that two independent samples (population with AD diagnosis v.s. population without any AD diagnosis) have identical average values, except for sex [b] Chi-square test of independence of the observed male and female frequencies. [c] MCI age is the sample median with inter-quartile range (IQR, 25th to 75th percentile).

Supplementary Table S2. Balance performance [a] of different ML-PS models [b], including LR, GBM, MLP, and LSTM, under the best model selection practice, OneFlorida, 2012-2020. The best balance performance was highlighted in bold.

Drug	No. of Treated	No. of Control[c]	ML-PS Models	No. of unbalanced feat. before re-weighting[d]	No. of unbalanced feat. after re-weighting	Pvalue (LR vs. others)[e]	No. of balanced trials after re weigting (%)	No. of unbalanced feat. in balanced trials after re weighting	Pvalue (LR vs. others)[e]
**aspirin**	1532	4576.4	**LR**	**50.9**	**0.3**	—	**100**	**0.3**	—
			GBM	50.9	3.4	1.1E-28	87	2.9	5.3E-30
			MLP	50.9	8.1	4.6E-23	35	4	1.1E-25
			LSTM	48.6	5.4	1.7E-08	78	2.6	3.6E-26
**atorvastatin**	1674	3181.7	**LR**	**67.6**	**0.8**	—	**99**	**0.8**	—
			GBM	67.6	2.8	8.5E-15	88	2.3	1.5E-13
			MLP	67.6	7	1.2E-18	45	3.5	7.9E-21
			LSTM	30.9	5.3	7.6E-08	68	1.8	6.9E-06
**pantoprazole**	1100	2508	**LR**	**114.6**	**0.7**	—	**99**	**0.6**	—
			GBM	114.6	6.5	1.1E-34	40	3.4	4.3E-16
			MLP	114.6	15.8	2.7E-38	0	—	—
			LSTM	38.7	2.5	7.6E-10	93	2	1.7E-11
**acetaminophe n**	1837	3376.3	**LR**	**50.6**	**1.6**	—	**93**	**1.2**	—
			GBM	50.6	8.8	8.7E-17	50	2.1	2.4E-04
			MLP	50.6	11.2	2.1E-20	25	4	1.4E-12
			LSTM	45.9	2.7	1.6E-03	84	1.7	3.5E-02
**famotidine**	842	2461.7	**LR**	**96.5**	**1.5**	—	**93**	**1**	—
			GBM	96.5	8.7	7.3E-30	22	4.5	7.5E-24
			MLP	96.5	12.1	7.3E-25	13	4	2.2E-12
			LSTM	27.5	10.7	2.6E-20	24	4	2.7E-14
**gabapentin**	1237	2426.7	**LR**	**107**	**1.8**	—	**91**	**1.2**	—
			GBM	107	12.5	2.3E-25	32	3.9	3.0E-19
			MLP	107	11.3	1.1E-25	21	3.1	7.3E-06
			LSTM	50.5	7.1	8.4E-17	41	3.5	3.1E-13
**trazodone**	1126	3370.9	**LR**	**92.4**	**2.6**	—	**89**	**1.9**	—
			GBM	92.4	7.8	2.0E-21	37	3.9	3.1E-12
			MLP	92.4	14.3	1.4E-25	0	—	—
			LSTM	48.2	6.6	3.4E-08	55	2.7	1.9E-03
**amlodipine**	930	2787.1	**LR**	**52.8**	**3.3**	—	**81**	**2.1**	—
			GBM	52.8	12.2	6.9E-41	0	—	—
			MLP	52.8	15.9	1.7E-11	0	—	—
			LSTM	39.1	10	9.6E-16	28	3.9	5.9E-09
**mirtazapine**	810	2428.1	**LR**	**73.5**	**4.3**	—	**76**	**2.1**	—
			GBM	73.5	14	5.0E-15	21	3.7	2.9E-07
			MLP	73.5	17.6	5.4E-24	0	—	—
			LSTM	48.2	11	1.9E-08	40	2.6	6.5E-02

**omeprazole**	917	2120	**LR**	**90.7**	**4.2**	—	**76**	**2.5**	—
			GBM	90.7	11.7	3.4E-26	0	—	—
			MLP	90.7	12.3	5.4E-15	21	3.7	2.0E-04
			LSTM	80.5	13.4	3.9E-16	0	—	—
**lisinopril**	950	2240.5	**LR**	**54.3**	**4**	—	**72**	**1.6**	—
			GBM	54.3	13.1	1.8E-25	0	—	—
			MLP	54.3	11.1	8.6E-14	23	4.3	1.2E-13
			LSTM	38.2	10.9	1.7E-08	29	3.8	6.8E-11
**sertraline**	709	2127	**LR**	**57.5**	**4.8**	—	**71**	**2.5**	—
			GBM	57.5	14.8	1.3E-27	0	—	—
			MLP	57.5	12.7	9.1E-11	15	3.9	7.2E-04
			LSTM	39.6	11.2	1.5E-09	33	3.4	2.9E-03
**albuterol**	1045	2206.9	**LR**	**85.8**	**5.2**	—	**66**	**2.8**	—
			GBM	85.8	19.1	3.2E-48	0	—	—
			MLP	85.8	21.6	1.9E-36	0	—	—
			LSTM	57.7	8.4	1.1E-07	26	3.9	1.3E-04
**metoprolol**	892	1682.5	**LR**	**60.5**	**5.6**	—	**60**	**3**	—
			GBM	60.5	20.1	2.6E-21	0	—	—
			MLP	60.5	16	5.2E-34	0	—	—
			LSTM	37.7	12.3	2.1E-15	0	—	—
**fluticasone**	903	2704.5	**LR**	**88.2**	**5.7**	—	**56**	**2.9**	—
			GBM	88.3	14.4	1.2E-24	0	—	—
			MLP	88.2	17.7	1.9E-25	0	—	—
			LSTM	64.2	21.9	3.3E-18	0	—	—
**escitalopram**	767	2300.4	**LR**	**70**	**9.4**	—	**52**	**1.8**	—
			GBM	70	18.4	4.5E-08	10	3.7	1.6E-04
			MLP	70	17.7	2.3E-06	15	4.3	5.1E-11
			LSTM	54.5	16.2	4.8E-05	26	3.4	9.5E-06
**losartan**	801	2296.8	**LR**	**81.1**	**10.4**	—	**49**	**2.4**	—
			GBM	81.1	20.1	4.9E-11	0	—	—
			MLP	81.1	22.1	7.7E-14	0	—	—
			LSTM	56	15.9	2.4E-03	25	2.8	1.9E-01
**meloxicam**	675	2024.1	**LR**	**91.7**	**12.9**	—	**48**	**2.4**	—
			GBM	91.7	25.4	1.2E-09	0	—	—
			MLP	91.7	32.9	5.9E-13	0	—	—
			LSTM	72.6	22.1	4.7E-06	0	—	—
**folic acid**	844	1823.5	**LR**	**100.4**	**13.8**	—	**34**	**2.4**	—
			GBM	100.4	27.7	1.2E-13	0	—	—
			MLP	100.3	31.1	1.7E-18	0	—	—
			LSTM	84.5	23.4	2.2E-07	0	—	—
**ergocalciferol**	996	1899.7	**LR**	**115.8**	**8.5**	—	**33**	**2.9**	—
			GBM	115.8	17.8	5.0E-20	0	—	—
			MLP	115.8	24	2.1E-34	0	—	—
			LSTM	100.8	18.8	1.2E-23	0	—	—

**amoxicillin**	668	2000.3	**LR**	**74.5**	**8.4**	—	**33**	**3.3**	—
			GBM	74.5	16.3	8.9E-22	0	—	—
			MLP	74.5	17	2.4E-15	0	—	—
			LSTM	69.5	28.2	2.2E-33	0	—	—
**ciprofloxacin**	619	1855.8	**LR**	**69.1**	**11.6**	—	**22**	**3.6**	—
			GBM	69.1	25.4	6.5E-27	0	—	—
			MLP	69.1	27.1	8.5E-23	0	—	—
			LSTM	66.3	30.1	1.5E-22	0	—	—
**tramadol**	645	1935	**LR**	**78.7**	**10.8**	—	**22**	**3.5**	—
			GBM	78.7	28.2	1.0E-37	0	—	—
			MLP	78.7	28.5	3.9E-18	0	—	—
			LSTM	53.3	24	7.2E-26	0	—	—
**ibuprofen**	891	2664.7	**LR**	**106.9**	**11.1**	—	**21**	**3.6**	—
			GBM	106.9	26.4	9.3E-26	0	—	—
			MLP	106.9	26.7	2.9E-20	0	—	—
			LSTM	91.2	26.5	5.5E-27	0	—	—
**citalopram**	516	1547.6	**LR**	**75.6**	**16**	—	**18**	**3.7**	—
			GBM	75.6	30.7	1.3E-17	0	—	—
			MLP	75.6	26	6.9E-08	0	—	—
			LSTM	55.1	25.1	3.8E-07	0	—	—

[a]Baseline covariates included age, sex, diagnoses codes, medications, and the time from the MCI initiation date to the trial drug initiation date, in total 267 covariates. A covariate is assumed balanced if its standardized mean difference (SMD) of its prevalence between exposure groups is at most 0.1 and a trial is assumed balanced if the ratio of unbalanced features among all covariates before/after IPTW ≤ 2%. [b] Inverse probability of treatment weights (IPTW) was estimated by different machine learning-propensity score models (ML-PS), including regularized logistic regression-based PS model (LR), gradient boosting machines (GBM), deep multilayer perceptrons (MLP), and long short-term memory neural network with attention mechanisms-based PS model (LSTM) using our proposed model selection strategy which achieved the best balancing performance. [c] Control groups are constructed randomly, either from alternative drug cohorts or similar drug cohorts under ATC-L2. We set the number of patients in the control group as 3 folds as the treated group. [d] The LSTM-based PS method estimated the number of unbalanced features by summing diagnosis and medication sequences weighted by normalized temporal attention learned from training sets, trying to summarize baseline covariates by their importance over time. [e] T-test was used for comparing the means of unbalanced covariates after re-weighting by different models (LR v.s. GBM, LR vs. MLP, LR vs. LSTM).

Supplementary Table S3. Selected ICD-9/10 diagnosis codes for Mild Cognitive Impairment (MCI) and Alzheimer's Disease (AD).

**MCI**	**Usage**: The definition of MCI in real-world healthcare data for the selection of the targeted population.<br><br>**ICD-9 codes**:<br>331.83 Mild cognitive impairment, so stated<br>294.9 Unspecified persistent mental disorders due to conditions classified elsewhere<br>**ICD-10 codes**:<br>G31.84 Mild cognitive impairment, so stated<br>F09 Unspecified mental disorder due to known physiological condition<br>To select patients with any of the above codes in the database
**AD**	**Usage**: The definition of AD in real-world healthcare data for the selection of eligible individuals before baseline and identification of outcome in follow-up.<br><br>**ICD-9 codes**:<br>331.0 Alzheimer's disease<br>**ICD-10 codes**:<br>G30 Alzheimer's disease<br>G30.0 Alzheimer's disease with early onset<br>G30.1 Alzheimer's disease with late onset<br>G30.8 Other Alzheimer's disease<br>G30.9 Alzheimer's disease, unspecified<br>To select patients with any of the above codes in the database
**AD-related dementias**	**Usage**: The definition of AD-related dementias in real-world healthcare data for selection of eligible individuals before baseline.<br><br>**ICD-9 codes**:<br>294.10 Dementia in conditions classified elsewhere without behavioral disturbance<br>294.11 Dementia in conditions classified elsewhere with behavioral disturbance<br>294.20 Dementia, unspecified, without behavioral disturbance.<br>294.21 Dementia, unspecified, with behavioral disturbance 290.* Dementias<br>**ICD-10 codes**:<br>F01.* Vascular dementia<br>F02.* Dementia in other diseases classified elsewhere F03.* Unspecified dementia<br>To select patients with any of the above codes in the database

MCI, mild cognitive impairment; AD, Alzheimer's disease; ICD-9/10, the International Classification of Diseases 9th or 10th Revision

Supplementary Table S4. Baseline comorbidity ICD codes (at the end due to size).

Supplementary Table S5-Part (a)-Trial characteristics and estimated effects from the OneFlorida –2-year follow-up period

Drug	RxNorm	Balanced Trials %	No. of treated	No. of control	No. of unbalanced feat.	No. of unbalanced feat. 95% CI	No. of unbalanced feat. after re-weighting	No. of unbalanced feat. after re-weighting, 95% CI	Adjusted 2-yr survival difference	Adjusted 2-yr survival difference, 95% CI	Adjusted 2-yr survival difference, p-value	Adjusted Hazard Ratio	Adjusted Hazard Ratio, 95% CI	Adjusted Hazard Ratio, p-value
meloxicam	41493	59	675	2025.0	72.6	70.1,75.7	0.6	0.2,0.9	2.4	2.3,2.4	0	0.50	0.49,0.52	0
pantoprazole	40790	100	1100	2508.0	114.6	112.2,117.3	0.1	0.0,0.2	2.4	2.2,2.5	0	0.58	0.56,0.60	0
mupirocin	42372	89	514	1542.0	91.0	88.0,94.4	1.7	1.4,2.0	1.5	1.4,1.6	0	0.61	0.59,0.62	0
gabapentin	25480	100	1237	2426.7	107.0	101.5,112.4	0.3	0.2,0.4	1.9	1.7,2.1	0	0.62	0.60,0.64	0
loratadine	28889	55	534	1242.9	68.1	66.9,70.1	2.3	2.1,2.6	2.3	2.2,2.4	0.016	0.63	0.61,0.65	0.038
escitalopram	3E+05	56	767	2301.0	47.7	44.1,51.1	0.4	0.2,0.7	2.9	2.3,3.6	0	0.70	0.63,0.79	0
hydrochlorothiazide	5487	18	676	2022.7	78.9	71.6,86.1	3.3	2.6,4.1	1.6	1.1,2.2	0	0.72	0.65,0.79	0
atorvastatin	83367	99	1674	3163.1	67.7	64.5,70.6	0.6	0.5,0.9	1.4	1.3,1.5	0	0.77	0.76,0.79	0
acetaminophen	161	100	1837	3376.3	50.6	49.0,52.2	2.5	2.0,3.0	1.3	1.2,1.4	0	0.78	0.76,0.79	0
albuterol	435	88	1045	2083.2	83.4	81.2,85.8	0.9	0.6,1.2	1.0	0.9,1.2	0	0.80	0.79,0.82	0
diclofenac	3355	15	1045	3129.3	122.4	117.9,126.7	3.5	2.6,4.2	1.0	0.5,1.7	0	0.81	0.73,0.88	0
lisinopril	29046	89	950	2165.2	52.3	49.9,54.8	1.4	1.0,1.8	0.9	0.8,1.0	0	0.82	0.80,0.84	0
prednisone	8640	62	572	1714.5	71.5	68.7,74.8	3.0	2.6,3.4	0.6	0.4,0.7	0	0.91	0.87,0.95	0.004
fluticasone	41126	87	903	2703.8	87.5	84.6,90.4	1.5	1.2,1.8	0.6	0.4,0.9	0	0.91	0.88,0.95	0
ergocalciferol	4018	92	996	1805	116.3	112.1,12	0.9	0.6,1.2	0.7	0.5,1.0	0	0.92	0.88,0.	0.008

				.1		0.0							97	
mirtazapine	15996	92	810	2428.8	71.2	66.4,76.2	0.6	0.4,0.8	1.4	0.9,2.0	0	0.93	0.85,1.01	0.082
omeprazole	7646	97	917	2100.4	90.8	88.7,92.8	3.0	2.7,3.4	0.3	0.2,0.5	0	0.93	0.91,0.95	0
polyethylene glycol 3350	2E+05	70	592	1776.0	113.3	108.7,118.0	2.1	1.8,2.5	0.5	0.3,0.8	0	0.94	0.88,1.00	0.118
famotidine	4278	100	842	2461.7	96.5	93.3,99.6	0.3	0.1,0.4	0.3	0.1,0.4	0	0.95	0.92,0.98	0.002
losartan	52175	60	801	2228.0	72.4	71.1,73.6	1.4	1.2,1.7	-0.0	-0.2,0.2	0.942	0.95	0.92,0.98	0
amoxicillin	723	75	668	1999.1	70.4	67.4,73.3	1.8	1.4,2.1	0.4	0.2,0.6	0	0.95	0.92,0.99	0.016
ciprofloxacin	2551	74	619	1855.3	62.9	60.3,65.5	2.3	1.9,2.6	0.2	0.1,0.4	0.006	0.98	0.94,1.01	0.168
tramadol	10689	62	645	1935.0	69.4	64.0,74.6	1.2	0.9,1.6	0.4	0.1,0.8	0.006	0.98	0.93,1.03	0.388
ibuprofen	5640	66	891	2662.7	102.3	99.1,105.4	2.9	2.5,3.3	0.5	0.2,0.7	0	0.99	0.94,1.03	0.468
trazodone	10737	99	1126	3370.8	92.5	87.4,97.4	0.9	0.7,1.1	1.1	0.5,1.7	0	1.00	0.92,1.09	0.976
sertraline	36437	94	709	2127.0	56.1	51.1,61.2	0.9	0.6,1.1	0.9	0.4,1.5	0.002	1.03	0.96,1.12	0.432
methylprednisolone	6902	17	567	1701.0	103.1	98.9,107.2	3.2	2.7,3.8	-0.5	-0.6,-0.3	0	1.05	1.02,1.09	0.008
citalopram	2556	45	516	1548.0	49.9	45.8,55.0	1.3	0.9,1.8	-0.0	-0.6,0.6	0.886	1.08	0.99,1.17	0.094
folic acid	4511	49	844	2532.0	66.3	62.9,69.8	0.7	0.3,1.0	-0.4	-0.7,-0.1	0.062	1.10	1.04,1.16	0
amlodipine	17767	98	930	2788.9	52.3	50.8,53.9	1.5	1.3,1.7	-0.3	-0.4,-0.1	0.004	1.11	1.08,1.13	0
azithromycin	18631	18	648	1944.0	79.7	74.7,84.8	3.1	2.3,3.8	-0.6	-1.0,-0.1	0.066	1.17	1.06,1.28	0.004
sodium chloride	9863	49	1000	1013.0	113.0	113.0,113.0	5.0	5.0,5.0	-1.3	-1.4,-1.3	0	1.23	1.22,1.24	0
metoprolol	6918	100	892	1682.5	60.5	58.0,62.7	0.5	0.3,0.6	-0.8	-1.0,-0.6	0	1.29	1.24,1.34	0

diphenhydramine	3498	90	581	1743.0	73.3	71.5,75.5	2.7	2.5,3.0	-1.5	-1.7,-1.4	0	1.34	1.30,1.39	0
aspirin	1191	100	1532	4576.4	50.9	49.2,52.6	0.0	0.0,0.1	-1.6	-1.7,-1.4	0	1.35	1.31,1.39	0
lidocaine	6387	23	1093	3252.4	93.2	90.7,95.7	3.5	2.9,4.0	-2.2	-2.4,-2.0	0	1.37	1.30,1.42	0
quetiapine	51272	20	618	1854.0	67.7	59.5,76.9	1.9	1.1,2.8	-2.9	-3.2,-2.4	0	1.82	1.70,1.92	0

1. adjusted hazard ratio and 2-year standardized AD-free survival differences and hazard ratios after inverse probability of treatment re-weighting (IPTW) by regularized logistic regression-based PS model (LR-PS) using our proposed model selection strategy, adjusted for 267 covariates in total: age, sex, comorbidities, medication history, and the time from MCI initiation date to the trial drug initiation date. Covariates were collected during baseline period.
2. Drugs were ranked by the estimated aHR.
3. We selected drugs with at least 10% emulated trials were balanced and for each balanced trial all the unbalanced features were balanced after IPTW.
4. Control groups are constructed randomly, either from alternative drug cohorts or similar drug cohorts under ATC L2. We set number of patients in the control group to maximum 3-folds as the treated group and we report the mean number of all balanced trials here.
5. All statistics were sample means over balanced trials. Bootstrapped p-values for one-sample T-test and 1,000 bootstrapped 95% confidence interval were reported here.

Supplementary Table S5-Part (b)-Trial characteristics and estimated effects from the OneFlorida –5-year follow-up period

Drug	RxNorm	Balanced Trials %	No. of treated	No. of control	No. of unbalanced feat.	No. of unbalanced feat. 95% CI	No. of unbalanced feat. after re-weighting	No. of unbalanced feat. after re-weighting, 95% CI	Adjusted 2-yr survival difference	Adjusted 2-yr survival difference, 95% CI	Adjusted 2-yr survival difference, p-value	Adjusted Hazard Ratio	Adjusted Hazard Ratio, 95% CI	Adjusted Hazard Ratio, p-value
escitalopram	321988	56	767	2301.0	47.7	44.1,51.1	0.4	0.2,0.7	2.9	2.3,3.6	0.193831	0.68	0.61,0.75	0
mupirocin	42372	89	514	1542.0	91.0	88.0,94.4	1.7	1.4,2.0	1.5	1.4,1.6	0.243111	0.71	0.69,0.73	0
atorvastatin	83367	99	1674	3163.1	67.7	64.5,70.6	0.7	0.5,0.9	1.4	1.3,1.5	0.166618	0.74	0.73,0.76	0
meloxicam	41493	59	675	2025.0	72.6	70.1,75.7	0.6	0.2,0.9	2.4	2.3,2.4	0.032791	0.76	0.74,0.77	0
gabapentin	25480	100	1237	2426.7	107.0	101.5,112.4	0.3	0.2,0.4	1.9	1.7,2.1	0.069958	0.76	0.74,0.77	0
polyethylene glycol 3350	221147	70	592	1776.0	113.3	108.7,118.0	2.1	1.8,2.5	0.5	0.3,0.8	0.564284	0.79	0.75,0.83	0
diclofenac	3355	15	1045	3129.3	122.4	118.0,126.9	3.5	2.7,4.2	1.0	0.5,1.7	0.428421	0.79	0.72,0.85	0
pantoprazole	40790	100	1100	2508.0	114.6	112.2,117.3	0.1	0.0,0.2	2.4	2.2,2.5	0.024904	0.81	0.79,0.83	0
mirtazapine	15996	92	810	2428.8	71.2	66.4,76.2	0.6	0.4,0.8	1.4	0.9,2.0	0.172883	0.83	0.77,0.89	0
hydrochlorothiazide	5487	18	676	2022.7	78.9	71.6,86.1	3.3	2.6,4.1	1.6	1.1,2.2	0.278641	0.84	0.76,0.91	0
trazodone	10737	99	1126	3370.8	92.5	87.4,97.4	0.9	0.7,1.1	1.1	0.5,1.7	0.05781	0.85	0.78,0.91	0
loratadine	28889	55	534	1242.9	68.1	66.9,70.1	2.3	2.1,2.6	2.3	2.2,2.4	0.111824	0.86	0.85,0.88	0.014
ibuprofen	5640	66	891	2662.7	102.3	99.1,105.5	2.9	2.5,3.3	0.5	0.2,0.7	0.578669	0.86	0.83,0.89	0
omeprazole	7646	97	917	2100.4	90.8	88.7,92.8	3.1	2.7,3.4	0.3	0.2,0.5	0.722552	0.86	0.84,0.88	0

lisinopril	29046	89	950	2165.2	52.3	49.9,54.9	1.4	1.0,1.7	0.9	0.8,1.0	0.3936	0.87	0.85,0.90	0
ergocalciferol	4018	92	996	1805.1	116.3	112.2,119.9	0.9	0.6,1.2	0.7	0.4,1.0	0.296691	0.88	0.83,0.93	0
acetaminophen	161	100	1837	3376.3	50.6	49.0,52.2	2.5	2.0,3.0	1.3	1.2,1.4	0.17726	0.89	0.87,0.91	0
fluticasone	41126	87	903	2703.8	87.5	84.5,90.3	1.5	1.2,1.8	0.6	0.4,0.9	0.547167	0.92	0.89,0.95	0
famotidine	4278	100	842	2461.7	96.5	93.3,99.6	0.3	0.1,0.4	0.3	0.1,0.4	0.554006	0.95	0.93,0.97	0
diphenhydramine	3498	90	581	1743.0	73.3	71.5,75.5	2.7	2.5,3.0	-1.5	-1.7,-1.4	0.227486	0.95	0.92,0.98	0.002
azithromycin	18631	18	648	1944.0	79.7	74.7,84.8	3.1	2.3,3.8	-0.6	-1.0,-0.1	0.443563	0.98	0.91,1.04	0.448
losartan	52175	60	801	2228.0	72.4	71.1,73.6	1.4	1.2,1.7	-0.0	-0.2,0.2	0.702365	0.99	0.97,1.01	0.31
amoxicillin	723	75	668	1999.1	70.4	67.4,73.3	1.8	1.4,2.1	0.4	0.2,0.6	0.581504	1.01	0.98,1.04	0.414
methylprednisolone	6902	17	567	1701.0	103.1	98.9,107.2	3.2	2.7,3.8	-0.5	-0.6,-0.3	0.671286	1.02	0.99,1.06	0.284
ciprofloxacin	2551	74	619	1855.3	62.9	60.3,65.5	2.3	1.9,2.6	0.2	0.1,0.4	0.642697	1.03	0.99,1.06	0.128
tramadol	10689	62	645	1935.0	69.4	64.0,74.6	1.2	0.9,1.6	0.4	0.1,0.8	0.640657	1.05	1.00,1.09	0.07
prednisone	8640	62	572	1714.5	71.5	68.7,74.8	3.0	2.6,3.4	0.6	0.4,0.7	0.554269	1.06	1.02,1.10	0
sertraline	36437	94	709	2127.0	56.1	51.1,61.2	0.9	0.6,1.1	0.9	0.4,1.5	0.243625	1.07	1.00,1.14	0.07
folic acid	4511	49	844	2532.0	66.3	62.9,69.8	0.7	0.3,1.0	-0.4	-0.7,-0.1	0.446613	1.09	1.04,1.14	0.004
albuterol	435	88	1045	2083.2	83.4	81.3,85.6	0.9	0.6,1.2	1.0	0.9,1.1	0.304436	1.09	1.07,1.10	0
metoprolol	6918	100	892	1682.5	60.5	58.0,62.7	0.5	0.3,0.6	-0.8	-1.0,-0.6	0.390861	1.09	1.07,1.11	0
sodium chloride	9863	49	1000	1013.0	113.0	113.0,113.0	5.0	5.0,5.0	-1.3	-1.4,-1.3	0.261855	1.15	1.15,1.15	0
citalopram	2556	45	516	1548.0	49.9	45.8,55.0	1.3	0.9,1.8	-0.0	-0.6,0.6	0.344022	1.19	1.10,1.29	0

amlodipine	17767	98	930	2788.9	52.3	50.8,53.8	1.5	1.3,1.7	-0.3	-0.4,-0.1	0.570642	1.23	1.21,1.26	0
aspirin	1191	100	1532	4576.4	50.9	49.2,52.6	0.0	0.0,0.1	-1.6	-1.7,-1.4	0.13925	1.27	1.23,1.30	0
lidocaine	6387	24	1093	3253.5	93.2	90.5,95.8	3.5	3.0,4.0	-2.2	-2.5,-2.0	0.05124	1.34	1.30,1.39	0
quetiapine	51272	20	618	1854.0	67.7	59.5,76.9	1.9	1.1,2.8	-2.9	-3.2,-2.4	0.061453	1.63	1.52,1.71	0

1. adjusted hazard ratio and 5-year standardized AD-free survival differences and hazard ratios after inverse probability of treatment re-weighting (IPTW) by regularized logistic regression-based PS model (LR-PS) using our proposed model selection strategy, adjusted for 267 covariates in total: age, sex, comorbidities, medication history, and the time from MCI initiation date to the trial drug initiation date. Covariates were collected during baseline period.
2. Drugs were ranked by the estimated aHR.
3. We selected drugs with at least 10% emulated trials were balanced and for each balanced trial all the unbalanced features were balanced after IPTW.
4. Control groups are constructed randomly, either from alternative drug cohorts or similar drug cohorts under ATC L2. We set number of patients in the control group to maximum 3-folds as the treated group and we report the mean number of all balanced trials here.
5. All statistics were sample means over balanced trials. Bootstrapped p-values for one-sample T-test and 1,000 bootstrapped 95% confidence interval were reported here.

Supplementary Table S6-Part (a)-Trial characteristics and estimated effects from the MarketScan –2-year follow-up period

Drug	GPI	Balanced Trials %	No. of treated	No. of control	No. of unbalanced feat.	No. of unbalanced feat. 95% CI	No. of unbalanced feat. after re-weighting	No. of unbalanced feat. after re-weighting, 95% CI	Adjusted 2-yr survival difference	Adjusted 2-yr survival difference, 95% CI	Adjusted 2-yr survival difference, p-value	Adjusted Hazard Ratio	Adjusted Hazard Ratio, 95% CI	Adjusted Hazard Ratio, p-value
lyrica	72600057	74	1786	5356.9	80.0	74.5,85.5	0.3	0.2,0.4	2.8	2.7,3.0	0	**0.57**	0.55,0.58	0
fluconazole	11407015	45	1933	5799.0	85.2	78.7,91.8	0.3	0.1,0.4	3.2	2.7,4.0	0	**0.63**	0.60,0.65	0
diazepam	57100040	82	1702	5105.8	68.3	64.0,72.9	0.2	0.1,0.3	3.0	2.8,3.3	0	**0.63**	0.61,0.66	0
chlorhexidine	88150020	88	1442	2917.5	66.6	61.5,72.1	0.1	0.0,0.1	2.4	2.3,2.6	0	**0.64**	0.62,0.65	0
hydroxyzine	57200040	88	1561	4683.0	100.2	97.0,103.1	0.1	0.0,0.2	3.0	2.6,3.3	0	**0.67**	0.64,0.70	0
azithromycin	03400010	50	5414	16190.0	37.1	34.1,40.1	0.0	0.0,0.0	3.7	2.8,4.7	0	**0.68**	0.64,0.71	0
aspirin	65990002	97	3412	10230.9	48.5	46.0,51.0	0.1	0.0,0.1	2.5	2.3,2.7	0	**0.68**	0.67,0.69	0
hydrocortisone	90550075	82	890	2670.0	30.6	26.8,34.2	0.1	0.1,0.2	2.4	2.1,2.7	0	**0.71**	0.69,0.73	0
methylprednisolone	22100030	97	3032	6971.8	78.6	76.0,81.3	0.1	0.0,0.1	1.9	1.6,2.2	0	**0.72**	0.70,0.73	0
gabapentin	72600030	100	7625	15740.1	79.0	75.2,82.5	0.0	0.0,0.0	2.6	2.2,3.2	0	**0.72**	0.70,0.74	0
bupropion	58300040	52	3773	11319.0	51.0	48.9,53.0	0.4	0.3,0.6	2.9	2.6,3.1	0	**0.75**	0.72,0.78	0
clobetasol	90550025	92	1668	5004.0	30.3	27.2,33.9	0.0	0.0,0.1	2.2	1.8,2.9	0	**0.75**	0.73,0.77	0
metronidazole	16000035	83	1171	3513.0	43.9	40.6,47.5	0.1	0.0,0.1	2.2	2.0,2.4	0	**0.76**	0.74,0.78	0
dextromethorphan	43997	67	1003	3009	21.3	17.5,25.	0.1	0.1,0.2	1.3	1.2,1.4	0	**0.76**	0.74,0	0

	002			.0		6							.78	
tizanidine	75100090	63	1648	4944.0	69.8	66.0,74.2	0.0	0.0,0.1	1.2	1.0,1.3	0	**0.76**	0.74,0.78	0
oxycodone	65100075	55	2110	6330.0	79.3	74.1,84.9	0.4	0.3,0.5	1.4	1.2,1.5	0	**0.77**	0.75,0.79	0
albuterol	44201010	99	4413	8058.3	20.9	18.8,23.3	0.0	0.0,0.0	1.9	1.5,2.3	0	**0.78**	0.75,0.80	0
Ibuprofen	65991702	100	7449	13282.4	20.9	19.8,22.1	0.0	0.0,0.0	2.4	2.0,3.0	0	**0.78**	0.75,0.80	0
amoxicillin	01200010	100	5530	9358.2	11.3	9.1,13.6	0.5	0.4,0.6	1.9	1.5,2.5	0	**0.80**	0.77,0.82	0
fluzone	17100020	36	2563	7687.6	75.3	70.2,80.5	0.3	0.1,0.4	2.2	1.0,3.8	0	**0.81**	0.76,0.86	0
ketoconazole	90154045	98	1740	5220.0	30.8	28.3,33.4	0.0	0.0,0.1	1.3	1.0,1.7	0	**0.83**	0.80,0.85	0
prednisone	22100045	100	5557	9559.0	34.1	31.2,37.5	0.5	0.4,0.6	1.7	1.2,2.3	0	**0.85**	0.81,0.88	0
metformin	27250050	52	3696	4369.7	73.4	72.4,74.0	0.0	0.0,0.1	1.4	1.4,1.4	0	**0.85**	0.85,0.85	0
methocarbamol	75100070	61	976	2928.0	54.6	50.3,59.3	0.1	0.0,0.1	0.8	0.7,1.0	0	**0.85**	0.82,0.88	0
penicillin v potassium	01100040	35	607	1821.0	38.6	34.3,43.9	0.3	0.1,0.4	0.8	0.5,1.0	0	**0.85**	0.82,0.89	0
zolpidem	60204080	97	2617	4483.0	34.4	29.3,40.1	0.0	0.0,0.1	1.2	1.0,1.4	0	**0.85**	0.83,0.88	0
doxycycline	04000020	50	3653	10959.0	47.2	42.2,52.6	0.0	0.0,0.1	1.6	0.8,2.6	0	**0.86**	0.82,0.90	0
amitriptyline	58200010	81	1546	4638.0	84.3	79.6,89.0	0.3	0.2,0.4	1.6	1.1,2.0	0	**0.86**	0.81,0.91	0
spironolactone	37500020	62	2058	6174.0	49.5	46.8,52.7	0.1	0.0,0.2	1.9	1.8,2.1	0	**0.87**	0.85,0.88	0
januvia	27550070	23	1271	3813.0	25.7	23.8,27.6	0.1	0.0,0.2	0.9	0.7,1.1	0	**0.87**	0.84,0.89	0
fluticasone	42200032	100	5500	9071.8	18.4	15.4,21.7	0.0	0.0,0.0	1.5	1.0,2.0	0	**0.87**	0.84,0.90	0
myrbetriq	54200050	50	1524	4572.0	84.1	83.5,84.7	0.0	0.0,0.0	1.8	1.7,1.9	0	**0.87**	0.87,0.88	0

triamcinolone	90550085	99	2847	8541.0	17.3	15.0,20.0	0.0	0.0,0.0	1.2	0.9,1.5	0	**0.88**	0.86,0.89	0
atorvastatin	39400010	100	9161	18586.5	26.1	24.4,27.8	0.3	0.2,0.4	1.2	0.8,1.6	0	**0.89**	0.86,0.92	0
cefuroxime	02200065	87	1124	3372.0	48.9	46.1,51.7	0.4	0.3,0.5	1.1	1.0,1.3	0	**0.89**	0.87,0.91	0
valsartan	36150080	55	1061	3183.0	25.7	22.7,29.2	0.2	0.1,0.3	0.9	0.7,1.2	0	**0.90**	0.87,0.92	0
duloxetine	58180025	91	3940	11820.0	77.1	74.5,79.9	0.4	0.3,0.5	1.5	1.0,1.9	0	**0.90**	0.85,0.95	0
childrens Ibuprofen	66100020	90	2244	6732.0	37.9	31.2,44.3	0.1	0.0,0.1	1.1	0.8,1.5	0	**0.91**	0.88,0.93	0
prednisolone	86300050	58	2543	7629.0	9.7	8.1,11.7	0.3	0.2,0.4	1.0	0.7,1.5	0	**0.91**	0.88,0.93	0
omeprazole	49270060	100	6966	14832.8	8.3	7.2,9.6	0.0	0.0,0.0	1.0	0.6,1.6	0	**0.91**	0.88,0.94	0
levofloxacin	05000034	100	3393	7214.5	52.9	50.1,56.3	0.5	0.4,0.6	1.3	0.9,1.9	0	**0.91**	0.89,0.94	0
rivaroxaban	83370060	54	1699	5097.0	30.3	28.8,32.5	0.1	0.0,0.1	0.1	0.0,0.1	0.012	**0.92**	0.91,0.92	0
penicillin	01990002	100	3808	7787.3	45.4	42.0,48.8	0.5	0.4,0.6	0.9	0.5,1.5	0	**0.92**	0.89,0.95	0
furosemide	37200030	98	6067	11483.3	69.4	66.0,72.9	0.3	0.2,0.4	1.2	0.7,1.7	0	**0.92**	0.90,0.95	0
pantoprazole	49270070	100	5555	14580.5	41.2	38.7,44.3	0.0	0.0,0.0	1.2	0.8,1.7	0	**0.93**	0.90,0.95	0
nystatin	88100010	46	736	2208.0	98.2	93.6,102.3	0.5	0.3,0.6	0.7	0.6,0.9	0	**0.94**	0.92,0.96	0
metoprolol	33200030	100	6825	12359.0	38.9	34.9,42.5	0.5	0.4,0.6	1.0	0.6,1.5	0	**0.94**	0.91,0.97	0
diclofenac	66100007	60	1239	3717.0	26.6	22.2,31.4	0.1	0.0,0.2	0.4	0.3,0.7	0	**0.94**	0.92,0.97	0
tramadol	65100095	100	5544	14046.5	37.9	35.2,40.7	0.0	0.0,0.0	0.8	0.4,1.4	0	**0.94**	0.91,0.98	0
mupirocin	90100065	99	2165	6495.0	37.7	33.5,42.3	0.0	0.0,0.0	0.6	0.2,1.0	0	**0.95**	0.92,0.97	0
fluocinonide	90550060	86	956	2868.0	27.3	23.8,30.9	0.2	0.1,0.2	0.7	0.5,1.0	0	**0.95**	0.92,0.98	0

amlodipine	34000003	80	6837	8790.2	31.4	28.3,35.0	0.4	0.3,0.5	1.1	0.6,1.7	0	**0.95**	0.91,0.98	0
celecoxib	66100525	84	1586	4758.0	39.8	35.5,44.7	0.1	0.1,0.2	0.3	0.0,0.6	0.008	**0.96**	0.93,0.98	0
clotrimazole	90159902	99	1651	4953.0	21.1	18.0,24.7	0.1	0.0,0.1	0.5	0.3,0.9	0	**0.96**	0.93,0.98	0
ropinirole	73203070	84	1136	3408.0	85.8	78.9,92.4	0.0	0.0,0.1	0.5	0.4,0.7	0	**0.96**	0.94,0.98	0
losartan	36150040	84	5084	14360.3	31.2	29.4,33.0	0.4	0.3,0.5	0.5	0.1,0.9	0.002	**0.96**	0.93,0.98	0
pravastatin	39400065	100	3334	10000.7	21.2	17.0,25.5	0.0	0.0,0.1	0.3	-0.1,0.9	0.104	**0.96**	0.93,0.99	0.002
amphetamine-dextroamphetamine	61109902	50	1265	2732.0	38.0	38.0,38.0	0.0	0.0,0.0	0.0	0.0,0.0	0	**0.96**	0.96,0.96	0
levothyroxine	28100010	50	4167	12497.4	35.0	30.8,39.4	0.0	0.0,0.0	0.9	0.3,1.8	0.002	**0.96**	0.92,1.01	0.094
hydrochlorothiazide	37600040	99	3430	9479.9	49.4	46.6,52.1	0.6	0.5,0.7	0.2	-0.0,0.6	0.062	**0.97**	0.94,0.99	0.002
venlafaxine	58180090	81	2497	7491.0	41.2	36.2,45.8	0.3	0.2,0.4	0.7	0.2,1.1	0.008	**0.98**	0.92,1.04	0.48
clindamycin	16220020	97	1963	5888.5	33.5	30.7,36.6	0.1	0.0,0.1	0.2	-0.1,0.5	0.2	**0.98**	0.96,1.00	0.022
clopidogrel	85158020	50	3628	753.0	18.0	18.0,18.0	0.0	0.0,0.0	-0.1	-0.1,-0.1	0	**0.98**	0.98,0.98	0
carvedilol	33300007	55	2733	8199.0	62.1	60.8,63.3	0.1	0.0,0.1	-0.1	-0.2,-0.0	0.012	**0.99**	0.98,1.00	0.21
naproxen	66100060	73	1850	5550.0	24.0	18.1,30.0	0.1	0.0,0.2	0.2	-0.2,0.6	0.374	**0.99**	0.96,1.02	0.598
ergocalciferol	77202030	50	3703	11109.0	19.6	16.0,24.6	0.0	0.0,0.0	0.6	-0.2,1.6	0.12	**0.99**	0.94,1.04	0.722
lidocaine	90850060	98	2116	6348.0	80.3	76.9,83.8	0.1	0.0,0.1	0.5	0.3,0.9	0	**1.00**	0.98,1.02	0.988
oxybutynin	54100045	99	2409	5411.3	22.3	18.2,26.5	0.1	0.1,0.2	0.3	-0.1,0.6	0.15	**1.00**	0.97,1.03	0.98
warfarin	83200030	77	2764	4849.8	75.1	71.4,78.9	0.1	0.1,0.2	0.3	0.2,0.4	0	**1.00**	0.99,1.01	0.96
rosuvastatin	39400060	94	2663	7989.0	31.9	28.9,34.8	0.4	0.3,0.4	-0.1	-0.2,0.0	0.076	**1.00**	0.99,1.02	0.69

ondansetron	50250065	98	3175	5263.9	88.6	86.3,91.0	0.1	0.0,0.1	-0.4	-0.7,-0.0	0.134	**1.02**	0.99,1.04	0.322
cephalexin	02100020	100	5106	8335.5	29.6	28.1,31.2	0.0	0.0,0.0	-0.0	-0.4,0.5	0.95	**1.02**	0.99,1.05	0.208
ranitidine	49200020	99	2434	7302.0	48.8	44.5,53.8	0.0	0.0,0.0	-0.3	-0.6,0.1	0.216	**1.03**	1.00,1.05	0.072
rivastigmine	62051040	49	2588	7764.0	14.2	12.4,17.3	0.5	0.4,0.7	-1.6	-2.3,-1.2	0	**1.04**	1.02,1.07	0
apixaban	83370010	50	2051	5653.0	81.0	81.0,81.0	0.0	0.0,0.0	-0.6	-0.6,-0.6	0	**1.04**	1.04,1.04	0
meloxicam	66100052	91	4041	8974.6	33.9	28.9,39.2	0.0	0.0,0.1	-0.1	-0.5,0.5	0.792	**1.05**	1.02,1.08	0.028
sulfamethoxazole-trimethoprim	16990002	100	4219	8407.8	16.1	14.2,18.3	0.0	0.0,0.0	-0.2	-0.5,0.3	0.576	**1.06**	1.03,1.08	0
trazodone	58120080	100	4782	14346.0	42.6	39.5,45.6	0.0	0.0,0.0	0.2	-0.3,0.7	0.424	**1.06**	1.00,1.11	0.016
cyclobenzaprine	75100050	67	2694	4246.2	34.5	26.8,43.2	0.3	0.2,0.3	-0.3	-0.4,-0.2	0.004	**1.06**	1.05,1.08	0.002
clonazepam	72100010	97	2791	8373.0	71.5	67.6,75.4	0.1	0.1,0.2	-0.1	-0.4,0.3	0.696	**1.06**	1.04,1.09	0.002
ciprofloxacin	05000020	100	5749	9534.2	19.5	18.4,20.7	0.5	0.4,0.6	-0.5	-0.9,0.0	0.114	**1.07**	1.03,1.10	0.002
lisinopril	36100030	99	6430	14583.9	43.7	38.6,49.5	0.4	0.3,0.5	-0.5	-0.9,0.0	0.202	**1.07**	1.04,1.10	0
fluticasone-salmeterol	44209902	50	3245	5516.0	23.0	23.0,23.0	0.0	0.0,0.0	-0.1	-0.1,-0.1	0	**1.07**	1.07,1.07	0
diclofenac	90210030	96	2225	6675.0	67.4	63.6,71.5	0.3	0.2,0.4	-0.1	-0.5,0.3	0.64	**1.07**	1.04,1.10	0.002
laxative	46600033	92	1492	3026.5	66.5	64.6,68.2	0.1	0.1,0.2	-0.5	-0.8,-0.2	0.01	**1.08**	1.05,1.10	0
tamsulosin	56852070	50	4595	1691.0	61.0	61.0,61.0	0.0	0.0,0.0	-0.5	-0.6,-0.5	0	**1.08**	1.08,1.08	0
potassium	79700030	47	4803	14376.0	69.2	65.4,72.6	0.8	0.7,0.9	-0.4	-0.9,0.2	0.35	**1.08**	1.03,1.12	0.014
levetiracetam	72600043	16	2495	7485.0	93.3	86.5,99.8	1.0	1.0,1.0	-0.6	-0.9,-0.3	0	**1.09**	1.04,1.14	0.006
acetaminophen-codeine	65991002	99	1738	5214.0	43.1	37.9,48.1	0.1	0.1,0.2	-0.4	-0.7,-0.0	0.188	**1.10**	1.07,1.13	0

solifenacin	54100055	83	1404	4211.4	21.2	16.3,25.9	0.2	0.1,0.3	-0.9	-1.1,-0.6	0	**1.11**	1.07,1.14	0
montelukast	44505050	86	2194	6582.0	76.2	73.1,79.4	0.4	0.3,0.5	-1.3	-1.4,-1.1	0	**1.11**	1.09,1.13	0
simvastatin	39400075	100	3596	10788.0	81.5	76.1,87.0	0.0	0.0,0.0	-0.8	-1.1,-0.3	0.104	**1.11**	1.09,1.14	0
alprazolam	57100010	99	2941	7243.6	23.6	19.9,27.7	0.0	0.0,0.1	-0.8	-1.1,-0.5	0	**1.13**	1.09,1.17	0
baclofen	75100010	74	1600	4800.0	42.0	37.4,46.5	0.1	0.0,0.2	-0.3	-0.4,-0.1	0.008	**1.14**	1.10,1.17	0
benzonatate	43102010	93	2189	3978.8	51.4	48.8,54.2	0.2	0.1,0.3	-0.4	-0.8,0.0	0.168	**1.14**	1.10,1.18	0
paroxetine	58160060	87	1172	3516.0	35.6	30.5,41.1	0.1	0.1,0.2	-1.0	-1.4,-0.5	0	**1.15**	1.10,1.21	0
nitrofurantoin	16800050	52	2572	6567.9	33.3	33.0,33.8	1.0	1.0,1.0	-1.1	-1.1,-1.1	0	**1.15**	1.15,1.16	0
fluoxetine	58160040	96	2158	6474.0	40.3	35.1,45.6	0.1	0.0,0.1	-0.5	-1.0,-0.0	0.058	**1.16**	1.09,1.22	0
tolterodine	54100060	72	901	2703.0	24.3	20.5,28.2	0.2	0.1,0.2	-1.5	-1.7,-1.3	0	**1.16**	1.13,1.19	0
bystolic	33200040	16	705	2115.0	54.1	49.6,58.8	0.6	0.4,0.8	-1.4	-1.7,-1.0	0	**1.16**	1.11,1.24	0
nystatin	90150080	89	1587	4761.0	73.5	70.8,76.2	0.1	0.1,0.2	-1.2	-1.3,-1.0	0	**1.17**	1.15,1.20	0
temazepam	60201030	82	1236	3580.6	39.5	36.1,43.3	0.1	0.1,0.2	-0.8	-0.9,-0.6	0	**1.19**	1.16,1.21	0
famotidine	49200030	97	1859	5577.0	36.3	32.6,40.1	0.0	0.0,0.1	-1.8	-2.1,-1.4	0	**1.26**	1.24,1.29	0
buspirone	57200005	53	1723	5169.0	54.2	52.5,55.8	0.0	0.0,0.1	-2.6	-2.7,-2.4	0	**1.30**	1.26,1.34	0
citalopram	58160020	100	4298	12894.0	73.9	70.2,77.3	0.0	0.0,0.0	-2.3	-2.8,-1.8	0	**1.32**	1.26,1.38	0
methylphenidate	61400020	51	1171	2876.7	33.2	32.0,35.6	0.0	0.0,0.0	-0.7	-0.8,-0.7	0	**1.33**	1.32,1.34	0
mirtazapine	58030050	99	3686	11056.3	50.2	48.6,51.7	0.1	0.0,0.1	-2.7	-3.2,-2.3	0	**1.36**	1.29,1.42	0
lorazepam	57100060	100	3077	7649.0	34.3	29.2,39.5	0.0	0.0,0.0	-2.6	-3.0,-2.1	0	**1.46**	1.41,1.51	0

sertraline	58160070	100	5524	16564.6	17.6	15.5,19.7	0.0	0.0,0.0	-3.3	-3.7,-2.9	0	**1.49**	1.43,1.55	0
escitalopram	58160034	100	5041	15123.0	13.5	11.0,15.8	0.0	0.0,0.0	-4.3	-4.7,-3.9	0	**1.56**	1.49,1.62	0
premarin	55350025	48	613	1232.0	31.0	31.0,31.0	1.0	1.0,1.0	-2.7	-2.7,-2.6	0	**1.57**	1.56,1.58	0
quetiapine	59153070	94	2934	6345.1	38.6	34.0,43.5	0.1	0.1,0.2	-6.0	-6.7,-5.3	0	**1.84**	1.71,1.99	0
memantine	62053550	100	6282	18151.7	57.3	47.0,66.8	1.0	0.9,1.0	-10.2	-12.1,-8.2	0	**2.29**	2.04,2.56	0
donepezil	62051025	100	15503	26179.9	98.5	91.8,104.7	0.5	0.4,0.6	-13.9	-16.0,-11.8	0	**3.50**	3.02,3.99	0

1. adjusted hazard ratio and 2-year standardized AD-free survival differences and hazard ratios after inverse probability of treatment re-weighting (IPTW) by regularized logistic regression-based PS model (LR-PS) using our proposed model selection strategy, adjusted for 267 covariates in total: age, sex, comorbidities, medication history, and the time from MCI initiation date to the trial drug initiation date. Covariates were collected during baseline period.
2. Drugs were ranked by the estimated aHR.
3. We selected drugs with at least 10% emulated trials were balanced and for each balanced trial all the unbalanced features were balanced after IPTW.
4. Control groups are constructed randomly, either from alternative drug cohorts or similar drug cohorts under ATC L2. We set number of patients in the control group to maximum 3-folds as the treated group and we report the mean number of all balanced trials here.
5. All statistics were sample means over balanced trials. Bootstrapped p-values for one-sample T-test and 1,000 bootstrapped 95% confidence interval were reported here.

Supplementary Table S6-Part (b)-Trial characteristics and estimated effects from the MarketScan –5-year follow-up period

Drug	GPI	Balanced Trials %	No. of treated	No. of control	No. of unbalanced feat.	No. of unbalanced feat. 95% CI	No. of unbalanced feat. after re-weighting	No. of unbalanced feat. after re-weighting, 95% CI	Adjusted 2-yr survival difference	Adjusted 2-yr survival difference, 95% CI	Adjusted 2-yr survival difference, p-value	Adjusted Hazard Ratio	Adjusted Hazard Ratio, 95% CI	Adjusted Hazard Ratio, p-value
lyrica	72600057	73	1786	5356.8	79.5	74.4,85.1	0.3	0.2,0.4	3.1	2.9,3.2	0.001361	0.53	0.51,0.54	0
diazepam	57100040	82	1702	5105.8	68.3	63.9,72.6	0.2	0.1,0.3	2.9	2.7,3.1	0.030023	0.69	0.67,0.71	0
fluconazole	11407015	45	1933	5799.0	85.2	78.6,91.8	0.3	0.1,0.4	3.1	2.6,3.7	0.010144	0.71	0.69,0.74	0
aspirin	65990002	97	3412	10230.9	48.5	46.0,51.0	0.1	0.0,0.1	2.4	2.3,2.6	0.001751	0.71	0.71,0.72	0
albuterol	44201010	99	4413	8058.3	20.9	18.8,23.3	0.0	0.0,0.0	1.8	1.5,2.1	0.136659	0.72	0.71,0.73	0
tizanidine	75100090	63	1648	4944.0	69.8	66.0,74.2	0.0	0.0,0.1	1.2	1.1,1.4	0.131464	0.73	0.71,0.75	0
oxycodone	65100075	55	2110	6330.0	79.3	73.8,85.1	0.4	0.3,0.5	1.3	1.2,1.5	0.165533	0.74	0.72,0.76	0
azithromycin	03400010	50	5414	16190.0	37.1	34.1,40.1	0.0	0.0,0.0	3.4	2.7,4.2	0.000753	0.74	0.71,0.77	0
hydroxyzine	57200040	88	1561	4683.0	100.2	97.1,103.2	0.1	0.0,0.2	2.9	2.6,3.2	0.087534	0.74	0.71,0.77	0
methylprednisolone	22100030	97	3032	6971.8	78.6	76.0,81.3	0.1	0.0,0.1	1.8	1.6,2.1	0.029219	0.75	0.74,0.76	0
nystatin	88100010	46	736	2208.0	98.2	94.0,102.3	0.5	0.3,0.6	0.9	0.7,1.1	0.514076	0.75	0.73,0.77	0
bupropion	58300040	52	3773	11319.0	51.0	48.9,53.0	0.4	0.3,0.6	2.6	2.3,2.8	0.005811	0.77	0.76,0.80	0
clobetasol	90550025	92	1668	5004.0	30.3	27.3,33.7	0.0	0.0,0.1	2.1	1.7,2.7	0.094251	0.78	0.76,0.80	0
dextromethorphan	43997	67	1003	3009	21.3	17.4,25.	0.1	0.1,0.2	1.3	1.2,1.4	0.2143	0.79	0.77,0	0

	002			.0		5					69		.80	
metronidazole	16000035	83	1171	3513.0	43.9	40.5,47.6	0.1	0.0,0.1	2.3	2.1,2.5	0.09563	0.79	0.77,0.81	0
gabapentin	72600030	100	7625	15740.1	79.0	75.2,82.5	0.0	0.0,0.0	2.5	2.1,2.9	0.000806	0.79	0.77,0.81	0
hydrocortisone	90550075	82	890	2670.0	30.6	26.6,34.2	0.1	0.1,0.2	2.4	2.1,2.6	0.12137	0.79	0.77,0.81	0
Ibuprofen	65991702	100	7449	13282.4	20.9	19.8,22.1	0.0	0.0,0.0	2.3	1.9,2.7	0.036814	0.80	0.78,0.82	0
methocarbamol	75100070	61	976	2928.0	54.6	50.4,59.5	0.1	0.0,0.1	0.8	0.6,1.0	0.458302	0.81	0.79,0.83	0
fluzone	17100020	36	2563	7687.6	75.3	70.1,80.6	0.3	0.1,0.4	2.0	1.0,3.4	0.233042	0.83	0.78,0.87	0
amoxicillin	01200010	100	5530	9358.2	11.3	9.1,13.6	0.5	0.4,0.6	1.8	1.5,2.3	0.105933	0.83	0.81,0.85	0
chlorhexidine	88150020	88	1442	2917.5	66.6	61.6,72.2	0.1	0.0,0.1	2.3	2.2,2.5	0.038767	0.84	0.83,0.85	0
fluticasone	42200032	100	5500	9071.8	18.4	15.4,21.7	0.0	0.0,0.0	1.3	1.0,1.8	0.386164	0.86	0.83,0.87	0
doxycycline	04000020	50	3653	10959.0	47.2	42.2,52.6	0.0	0.0,0.1	1.4	0.8,2.3	0.308321	0.86	0.82,0.90	0
prednisone	22100045	100	5557	9559.0	34.1	31.2,37.5	0.5	0.4,0.6	1.6	1.1,2.1	0.438546	0.87	0.83,0.90	0
zolpidem	60204080	97	2617	4483.0	34.4	29.5,39.9	0.0	0.0,0.1	1.2	1.0,1.4	0.263173	0.87	0.86,0.89	0
januvia	27550070	23	1271	3813.0	25.7	23.8,27.6	0.1	0.0,0.2	0.9	0.7,1.1	0.461223	0.89	0.87,0.91	0
ketoconazole	90154045	98	1740	5220.0	30.8	28.5,33.5	0.0	0.0,0.1	1.2	1.0,1.6	0.325261	0.89	0.87,0.91	0
penicillin	01990002	100	3808	7787.3	45.4	42.0,48.8	0.5	0.4,0.6	0.8	0.5,1.3	0.515051	0.89	0.87,0.91	0
amitriptyline	58200010	81	1546	4638.0	84.3	79.6,89.0	0.3	0.2,0.4	1.5	1.1,1.9	0.238586	0.89	0.86,0.94	0
rivaroxaban	83370060	54	1699	5097.0	30.3	28.8,32.3	0.1	0.0,0.1	0.1	0.0,0.1	0.88022	0.90	0.89,0.90	0
triamcinolone	90550085	99	2847	8541.0	17.3	15.0,20.0	0.0	0.0,0.0	1.2	0.9,1.4	0.251761	0.90	0.88,0.91	0

celecoxib	66100525	84	1586	4758.0	39.8	35.4,44.5	0.1	0.1,0.2	0.2	0.0,0.5	0.629169	0.90	0.88,0.92	0
fluocinonide	90550060	86	956	2868.0	27.3	23.7,31.0	0.2	0.1,0.3	0.9	0.6,1.1	0.468424	0.90	0.88,0.92	0
furosemide	37200030	98	6067	11483.3	69.4	66.0,72.9	0.3	0.2,0.4	1.0	0.6,1.5	0.395375	0.91	0.88,0.93	0
naproxen	66100060	73	1850	5550.0	24.0	18.1,30.1	0.1	0.0,0.2	0.1	-0.2,0.6	0.633336	0.91	0.88,0.93	0
omeprazole	49270060	100	6966	14832.8	8.3	7.2,9.6	0.0	0.0,0.0	0.9	0.5,1.3	0.572858	0.91	0.89,0.93	0
myrbetriq	54200050	50	1524	4572.0	84.1	83.5,84.6	0.0	0.0,0.0	1.8	1.7,1.9	0.141679	0.92	0.91,0.92	0
childrens Ibuprofen	66100020	90	2244	6732.0	37.9	31.7,44.4	0.1	0.0,0.1	1.1	0.9,1.5	0.275149	0.92	0.90,0.93	0
duloxetine	58180025	91	3940	11820.0	77.1	74.5,79.9	0.4	0.3,0.5	1.3	0.8,1.7	0.074041	0.92	0.87,0.96	0.002
cefuroxime	02200065	87	1124	3372.0	48.9	46.2,51.7	0.4	0.3,0.5	1.1	0.9,1.3	0.352255	0.92	0.91,0.94	0
atorvastatin	39400010	100	9161	18586.5	26.1	24.4,27.8	0.3	0.2,0.4	1.1	0.7,1.4	0.2226	0.92	0.90,0.94	0
lidocaine	90850060	98	2116	6348.0	80.3	77.0,83.8	0.1	0.0,0.1	0.5	0.2,0.7	0.626115	0.92	0.90,0.94	0
valsartan	36150080	55	1061	3183.0	25.7	22.5,29.3	0.1	0.1,0.3	0.8	0.6,1.1	0.520449	0.92	0.91,0.94	0
spironolactone	37500020	62	2058	6174.0	49.5	46.9,52.4	0.1	0.0,0.2	1.9	1.8,2.0	0.082295	0.92	0.91,0.94	0
venlafaxine	58180090	81	2497	7491.0	41.2	36.2,45.8	0.3	0.2,0.4	0.7	0.2,1.1	0.088298	0.93	0.88,0.98	0.008
diclofenac	66100007	60	1239	3717.0	26.6	22.2,31.5	0.1	0.0,0.2	0.4	0.2,0.6	0.661737	0.93	0.91,0.95	0
penicillin v potassium	01100040	35	607	1821.0	38.6	34.2,44.1	0.3	0.1,0.4	0.7	0.5,1.0	0.588997	0.93	0.91,0.96	0
carvedilol	33300007	55	2733	8199.0	62.1	60.8,63.3	0.1	0.0,0.1	-0.1	-0.2,-0.0	0.821263	0.94	0.93,0.94	0
prednisolone	86300050	58	2543	7629.0	9.7	8.1,11.8	0.3	0.1,0.4	1.0	0.7,1.4	0.362796	0.94	0.92,0.96	0
pantoprazole	49270070	100	5555	14580.5	41.2	38.7,44.3	0.0	0.0,0.0	1.1	0.8,1.5	0.26386	0.94	0.92,0.96	0

ropinirole	73203070	84	1136	3408.0	85.8	79.2,92.3	0.0	0.0,0.1	0.7	0.6,0.9	0.56859	0.95	0.93,0.97	0
clopidogrel	85158020	50	3628	753.0	18.0	18.0,18.0	0.0	0.0,0.0	-0.3	-0.3,-0.2	0.85667	0.96	0.96,0.96	0
tamsulosin	56852070	50	4595	1691.0	61.0	61.0,61.0	0.0	0.0,0.0	-0.5	-0.5,-0.5	0.602962	0.96	0.96,0.96	0.002
metformin	27250050	52	3696	4369.7	73.4	72.4,74.0	0.0	0.0,0.1	1.3	1.3,1.4	0.071696	0.96	0.96,0.96	0
losartan	36150040	84	5084	14360.3	31.2	29.4,33.0	0.4	0.3,0.5	0.4	0.1,0.8	0.303184	0.97	0.94,0.99	0
amlodipine	34000003	80	6837	8790.2	31.4	28.3,35.0	0.4	0.3,0.5	1.0	0.6,1.6	0.583942	0.97	0.94,1.00	0.008
clindamycin	16220020	97	1963	5888.5	33.5	30.7,36.5	0.1	0.0,0.1	0.2	-0.1,0.4	0.657034	0.97	0.96,0.99	0
levofloxacin	05000034	100	3393	7214.5	52.9	50.1,56.3	0.5	0.4,0.6	1.1	0.7,1.6	0.280942	0.98	0.96,1.01	0.118
hydrochlorothiazide	37600040	99	3430	9479.9	49.4	46.6,52.1	0.6	0.5,0.7	0.2	-0.0,0.6	0.558041	0.99	0.97,1.01	0.136
clonazepam	72100010	97	2791	8373.0	71.5	67.6,75.4	0.1	0.1,0.2	-0.1	-0.4,0.2	0.40321	0.99	0.97,1.01	0.496
apixaban	83370010	50	2051	5653.0	81.0	81.0,81.0	0.0	0.0,0.0	-0.5	-0.5,-0.4	0.666664	0.99	0.99,1.00	0.004
trazodone	58120080	100	4782	14346.0	42.6	39.5,45.6	0.0	0.0,0.0	0.4	-0.0,0.8	0.03776	1.00	0.96,1.04	0.894
meloxicam	66100052	91	4041	8974.6	33.9	28.9,39.2	0.0	0.0,0.1	-0.2	-0.6,0.4	0.272091	1.00	0.97,1.03	0.882
levothyroxine	28100010	50	4167	12497.4	35.0	30.8,39.4	0.0	0.0,0.0	0.7	0.2,1.5	0.39624	1.01	0.96,1.04	0.838
clotrimazole	90159902	99	1651	4953.0	21.1	17.9,24.6	0.1	0.0,0.1	0.5	0.3,0.9	0.576066	1.01	0.99,1.03	0.38
rosuvastatin	39400060	94	2663	7989.0	31.9	28.9,34.8	0.4	0.3,0.4	-0.1	-0.3,-0.0	0.650485	1.01	0.99,1.03	0.156
potassium	79700030	47	4803	14376.0	69.2	65.4,72.6	0.8	0.7,0.9	-0.5	-0.9,0.1	0.156557	1.01	0.98,1.04	0.548
diclofenac	90210030	96	2225	6675.0	67.4	63.6,71.6	0.3	0.2,0.4	-0.2	-0.5,0.1	0.424454	1.01	0.99,1.04	0.272
pravastatin	39400065	100	3334	10000.7	21.2	17.0,25.5	0.0	0.0,0.1	0.3	-0.1,0.8	0.464639	1.01	0.99,1.04	0.312

metoprolol	33200030	100	6825	12359.0	38.9	34.9,42.5	0.5	0.4,0.6	0.8	0.5,1.3	0.534778	1.01	0.99,1.04	0.35
oxybutynin	54100045	99	2409	5411.3	22.3	18.6,26.6	0.1	0.1,0.2	0.2	-0.2,0.5	0.20105	1.02	0.99,1.05	0.27
cephalexin	02100020	100	5106	8335.5	29.6	28.1,31.2	0.0	0.0,0.0	-0.1	-0.5,0.3	0.35532	1.02	0.99,1.05	0.188
laxative	46600033	92	1492	3026.5	66.5	64.5,68.2	0.1	0.1,0.2	-0.5	-0.7,-0.2	0.657352	1.02	1.00,1.04	0.062
levetiracetam	72600043	16	2495	7485.0	93.3	86.1,99.3	1.0	1.0,1.0	-0.6	-0.8,-0.3	0.539101	1.02	0.99,1.06	0.188
amphetamine-dextroamphetamine	61109902	50	1265	2732.0	38.0	38.0,38.0	0.0	0.0,0.0	-0.1	-0.1,-0.1	0.891297	1.02	1.02,1.02	0
montelukast	44505050	86	2194	6582.0	76.2	72.8,79.4	0.4	0.3,0.5	-1.3	-1.4,-1.1	0.220995	1.03	1.01,1.05	0
ciprofloxacin	05000020	100	5749	9534.2	19.5	18.4,20.7	0.5	0.4,0.6	-0.6	-0.9,-0.2	0.178172	1.03	1.00,1.05	0.048
tramadol	65100095	100	5544	14046.5	37.9	35.2,40.7	0.0	0.0,0.0	0.7	0.3,1.1	0.337767	1.05	1.01,1.08	0.022
benzonatate	43102010	93	2189	3978.8	51.4	48.8,54.2	0.2	0.1,0.3	-0.4	-0.8,-0.0	0.284872	1.05	1.02,1.09	0.01
warfarin	83200030	77	2764	4849.8	75.1	71.4,78.9	0.1	0.1,0.2	0.2	0.1,0.3	0.814103	1.06	1.05,1.07	0
lisinopril	36100030	99	6430	14583.9	43.7	38.6,49.5	0.4	0.3,0.5	-0.6	-1.0,-0.2	0.129679	1.06	1.03,1.09	0
sulfamethoxazole-trimethoprim	16990002	100	4219	8407.8	16.1	14.2,18.3	0.0	0.0,0.0	-0.3	-0.6,0.0	0.266366	1.06	1.04,1.08	0
ergocalciferol	77202030	50	3703	11109.0	19.6	16.0,24.6	0.0	0.0,0.0	0.5	-0.2,1.4	0.395886	1.07	1.02,1.11	0.064
mupirocin	90100065	99	2165	6495.0	37.7	33.6,42.1	0.0	0.0,0.0	0.5	0.2,0.8	0.596269	1.07	1.05,1.09	0
alprazolam	57100010	99	2941	7243.6	23.6	19.9,27.7	0.0	0.0,0.1	-0.8	-1.0,-0.5	0.493012	1.08	1.05,1.11	0
ranitidine	49200020	99	2434	7302.0	48.8	44.5,53.5	0.0	0.0,0.0	-0.4	-0.6,-0.1	0.411049	1.08	1.06,1.10	0
ondansetron	50250065	98	3175	5263.9	88.6	86.3,91.0	0.1	0.0,0.1	-0.5	-0.8,-0.1	0.340718	1.09	1.06,1.11	0
bystolic	33200040	16	705	2115.0	54.1	49.5,58.8	0.6	0.4,0.9	-1.1	-1.5,-0.8	0.498312	1.09	1.04,1.16	0

fluticasone-salmeterol	44209902	50	3245	5516.0	23.0	23.0,23.0	0.0	0.0,0.0	-0.1	-0.1,-0.1	0.859276	1.10	1.10,1.10	0
cyclobenzaprine	75100050	68	2694	4302.6	35.6	28.1,44.0	0.3	0.2,0.4	-0.3	-0.4,-0.2	0.48387	1.10	1.07,1.13	0
baclofen	75100010	74	1600	4800.0	42.0	37.4,46.6	0.1	0.0,0.2	-0.2	-0.4,0.0	0.383528	1.10	1.06,1.14	0
acetaminophen-codeine	65991002	99	1738	5214.0	43.1	38.0,48.7	0.1	0.1,0.2	-0.5	-0.7,-0.1	0.403562	1.10	1.07,1.13	0
paroxetine	58160060	87	1172	3516.0	35.6	30.7,41.1	0.1	0.0,0.2	-1.2	-1.6,-0.8	0.266156	1.10	1.05,1.15	0
nystatin	90150080	89	1587	4761.0	73.5	70.7,76.0	0.1	0.0,0.2	-1.2	-1.4,-1.0	0.257876	1.10	1.08,1.12	0
fluoxetine	58160040	96	2158	6474.0	40.3	35.2,45.4	0.1	0.0,0.1	-0.7	-1.1,-0.3	0.215723	1.11	1.06,1.15	0
simvastatin	39400075	100	3596	10788.0	81.5	76.1,87.0	0.0	0.0,0.0	-0.9	-1.2,-0.5	0.0966	1.11	1.09,1.13	0
temazepam	60201030	82	1236	3580.6	39.5	36.1,43.3	0.1	0.1,0.2	-0.8	-1.0,-0.7	0.350397	1.11	1.09,1.13	0
rivastigmine	62051040	49	2588	7764.0	14.2	12.4,17.3	0.5	0.4,0.7	-3.6	-4.1,-3.3	0.023053	1.12	1.10,1.15	0
methylphenidate	61400020	51	1171	2876.7	33.2	32.0,35.6	0.0	0.0,0.0	-0.5	-0.5,-0.4	0.477513	1.12	1.11,1.13	0
solifenacin	54100055	83	1404	4211.4	21.2	17.0,26.0	0.2	0.1,0.3	-0.8	-1.1,-0.6	0.587909	1.12	1.09,1.15	0
famotidine	49200030	97	1859	5577.0	36.3	32.5,39.9	0.0	0.0,0.1	-1.7	-2.0,-1.4	0.087414	1.17	1.14,1.19	0
tolterodine	54100060	72	901	2703.0	24.3	20.2,28.3	0.2	0.1,0.2	-1.6	-1.8,-1.4	0.347666	1.19	1.16,1.23	0
buspirone	57200005	53	1723	5169.0	54.2	52.6,55.8	0.0	0.0,0.1	-2.4	-2.6,-2.3	0.030615	1.20	1.17,1.22	0
nitrofurantoin	16800050	52	2572	6567.9	33.3	33.0,33.9	1.0	1.0,1.0	-1.1	-1.1,-1.0	0.160761	1.20	1.20,1.20	0
mirtazapine	58030050	99	3686	11056.3	50.2	48.6,51.7	0.1	0.0,0.1	-2.5	-2.8,-2.0	0.170481	1.30	1.25,1.35	0
citalopram	58160020	100	4298	12894.0	73.9	70.2,77.3	0.0	0.0,0.0	-2.2	-2.6,-1.8	0.1808	1.32	1.28,1.37	0
sertraline	58160070	100	5524	16564.6	17.6	15.5,19.7	0.0	0.0,0.0	-2.7	-3.1,-2.3	0.013764	1.35	1.31,1.40	0

lorazepam	57100060	100	3077	7649.0	34.3	29.2,39.5	0.0	0.0,0.0	-2.5	-2.9,-2.1	0.004541	1.37	1.33,1.40	0
escitalopram	58160034	100	5041	15123.0	13.5	11.0,15.8	0.0	0.0,0.0	-4.1	-4.4,-3.8	0.002224	1.47	1.42,1.52	0
premarin	55350025	48	613	1232.0	31.0	31.0,31.0	1.0	1.0,1.0	-2.7	-2.7,-2.6	0.053284	1.56	1.56,1.57	0
quetiapine	59153070	94	2934	6345.1	38.6	34.0,43.5	0.1	0.1,0.2	-5.2	-5.9,-4.6	0.011269	1.60	1.51,1.70	0
memantine	62053550	100	6282	18151.7	57.3	47.0,66.8	1.0	0.9,1.0	-9.8	-11.3,-8.2	0.023701	2.15	1.95,2.36	0
donepezil	62051025	100	15503	26179.9	98.5	91.8,104.7	0.5	0.4,0.6	-11.2	-13.1,-9.2	0.028544	2.94	2.57,3.31	0

1. adjusted hazard ratio and 5-year standardized AD-free survival differences and hazard ratios after inverse probability of treatment re-weighting (IPTW) by regularized logistic regression-based PS model (LR-PS) using our proposed model selection strategy, adjusted for 267 covariates in total: age, sex, comorbidities, medication history, and the time from MCI initiation date to the trial drug initiation date. Covariates were collected during baseline period.
2. Drugs were ranked by the estimated aHR.
3. We selected drugs with at least 10% emulated trials were balanced and for each balanced trial all the unbalanced features were balanced after IPTW.
4. Control groups are constructed randomly, either from alternative drug cohorts or similar drug cohorts under ATC L2. We set number of patients in the control group to maximum 3-folds as the treated group and we report the mean number of all balanced trials here.
5. All statistics were sample means over balanced trials. Bootstrapped p-values for one-sample T-test and 1,000 bootstrapped 95% confidence interval were reported here.

Supplementary Table S7-Simulation study results summary

	Val-AUC Select					Val-Loss Select					Our strategy				
**Exp setup**	**HR estimate mean**	**HR estimate std**	**HR estimate bias**	**HR estimate mse**	**CI Coverage (%)**	**HR estimate mean**	**HR estimate std**	**HR estimate bias**	**HR estimate mse**	**CI Coverage (%)**	**HR estimate mean**	**HR estimate std**	**HR estimate bias**	**HR estimate mse**	**CI Coverage (%)**
**5000-lin-incorrect**	0.693	0.020	0.115	0.0136	0.0%	0.657	0.026	0.079	0.0070	14.0%	0.611	0.023	**0.034**	**0.0016**	**71.0%**
**5000-lin-correct**	0.694	0.021	0.116	0.0138	0.0%	0.657	0.027	0.079	0.0070	16.0%	0.611	0.026	**0.035**	**0.0018**	**73.0%**
**5000-nonLin-incorrect**	0.675	0.022	0.097	0.0099	0.0%	0.661	0.025	0.083	0.0076	8.0%	0.631	0.034	**0.053**	**0.0040**	**66.0%**
**5000-nonLin-correct**	0.717	0.030	0.139	0.0201	0.0%	0.653	0.027	0.075	0.0063	20.0%	0.601	0.023	**0.026**	**0.0011**	**81.0%**
**4500-lin-incorrect**	0.698	0.020	0.120	0.0148	0.0%	0.664	0.029	0.086	0.0081	18.0%	0.613	0.029	**0.036**	**0.0020**	**81.0%**
**4500-lin-correct**	0.698	0.022	0.120	0.0149	0.0%	0.662	0.031	0.084	0.0080	21.0%	0.610	0.029	**0.035**	**0.0019**	**84.0%**
**4500-nonLin-incorrect**	0.680	0.022	0.102	0.0108	0.0%	0.668	0.024	0.090	0.0086	6.0%	0.642	0.035	**0.064**	**0.0053**	**52.0%**
**4500-nonLin-correct**	0.723	0.029	0.145	0.0219	0.0%	0.661	0.026	0.083	0.0076	11.0%	0.603	0.025	**0.028**	**0.0012**	**85.0%**
**4000-lin-incorrect**	0.705	0.023	0.127	0.0166	0.0%	0.673	0.029	0.095	0.0098	6.0%	0.613	0.026	**0.036**	**0.0019**	**67.0%**
**4000-lin-correct**	0.705	0.025	0.127	0.0167	0.0%	0.673	0.031	0.095	0.0099	11.0%	0.613	0.028	**0.036**	**0.0020**	**74.0%**
**4000-nonLin-incorrect**	0.688	0.025	0.110	0.0126	0.0%	0.675	0.030	0.097	0.0103	10.0%	0.654	0.039	**0.077**	**0.0073**	**50.0%**
**4000-nonLin-correct**	0.734	0.033	0.156	0.0255	0.0%	0.672	0.030	0.094	0.0096	11.0%	0.607	0.026	**0.032**	**0.0015**	**78.0%**
**3500-lin-incorrect**	0.709	0.025	0.131	0.0177	0.0%	0.684	0.033	0.106	0.0124	9.0%	0.608	0.034	**0.035**	**0.0021**	**87.0%**
**3500-lin-correct**	0.708	0.026	0.130	0.0176	0.0%	0.681	0.032	0.103	0.0117	7.0%	0.608	0.035	**0.036**	**0.0021**	**86.0%**
**3500-nonLin-incorrect**	0.692	0.025	0.113	0.0135	0.0%	0.678	0.028	0.100	0.0108	6.0%	0.662	0.039	**0.084**	**0.0085**	**38.0%**
**3500-nonLin-**	0.739	0.033	0.161	0.027	0.0%	0.674	0.034	0.096	0.010	22.0%	0.600	0.029	**0.029**	**0.001**	**88.0%**

**correct**				0					3					**3**	
**3000-lin-incorrect**	0.715	0.029	0.137	0.0196	1.0%	0.692	0.033	0.114	0.0141	3.0%	0.606	0.034	**0.033**	**0.0019**	**87.0%**
**3000-lin-correct**	0.716	0.030	0.138	0.0198	1.0%	0.692	0.034	0.114	0.0142	6.0%	0.606	0.035	**0.034**	**0.0020**	**87.0%**
**3000-nonLin-incorrect**	0.699	0.031	0.120	0.0155	1.0%	0.685	0.029	0.107	0.0124	6.0%	0.654	0.046	**0.076**	**0.0078**	**55.0%**
**3000-nonLin-correct**	0.745	0.031	0.166	0.0287	0.0%	0.685	0.035	0.107	0.0126	16.0%	0.596	0.029	**0.027**	**0.0012**	**90.0%**

Ground truth marginal hazard ratio: 0.578

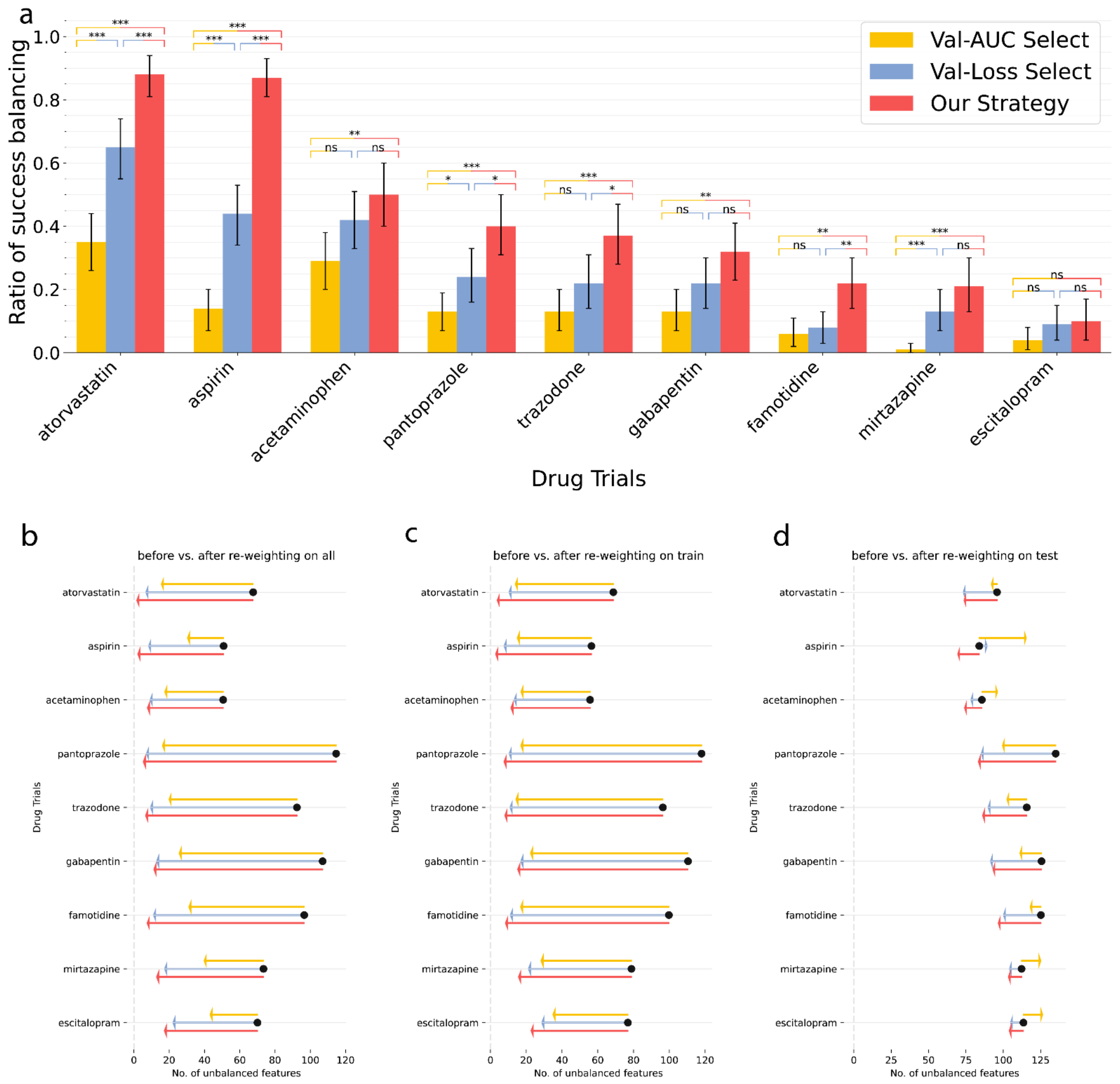

Supplementary Fig. S1. Balance performance of Gradient Boosting Machine-based Propensity Score models (GBM-PS) selected by different model selection strategies, OneFlorida database, 2012-2020. (a) The proportion of successfully balanced drug trials by GBM-PS selected by different model selection strategies. (b-d) The average number of unbalanced baseline covariates before and after re-weighting on (b) training and testing combined set, (c) training set, and (d) unseen testing set. Three model selection strategies are (i) the AUC score on the validation fold during the cross-validation procedure, (ii) the cross-entropy loss on the validation fold, and (iii) our proposed strategy, which leverages balance performance on the training and validation combined folds and generalization performance on the validation fold. We reported drugs with ≥ 10% balanced trials. A covariate is assumed balanced if its standardized mean difference (SMD) of its prevalence between exposure groups is at most 0.1 and a trial is assumed balanced if the ratio of unbalanced features among all covariates before/after IPTW ≤ 2%. The error bars indicate 95% confidence intervals by 1000-times bootstrapping. Welch's t-test (two-sample, two-sided) is used for testing the means of binary indicators for balanced trials, and *, $p < 0.05$; **, $p < 0.01$; ***, $p < 0.001$; not significant (ns), $p \geq 0.05$; AUC, area under the receiver operating characteristic curve. Source data are provided as a Source Data file.

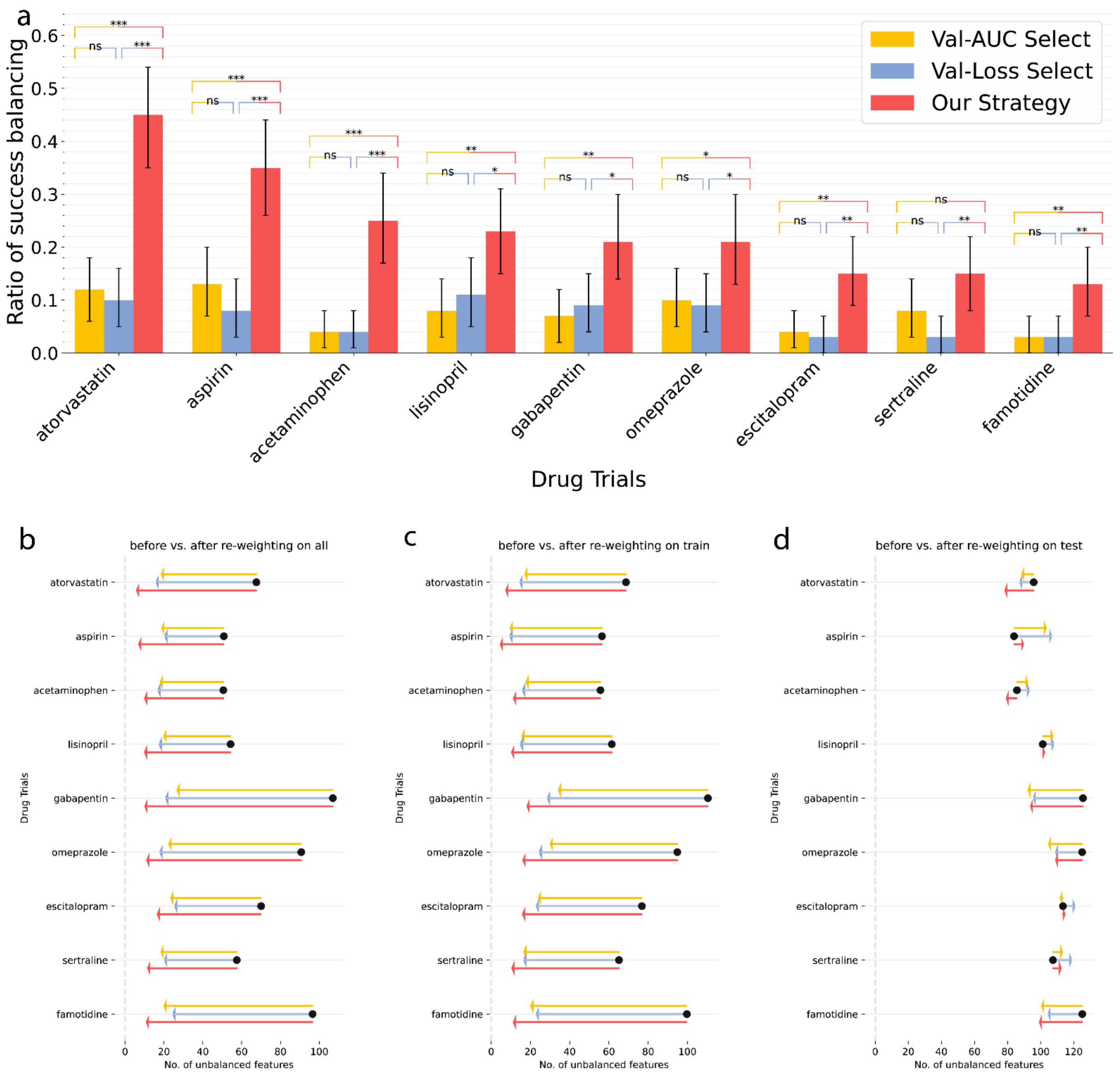

Supplementary Fig. S2. Balance performance of Multi-Layer Lerceptron-based Propensity Score models (MLP-PS) selected by different model selection strategies, OneFlorida database, 2012-2020. (a) The proportion of successfully balanced drug trials by MLP-PS selected by different model selection strategies. (b-d) The average number of unbalanced baseline covariates before and after re-weighting on (b) training and testing combined set, (c) training set, and (d) unseen testing set. Three model selection strategies are (i) the AUC score on the validation fold during the cross-validation procedure, (ii) the cross-entropy loss on the validation fold, and (iii) our proposed strategy, which leverages balance performance on the training and validation combined folds and generalization performance on the validation fold. We reported drugs with ≥ 10% balanced trials. A covariate is assumed balanced if its standardized mean difference (SMD) of its prevalence between exposure groups is at most 0.1 and a trial is assumed balanced if the ratio of unbalanced features among all covariates before/after IPTW ≤ 2%. The error bars indicate 95% confidence intervals by 1000-times bootstrapping. Welch's t-test (two-sample, two-sided) is used for testing the means of binary indicators for balanced trials, and *, $p < 0.05$; **, $p < 0.01$; ***, $p < 0.001$; not significant (ns), $p \geq 0.05$; AUC, area under the receiver operating characteristic curve. Source data are provided as a Source Data file.

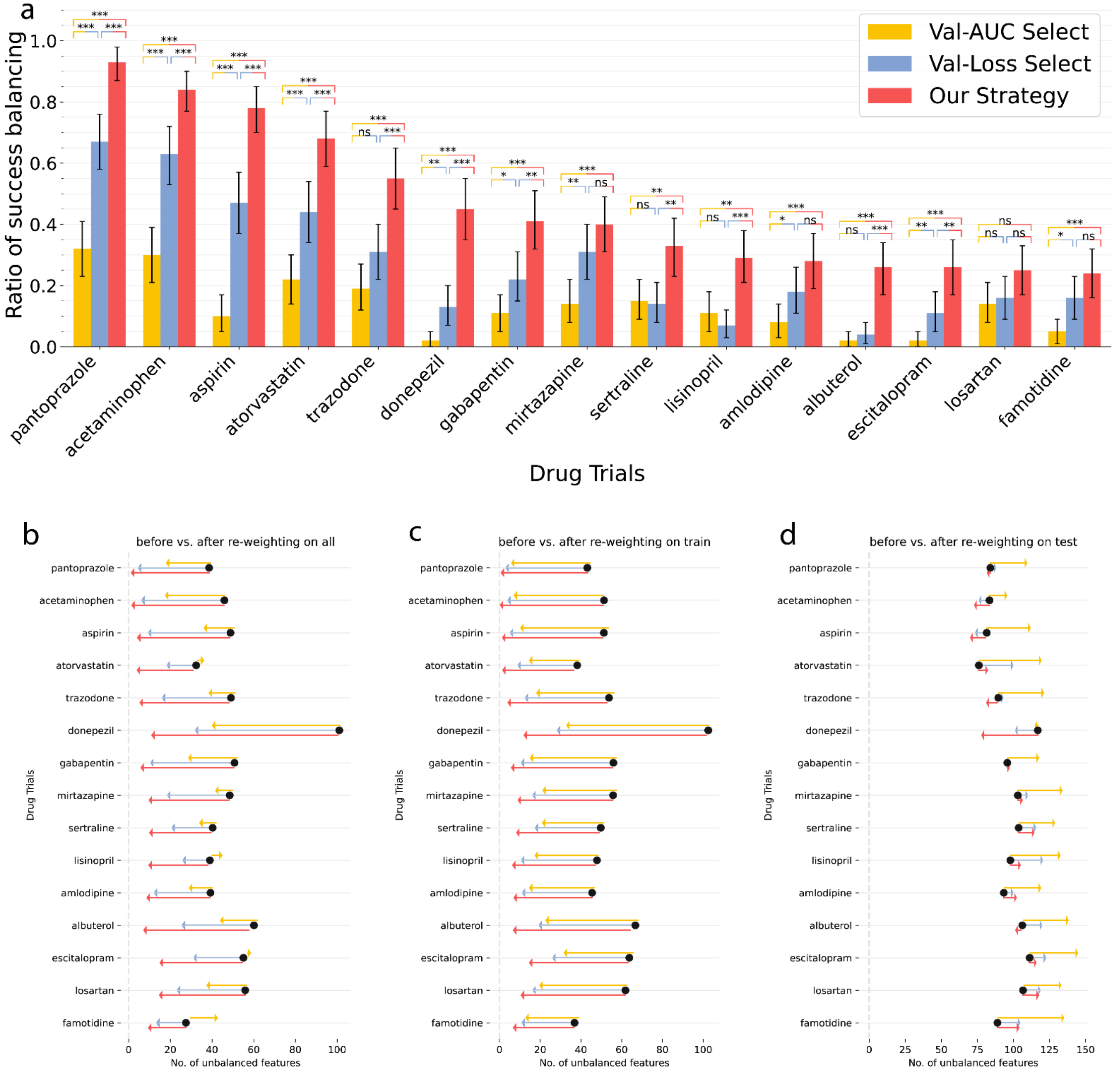

Supplementary Fig. S3. Balance performance of Long Short Term Memory neural networks-based Propensity Score models (LSTM-PS) selected by different model selection strategies, OneFlorida database, 2012-2020. (a) The proportion of successfully balanced drug trials by LSTM-PS selected by different model selection strategies. (b-d) The average number of unbalanced baseline covariates before and after re-weighting on (b) training and testing combined set, (c) training set, and (d) unseen testing set. Three model selection strategies are (i) the AUC score on the validation fold during the cross-validation procedure, (ii) the cross-entropy loss on the validation fold, and (iii) our proposed strategy, which leverages balance performance on the training and validation combined folds and generalization performance on the validation fold. We reported drugs with ≥ 10% balanced trials. A covariate is assumed balanced if its standardized mean difference (SMD) of its prevalence between exposure groups is at most 0.1 and a trial is assumed balanced if the ratio of unbalanced features among all covariates before/after IPTW ≤ 2%. The error bars indicate 95% confidence intervals by 1000-times bootstrapping. Welch's t-test (two-sample, two-sided) is used for testing the means of binary indicators for balanced trials, and *, $p < 0.05$; **, $p < 0.01$; ***, $p < 0.001$; not significant (ns), $p \geq 0.05$; AUC, area under the receiver operating characteristic curve. Source data are provided as a Source Data file.

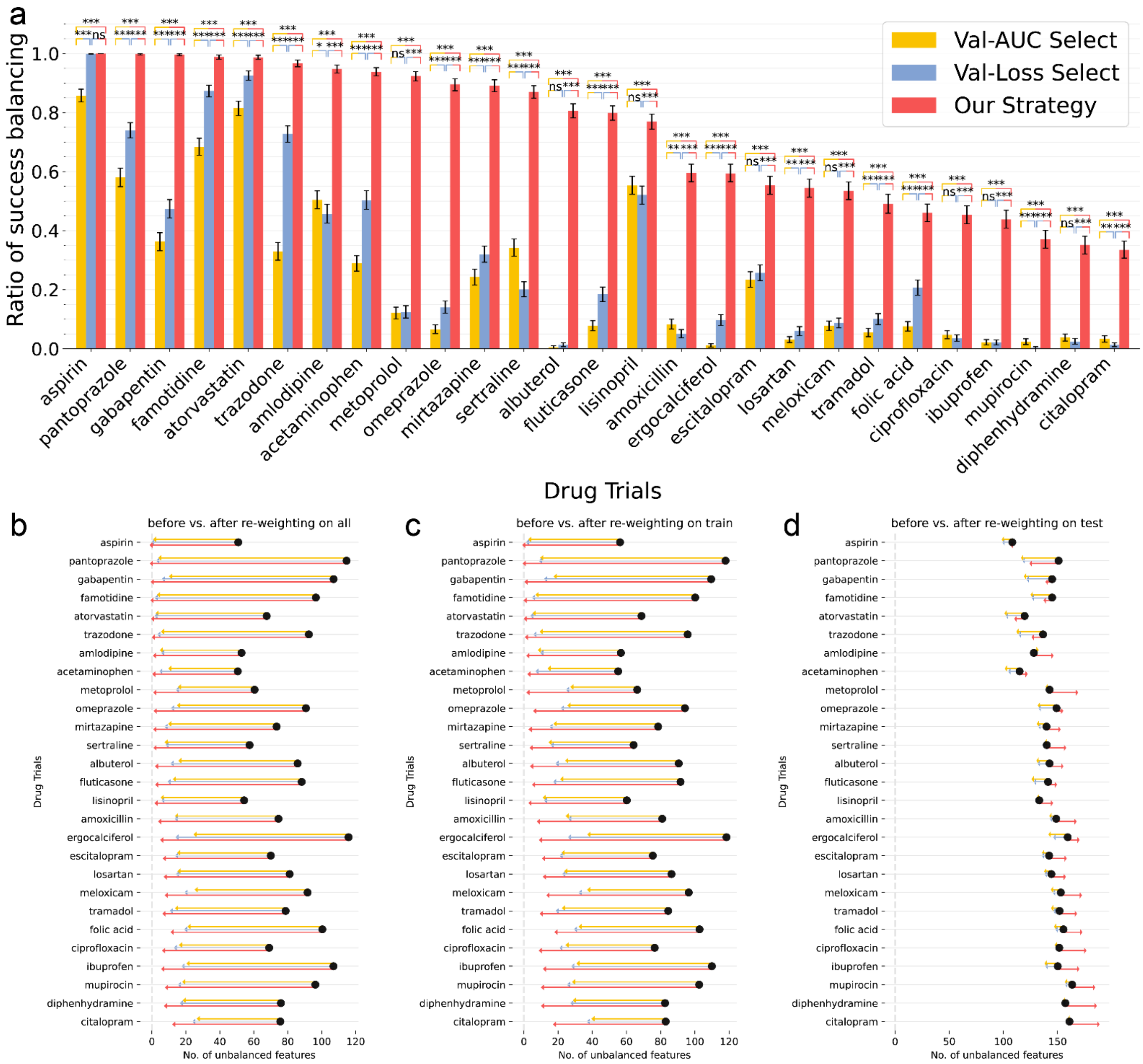

Supplementary Fig. S4. Balance performance of the regularized Logistic Regression-based Propensity Score (LR-PS) models selected by different model selection strategies under the Nested Cross-Validation framework, OneFlorida, 2012-2020. (a) The proportion of successfully balanced drug trials by LR-PS selected by different model selection strategies. (b-d) The average number of unbalanced baseline covariates before and after re-weighting on (b) training and testing combined set, (c) training set, and (d) unseen testing set. Three model selection strategies are (i) the AUC score on the validation fold during the cross-validation procedure, (ii) the cross-entropy loss on the validation fold, and (iii) our proposed strategy, which leverages balance performance on the training and validation combined folds and generalization performance on the validation fold. We used 10-fold outer cross-validation and 5-fold inner cross-validation. We reported drugs with ≥ 10% balanced trials. A covariate is assumed balanced if its standardized mean difference (SMD) of its prevalence between exposure groups is at most 0.1 and a trial is assumed balanced if the ratio of unbalanced features among all covariates before/after IPTW ≤ 2%. The error bars indicate 95% confidence intervals by 1000-times bootstrapping. Welch's t-test (two-sample, two-sided) is used for testing the means of binary indicators for balanced trials, and *, $p < 0.05$; **, $p < 0.01$; ***, $p < 0.001$; not significant (ns), $p \geq 0.05$; AUC, area under the receiver operating characteristic curve. Source Data are provided as a Source Data file.

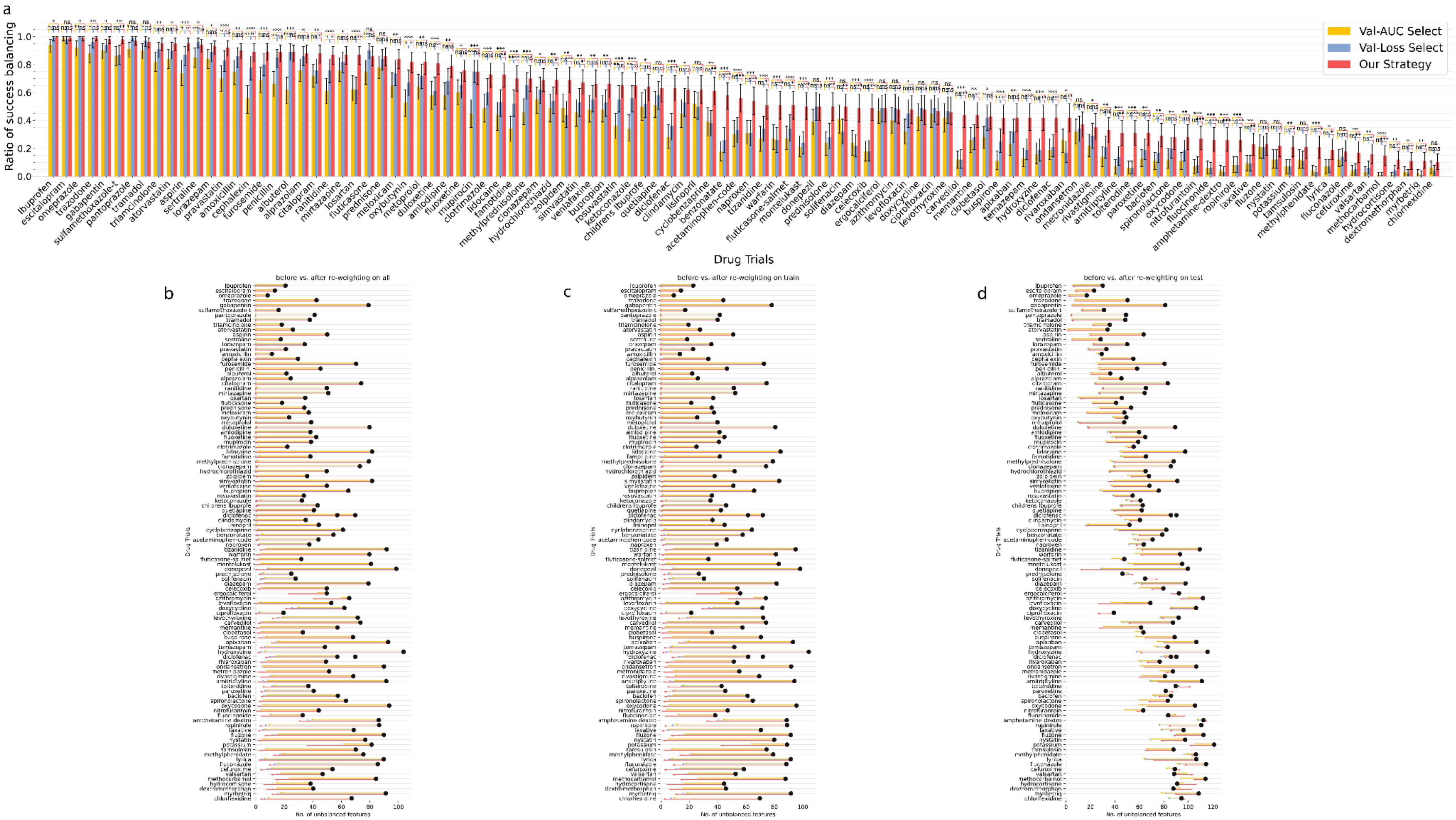
a
Ratio of success balancing
Val-AUC Select
Val-Loss Select
Our Strategy
Drug Trials
b
before vs. after re-weighting on all
No. of unbalanced features
c
before vs. after re-weighting on train
No. of unbalanced features
d
before vs. after re-weighting on test
No. of unbalanced features
Drug Trials

Supplementary Fig. S5. Balance performance of the regularized Logistic Regression-based Propensity Score models (LR-PS) selected by different model selection strategies, MarketScan database, 2009-2020. (a) The proportion of successfully balanced drug trials by LR-PS selected by different model selection strategies. (b-d) The average number of unbalanced baseline covariates before and after re-weighting on (b) training and testing combined set, (c) training set, and (d) unseen testing set. Three model selection strategies are (i) the AUC score on the validation fold during the cross-validation procedure, (ii) the cross-entropy loss on the validation fold, and (iii) our proposed strategy, which leverages balance performance on the training and validation combined folds and generalization performance on the validation fold. We reported drugs with ≥ 10% balanced trials. A covariate is assumed balanced if its standardized mean difference (SMD) of its prevalence between exposure groups is at most 0.1 and a trial is assumed balanced if the ratio of unbalanced features among all covariates before/after IPTW ≤ 2%. The error bars indicate 95% confidence intervals by 1000-times bootstrapping. Welch's t-test (two-sample, two-sided) is used for testing the means of binary indicators for balanced trials, and *, $p < 0.05$; **, $p < 0.01$; ***, $p < 0.001$; not significant (ns), $p \geq 0.05$; AUC, area under the receiver operating characteristic curve. Source data are provided as a Source Data file.

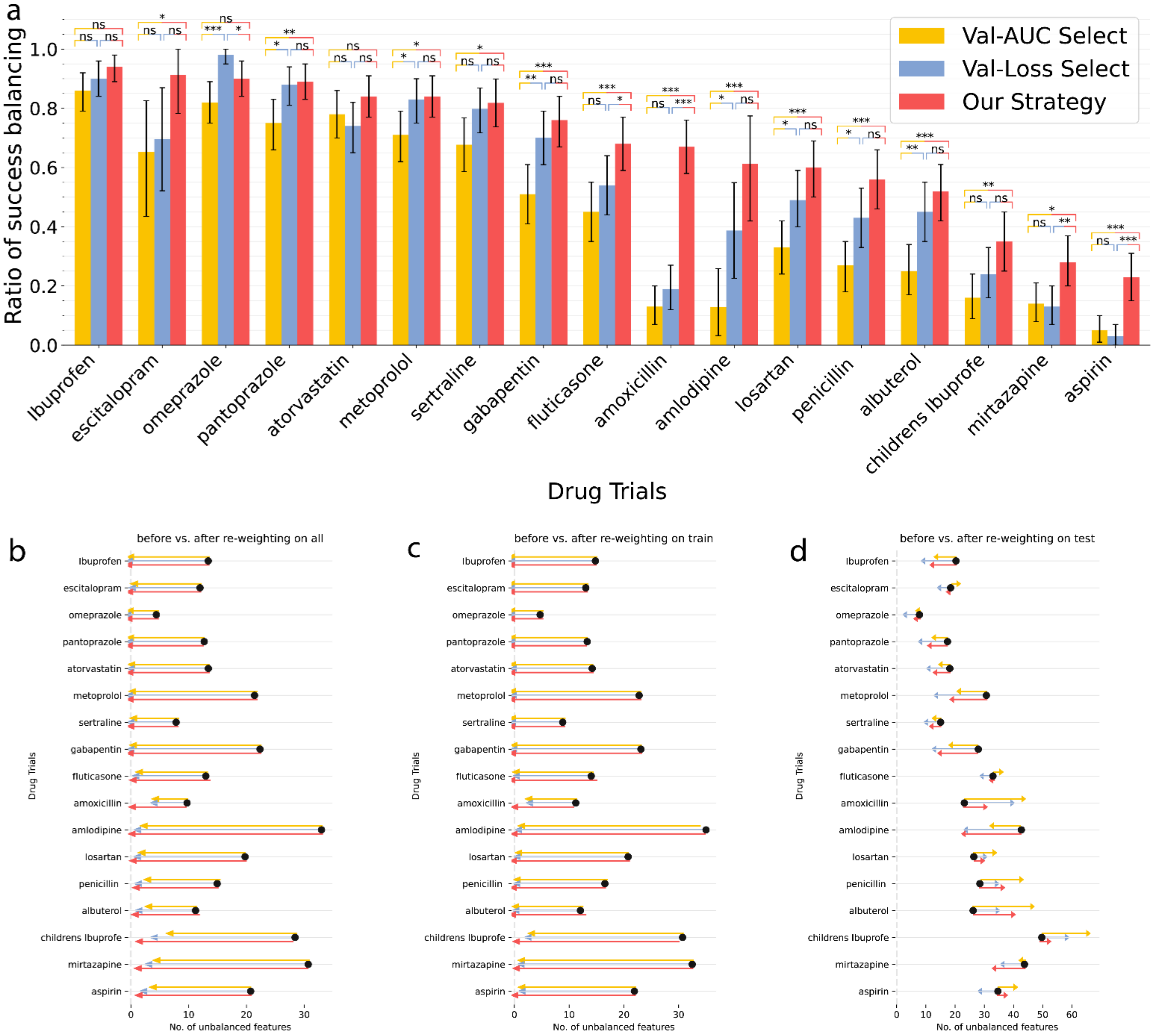

Supplementary Fig. S6. Balance performance of Long Short Term Memory neural networks-based Propensity Score models (LSTM-PS) selected by different model selection strategies, MarketScan database, 2009-2020. (a) The proportion of successfully balanced drug trials by LSTM-PS selected by different model selection strategies. (b-d) The average number of unbalanced baseline covariates before and after re-weighting on (b) training and testing combined set, (c) training set, and (d) unseen testing set. Three model selection strategies are (i) the AUC score on the validation fold during the cross-validation procedure, (ii) the cross-entropy loss on the validation fold, and (iii) our proposed strategy, which leverages balance performance on the training and validation combined folds and generalization performance on the validation fold. We reported drugs with ≥ 10% balanced trials. A covariate is assumed balanced if its standardized mean difference (SMD) of its prevalence between exposure groups is at most 0.1 and a trial is assumed balanced if the ratio of unbalanced features among all covariates before/after IPTW ≤ 2%. The error bars indicate 95% confidence intervals by 1000-times bootstrapping. Welch's t-test (two-sample, two-sided) is used for testing the means of binary indicators for balanced trials, and *, $p < 0.05$; **, $p < 0.01$; ***, $p < 0.001$; not significant (ns), $p \geq 0.05$; AUC, area under the receiver operating characteristic curve. Source data are provided as a Source Data file.

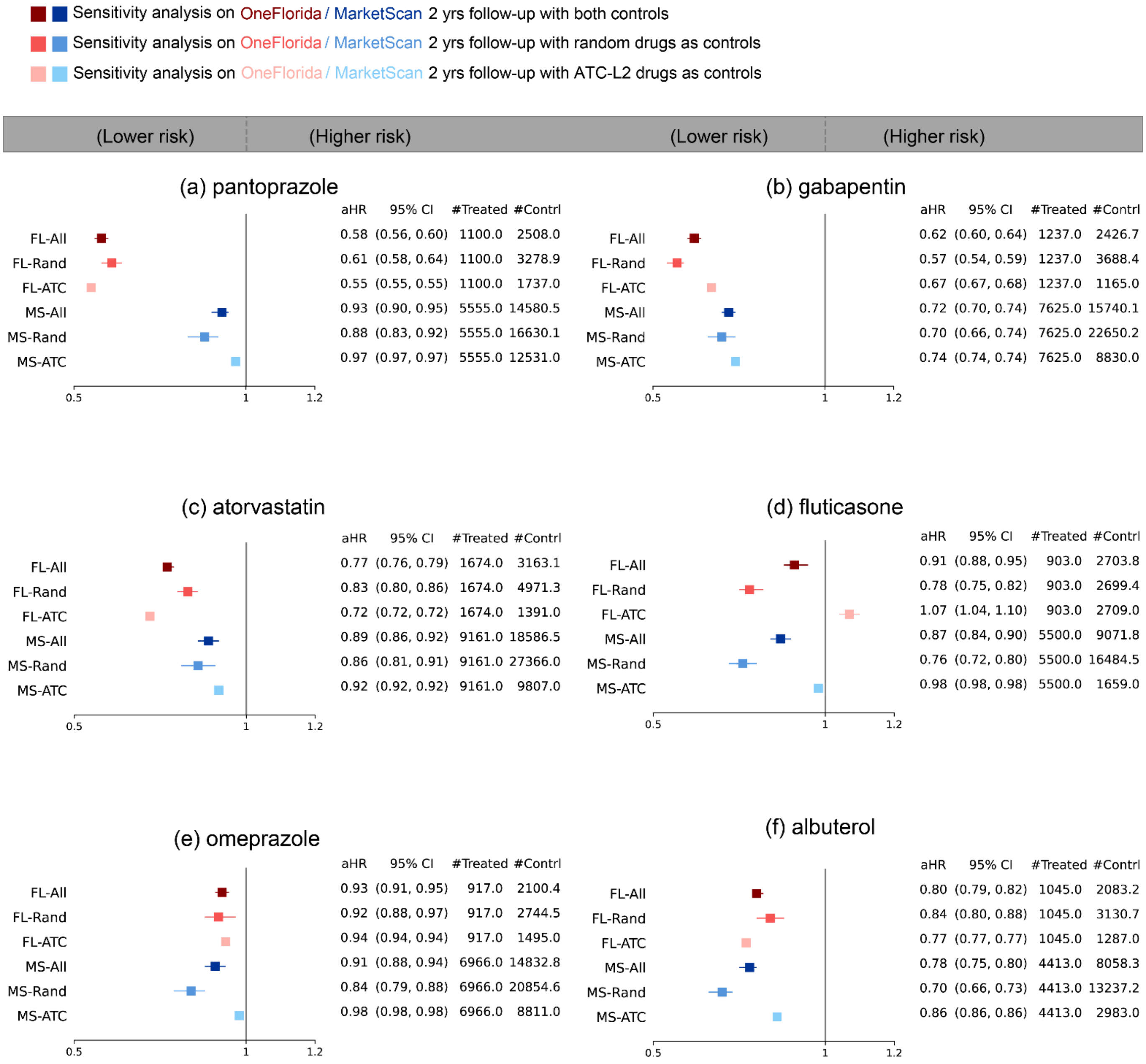

Supplementary Fig. S7. Generated drug repurposing hypotheses for AD with adjusted hazard ratios within the two-year follow-up period. Trial emulations of these drugs (a-f) were performed on OneFlorida (FL) and MarketScan (MS) data separately. For each drug, treated groups consisted of patients who were exposed to the trial drug (eligibility criterion in the Methods section), and control groups were built by either: (i) randomly selecting alternative drug groups, or (ii) using drug groups under the same second-level Anatomical Therapeutic Chemical classification codes (ATC-L2) as the trial drug. The primary analysis emulated 100 trials consisting of 50 random control groups and 50 ATC-L2 control groups (FL-All and MS-All), and two sensitivity analyses using only random controls (FL-Rand and MS-Rand) or only ATC-L2 controls (FL-ATC and MS-ATC). Source data are provided as a Source Data file.

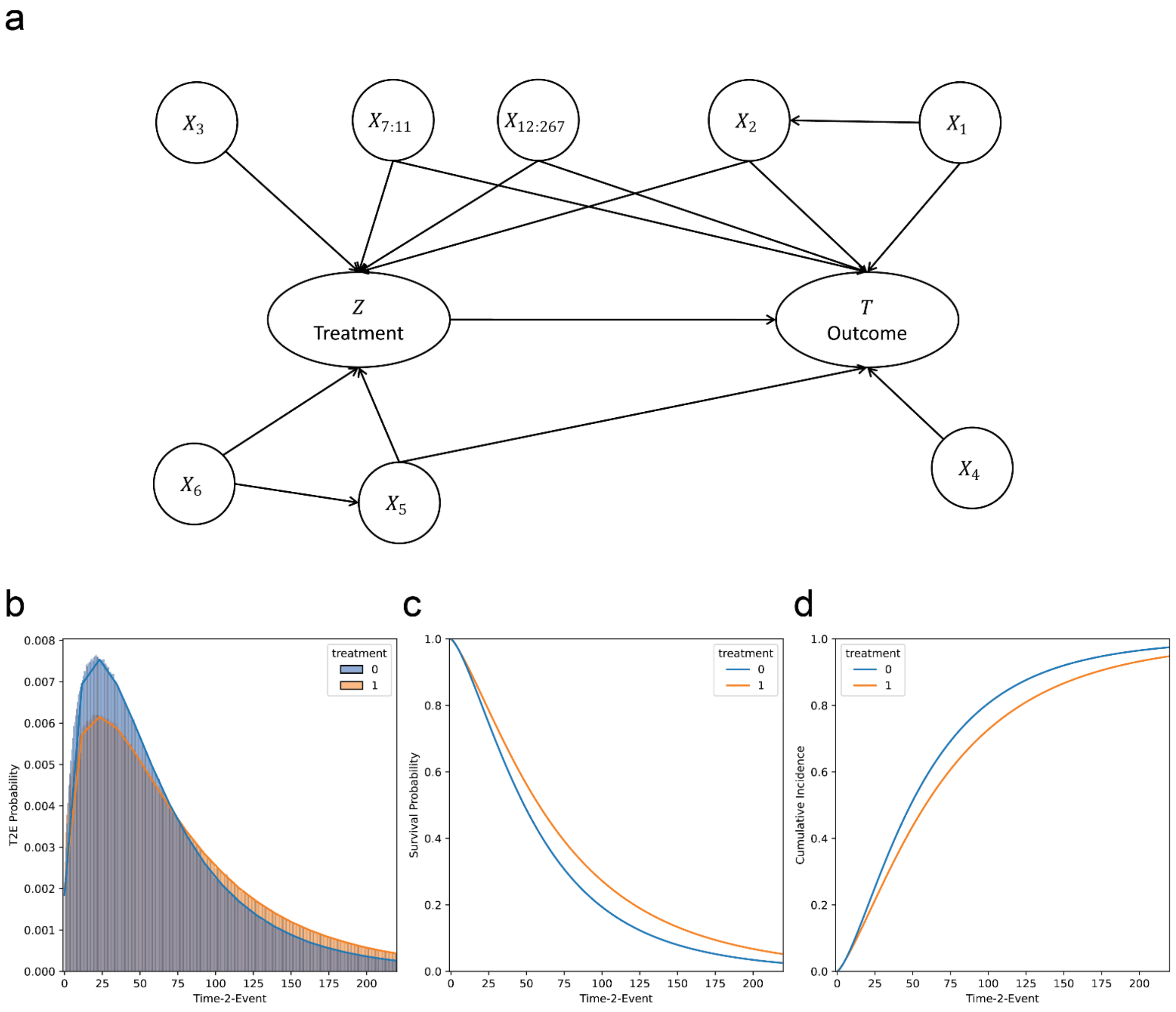

Supplementary Fig. S8. Data generation diagram and generated time-to-event samples in the simulation study. **(a)** the Directed Acyclic Graph of the hypothetical data generation process, **(b)** time-to-event distributions, **(c)** survival curves, and **(d)** cumulative incidence curves of the generated data from two exposure groups. The specifications of the data distributions and causal coefficients used in generating data samples were detailed in the method section.

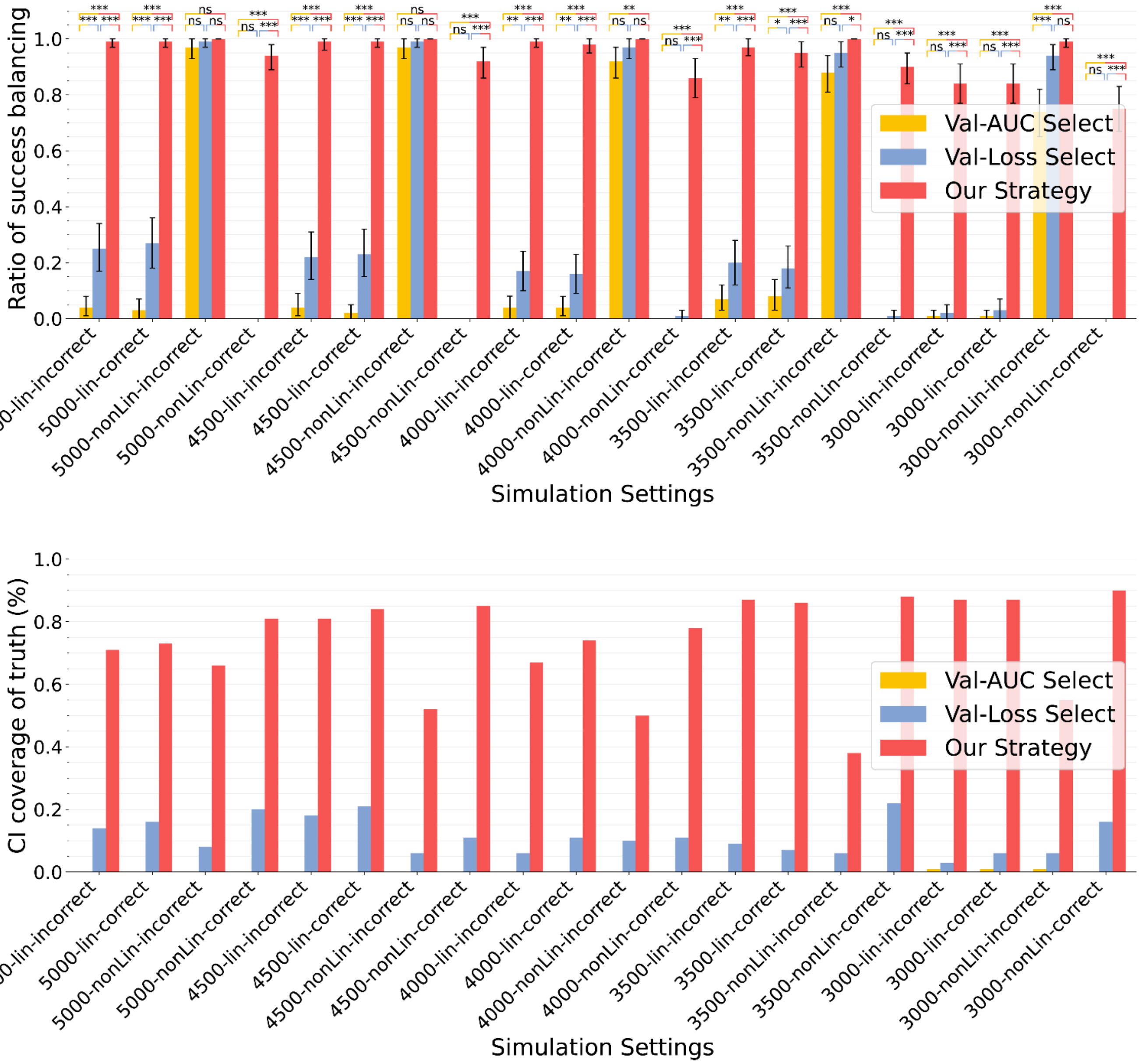

Supplementary Fig. S9. Balance performance and outcome estimates after IPTW in the simulation study, Part I. (a) The proportion of successfully balanced simulated trials under different settings; (b) the percentage of 95% confidence interval coverage of true hazard ratios. A covariate is assumed balanced if its standardized mean difference (SMD) of its prevalence between exposure groups is at most 0.1 and a trial is assumed balanced if the number of unbalanced features among all covariates before/after IPTW ≤ 2 in the simulation. Welch's t-test (two-sample, two-sided) is used for testing the means of binary indicators for balanced trials in (a). Source data are provided as a Source Data file.

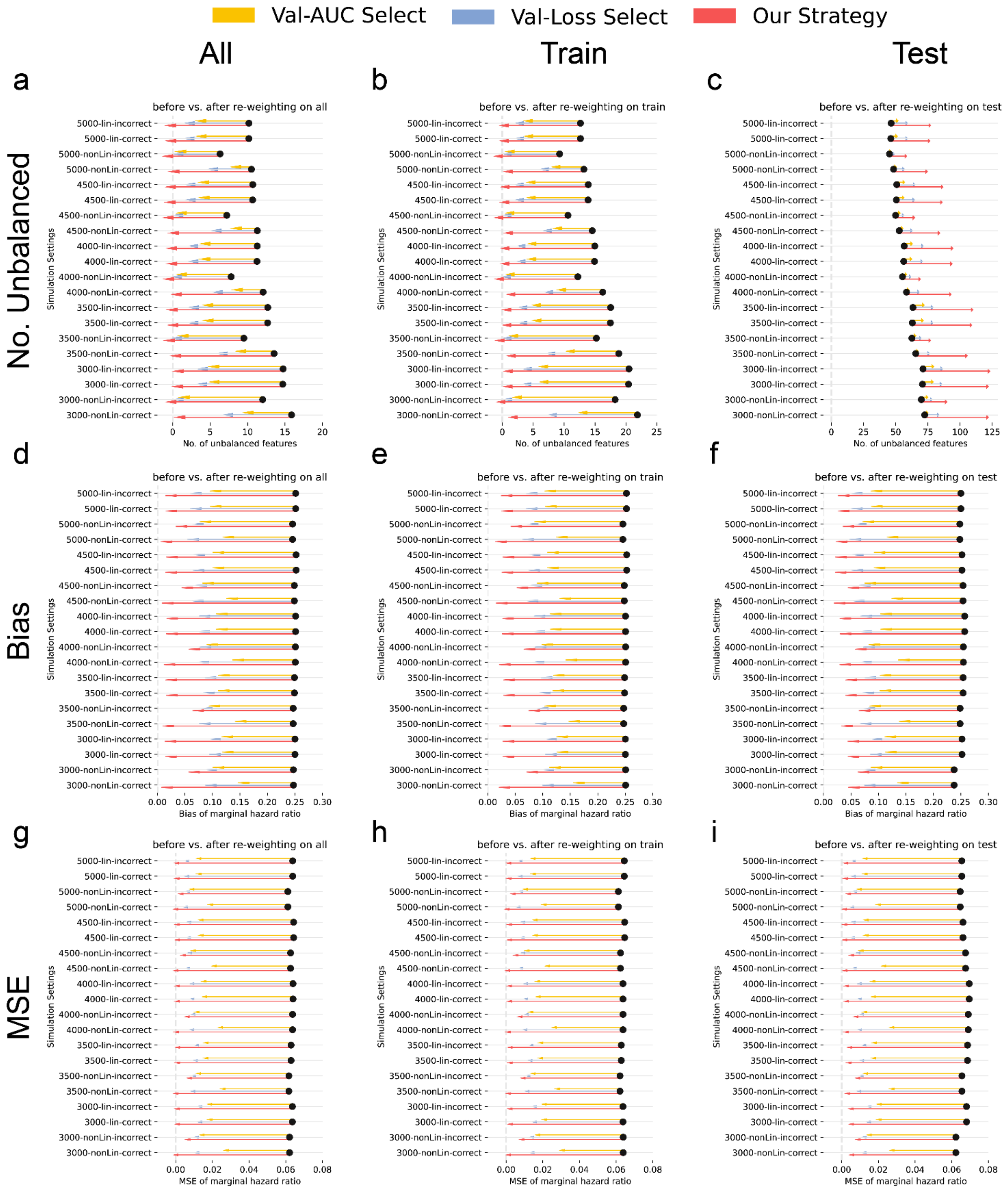

Supplementary Fig. S10. Balance performance and outcome estimates after IPTW in the simulation study, Part II. (a-c) The average number of unbalanced baseline covariates before and after re-weighting on (a) the all dataset, namely, the seen train and unseen test combined set, (b) the train set, and (c) the unseen test set. (d-f) The bias of estimates before and after re-weighting on (d) all datasets, (e) the train set, and (f) the unseen test set. (g-i) The mean squared error of estimates before and after re-weighting on the (g) all datasets, (h) the train set, and (i) the unseen test set. A covariate is assumed balanced if its standardized mean difference (SMD) of its prevalence between exposure groups is at most 0.1. Source data are provided as a Source Data file.

Supplementary Method – Causal Discovery of DAGs for emulated drug trials

Experiment setup

In addition to adjusting for a high-dimensional list of covariates, following another line of argument, we consider the hypothetical causal diagrams to adjust for good controls only. The hypothetical causal diagrams were built from both existing knowledge and data-driven causal discovery algorithms. Specifically, based on the best available knowledge, we first selected a subset of variables that are risk factors for or associated with AD, including age (the single most significant factor), gender, hypertension, hyperlipidemia, obesity, diabetes, heart failure, stroke, ischemic heart disease, traumatic brain injury due to brain damage, anxiety disorders, sleep disorders, alcohol use disorders, menopause, and periodontitis. Then we applied a constraint-based causal structure learning algorithm stable PC-algorithm (implemented by gcastle 1.0.3) to each emulated trial to learn its associated underlying directed acyclic graph (DAG). For each emulated trial, we excluded detected colliders (including M-colliders) and mediators and assumed that the remaining covariates are more likely to be confounders of the treatment assignment and the AD onset to adjust for.

We incorporated the following prior knowledge into the stable PC-algorithm:

- Both age and gender are likely to influence both the treatment assignment and the AD onset, thus we assumed there are direct edges from age or gender to both the treatment and outcome.
- All other covariates cannot influence either age or gender, thus no direct edges to age or gender.
- All the baseline covariates were measured before the treatment initiation, thus no direct edges from treatment to covariates before it.

We used the Bonferroni-method corrected significance level 2.9×10^{-4} (0.05/(k*(k-1))/2) and Fisher-zs test for the stable PC-algorithm, where k is the number of covariates. We also used a significance level of 0.05 as a sensitivity analysis.

Based on the inferred DAGs, we detected colliders, M-colliders, and mediators and further excluded these detected potentially bad controls from the adjustment. The following figures show exampled DAG for the emulated drug trials for pantoprazole, gabapentin, atorvastatin, fluticasone, omeprazole, and albuterol with random controls.

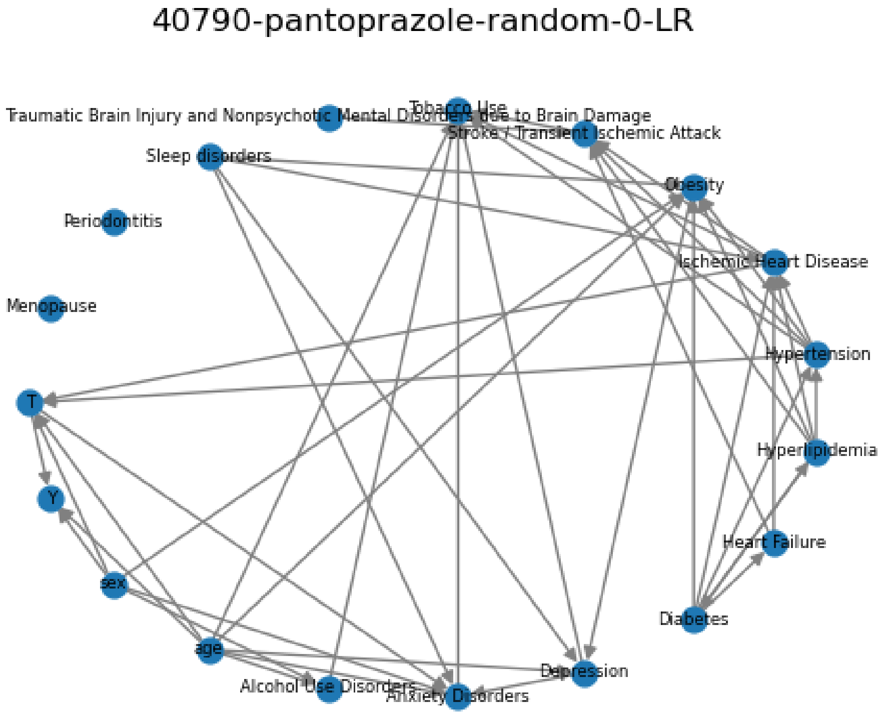

Sup. Method Fig. M1. One example of generated DAG from the pantoprazole trial from OneFlorida. The potentially bad controls are detected M-colliders including Anxiety Disorders and Tobacco Use.

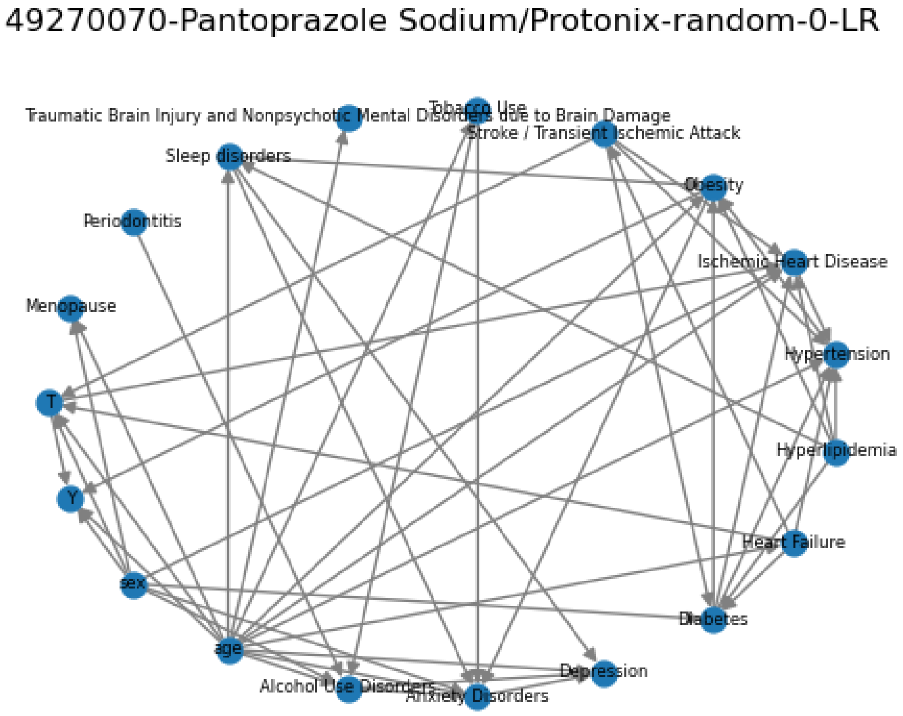

Sup. Method Fig. M2. One example of generated DAG from the pantoprazole trial from MarketScan. The potentially bad controls are detected mediators including Hypertension, Ischemic Heart Disease, Obesity, and M-Colliders including Anxiety Disorders and Tobacco Use.

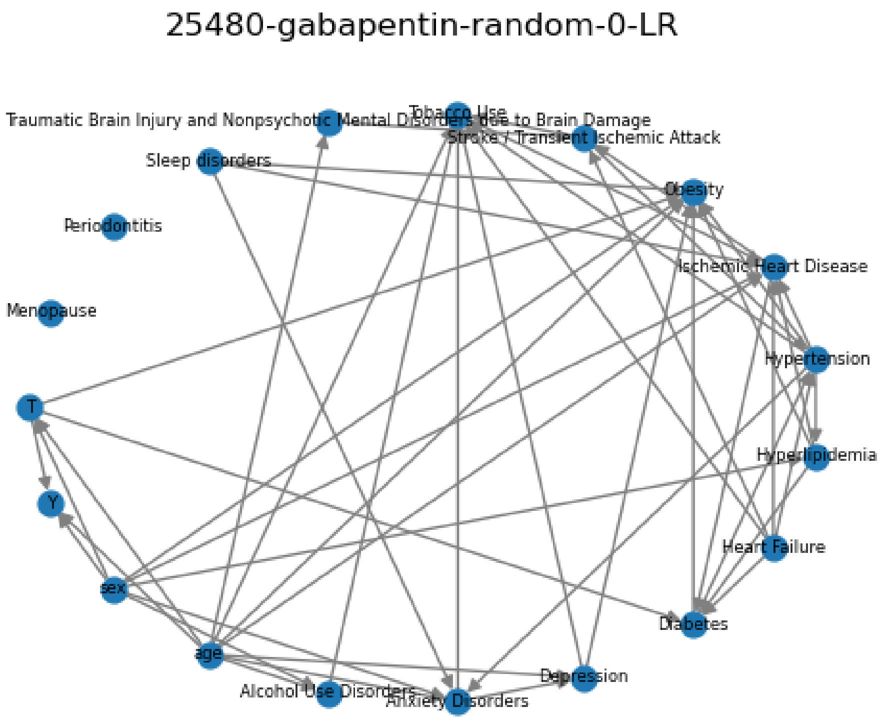

Sup. Method Fig. M3. One example of generated DAG from the gabapentin trial from OneFlorida. The potentially bad controls are detected M-colliders including Obesity, Hyperlipidemia, Ischemic Heart Disease, Diabetes, Tobacco Use, Hypertension, Depression, Stroke / Transient Ischemic Attack, Anxiety Disorders.

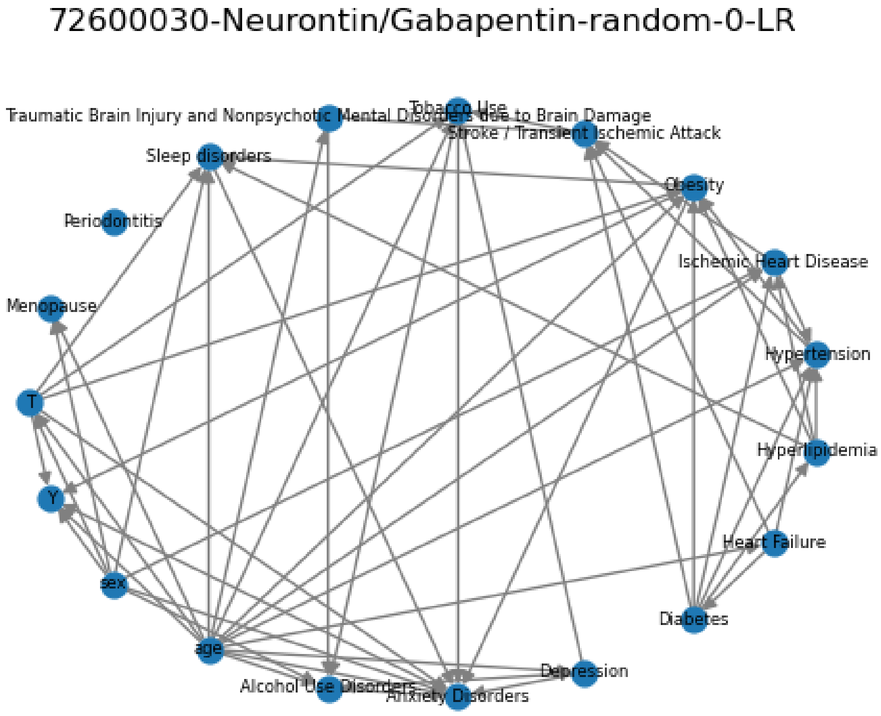

Sup. Method Fig. M4. One example of generated DAG from the gabapentin trial from MarketScan. The potentially bad controls are detected mediators including Anxiety Disorders, Alcohol Use Disorders, Sleep disorders, Obesity, Tobacco Use.

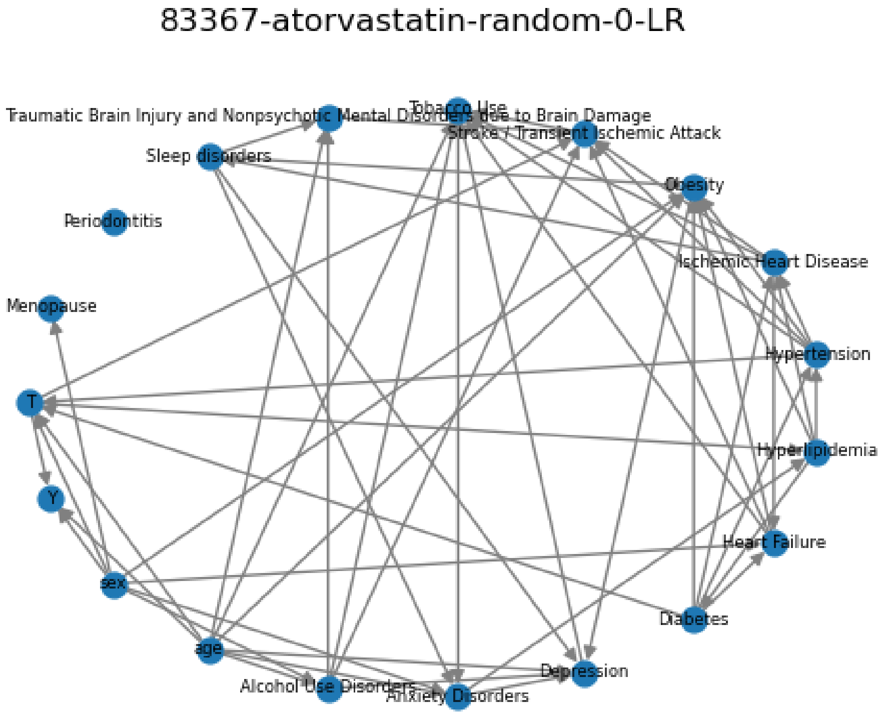

Sup. Method Fig. M5. One example of generated DAG from the atorvastatin trial from OneFlorida. No potentially bad controls are detected.

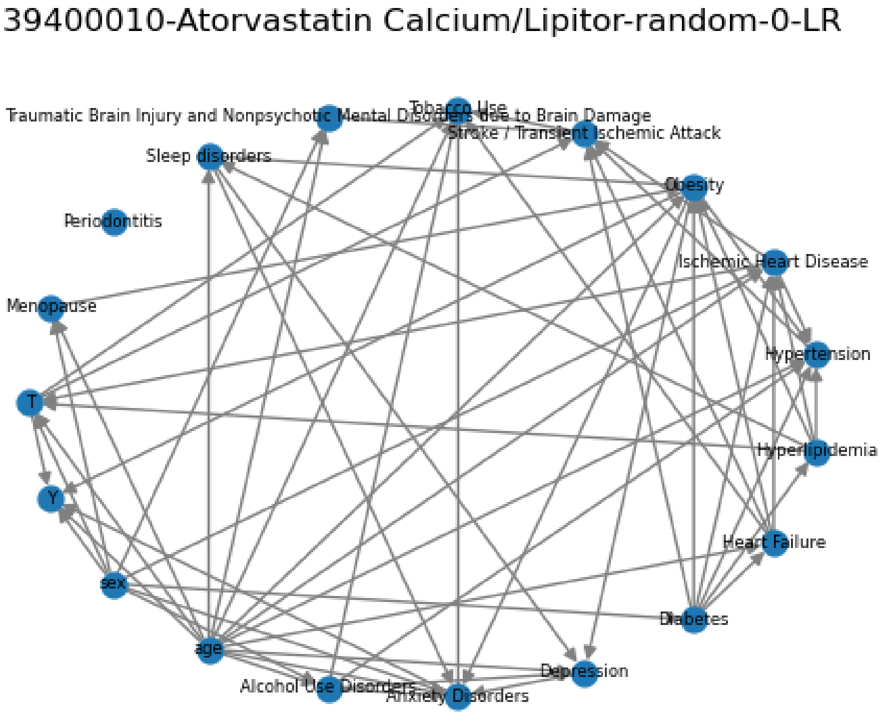

Sup. Method Fig. M6. One example of generated DAG from the atorvastatin trial from MarketScan. The potentially bad controls are detected M-colliders including Stroke / Transient Ischemic Attack, Hypertension, Tobacco Use.

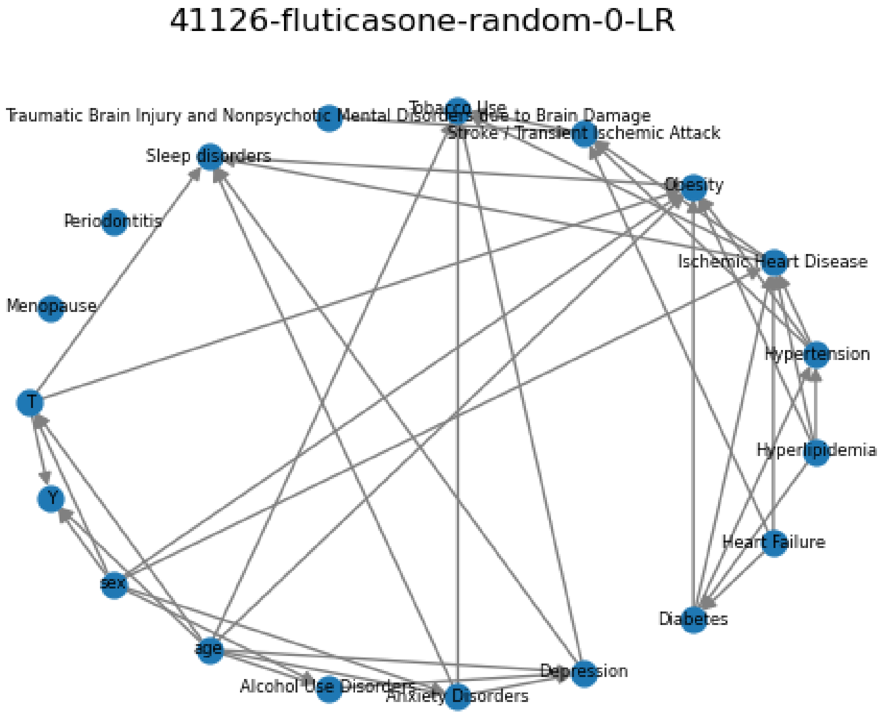

Sup. Method Fig. M7. One example of generated DAG from the fluticasone trial from OneFlorida. The potentially bad controls are detected M-colliders including Sleep disorders, Obesity.

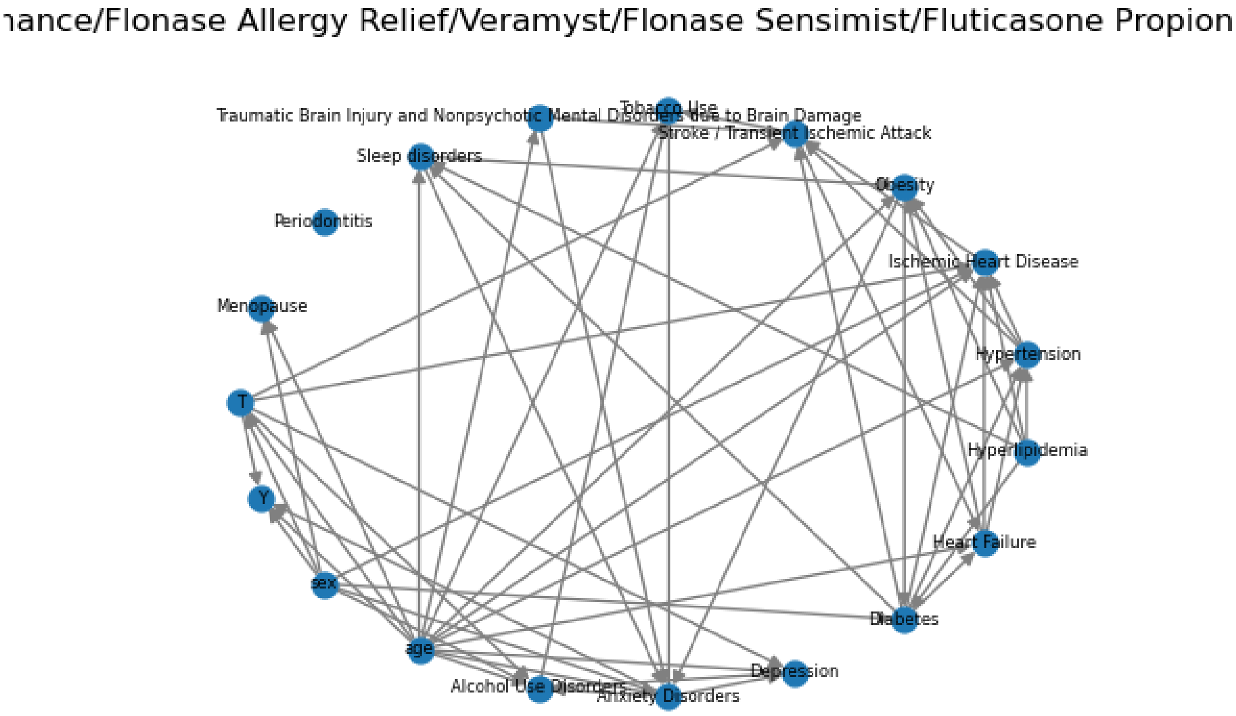

Sup. Method Fig. M8. One example of generated DAG from the fluticasone trial from MarketScan. The potentially bad controls are detected mediators including Anxiety Disorders, Stroke / Transient Ischemic Attack, Heart Failure, Sleep disorders, Obesity, Diabetes, Hypertension, Ischemic Heart Disease, and detected M-colliders including Tobacco Use, Depression, Alcohol Use Disorders.

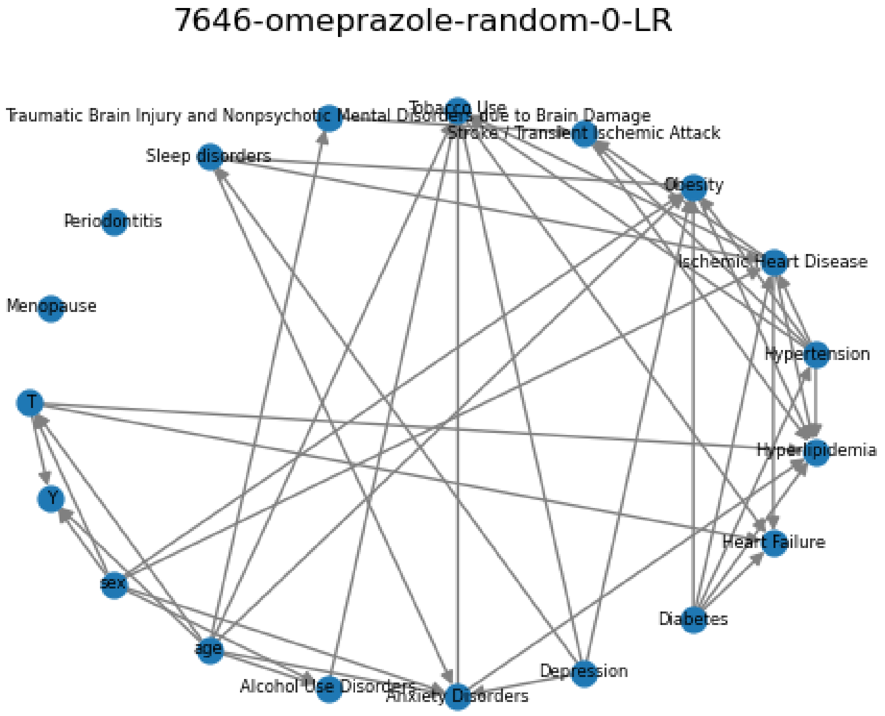

Sup. Method Fig. M9. One example of generated DAG from the omeprazole trial from OneFlorida. The potentially bad controls are detected M-colliders including Hyperlipidemia, Heart Failure.

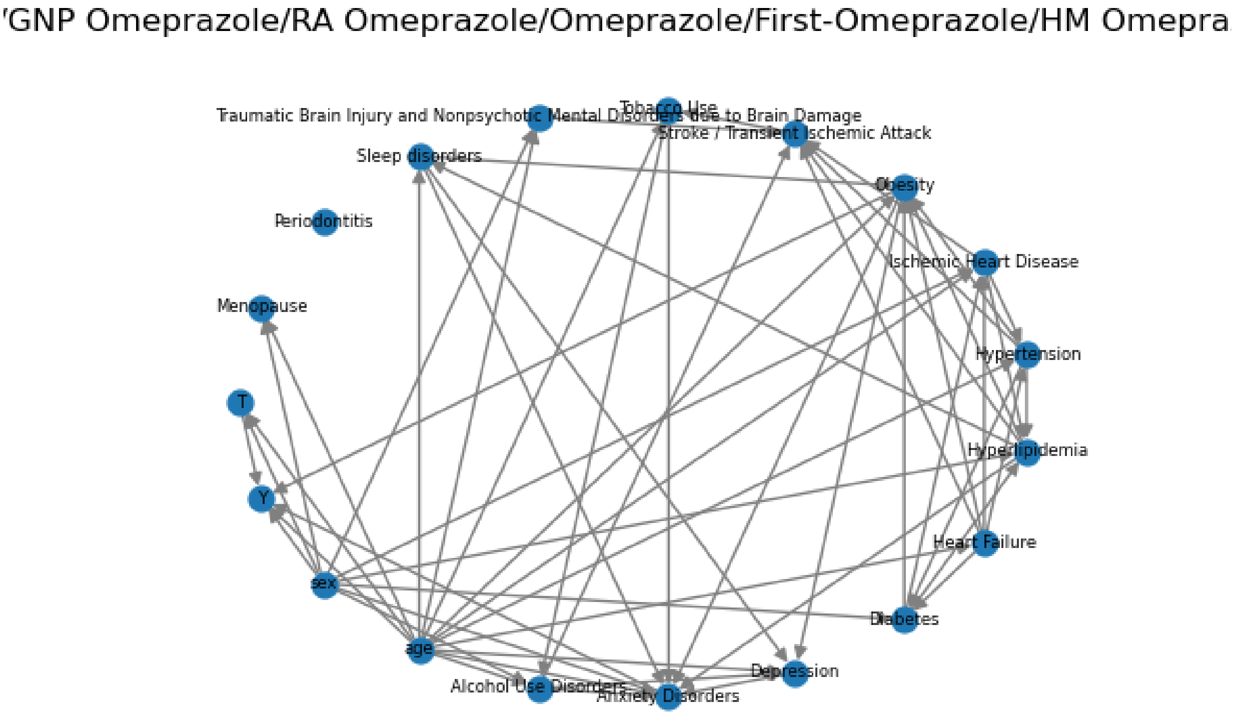

Sup. Method Fig. M10. One example of generated DAG from the omeprazole trial from MarketScan. No potentially bad controls are detected.

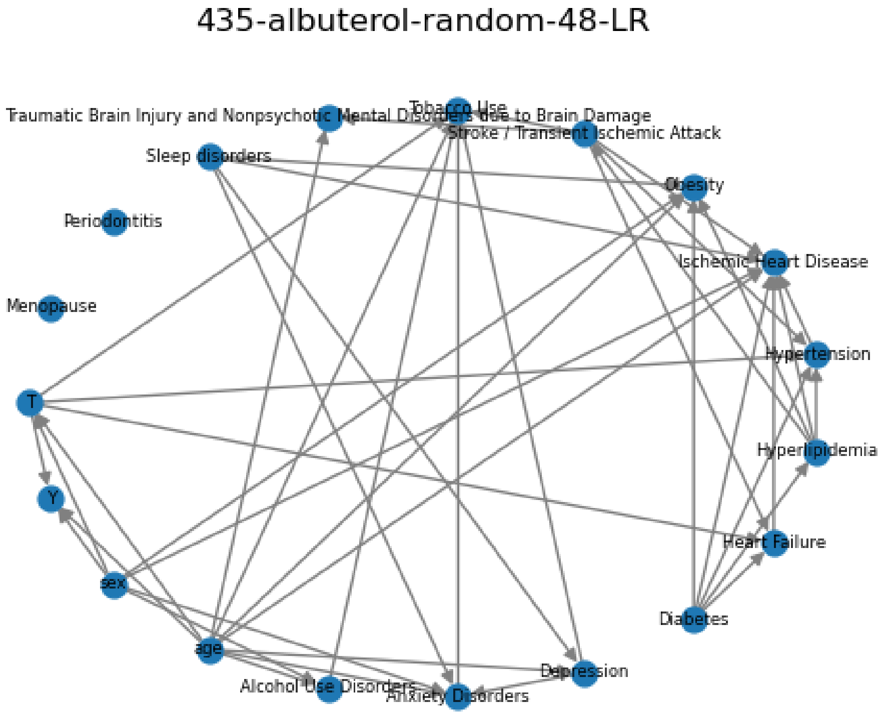

Sup. Method Fig. M11. One example of generated DAG from the albuterol trial from OneFlorida. The potentially bad controls are detected M-colliders including Tobacco Use, Ischemic Heart Disease, Obesity, Hypertension.

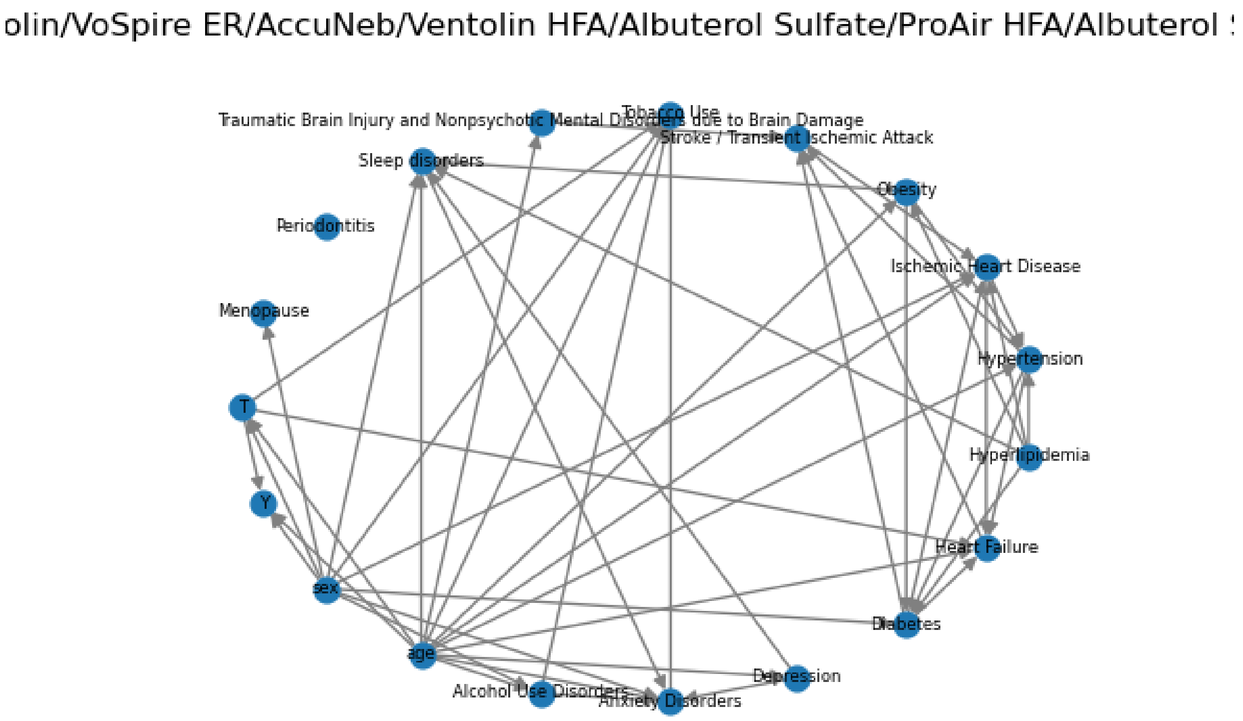

Sup. Method Fig. M12. One example of generated DAG from the albuterol trial from MarketScan. The potentially bad controls are detected M-colliders including Diabetes, Stroke / Transient Ischemic Attack, Ischemic Heart Disease, Hypertension, Tobacco Use, and Heart Failure.

Supplementary Table S4. Baseline comorbidity ICD codes. (at the end due to size)

Comorbidities	ICD codes
ADHD, Conduct Disorders, and Hyperkinetic Syndrome	31200, 31201, 31202, 31203, 31210, 31211, 31212, 31213, 31220, 31221, 31222, 31223, 31230, 31231, 31232, 31233, 31234, 31235, 31239, 3124, 31281, 31282, 31289, 3129, 31400, 31401, 3141, 3142, 3148, 3149, F630, F631, F632, F633, F6381, F6389, F639, F900, F901, F902, F908, F909, F910, F911, F912, F913, F918, F919
Acquired Hypothyroidism	2440, 2441, 2442, 2443, 2448, 2449, E018, E02, E032, E033, E038, E039, E890
Acute Myocardial Infarction	41001, 41011, 41021, 41031, 41041, 41051, 41061, 41071, 41081, 41091, I2101, I2102, I2109, I2111, I2119, I2121, I2129, I213, I214, I219, I21A1, I21A9, I220, I221, I222, I228, I229
Alcohol Use Disorders	2910, 2911, 2912, 2913, 2914, 2915, 2918, 29181, 29182, 29189, 2919, 30300, 30301, 30302, 30390, 30391, 30392, 30500, 30501, 30502, 3575, 4255, 53530, 53531, 5710, 5711, 5712, 5713, 76071, 9800, E8600, F1010, F10120, F10121, F10129, F1014, F10150, F10151, F10159, F10180, F10181, F10182, F10188, F1019, F1020, F10220, F10221, F10229, F10230, F10231, F10232, F10239, F1024, F10250, F10251, F10259, F1026, F1027, F10280, F10281, F10282, F10288, F1029, F10920, F10921, F10929, F1094, F10950, F10951, F10959, F1096, F1097, F10980, F10981, F10982, F10988, F1099, G621, I426, K2920, K2921, K700, K7010, K7011, K702, K7030, K7031, K7040, K7041, K709, P043, Q860, T510X1A, T510X2A, T510X3A, T510X4A, V6542, V791, Z7141, Z7142
Anemia	2800, 2801, 2808, 2809, 2810, 2811, 2812, 2813, 2814, 2818, 2819, 2820, 2821, 2822, 2823, 28240, 28241, 28242, 28243, 28244, 28245, 28246, 28247, 28249, 2825, 28260, 28261, 28262, 28263, 28264, 28268, 28269, 2827, 2828, 2829, 2830, 28310, 28311, 28319, 2832, 2839, 28401, 28409, 28411, 28412, 28419, 2842, 28481, 28489, 2849, 2850, 2851, 28521, 28522, 28529, 2853, 2858, 2859, D500, D501, D508, D509, D510, D511, D512, D513, D518, D519, D520, D521, D528, D529, D530, D531, D532, D538, D539, D550, D551, D552, D553, D558, D559, D560, D561, D562, D563, D564, D565, D568, D569, D5700, D5701, D5702, D571, D5720, D57211, D57212, D57219, D573, D5740, D57411, D57412, D57419, D5780, D57811, D57812, D57819, D580, D581, D582, D588, D589, D590, D591, D592, D593, D594, D595, D596, D598, D599, D600, D601, D608, D609, D6101, D6109, D611, D612, D613, D61810, D61811, D61818, D6182, D6189, D619, D62, D630, D631, D638, D640, D641, D642, D643, D644, D6481, D6489, D649
Anxiety Disorders	29384, 30000, 30001, 30002, 30009, 30010, 30020, 30021, 30022, 30023, 30029, 3003, 3005, 30089, 3009, 3080, 3081, 3082, 3083, 3084, 3089, 30981, 3130, 3131, 31321, 31322, 3133, 31382, 31383, F064, F4000, F4001, F4002, F4010, F4011, F40210, F40218, F40220, F40228, F40230, F40231, F40232, F40233, F40240, F40241, F40242, F40243, F40248, F40290, F40291, F40298, F408, F409, F410, F411, F413, F418, F419, F42, F422, F423, F424, F428, F429, F430, F4310, F4311, F4312, F449, F458, F488, F489, F938, F99, R452, R455, R456, R457
Asthma	49300, 49301, 49302, 49310, 49311, 49312, 49320, 49321, 49322, 49381, 49382, 49390, 49391, 49392, J4520, J4521, J4522, J4530, J4531, J4532, J4540, J4541, J4542, J4550, J4551, J4552, J45901, J45902, J45909, J45990, J45991, J45998
Atrial Fibrillation	42731, I480, I481, I482, I4891

Autism Spectrum Disorders	2990, 29900, 29901, 2991, 29911, 2998, 29980, 29981, 2999, 29990, 29991, F840, F843, F845, F848, F849
Benign Prostatic Hyperplasia	60000, 60001, 60010, 60011, 60020, 60021, 6003, 60090, 60091, N400, N401, N402, N403, N4283
Bipolar Disorder	29600, 29601, 29602, 29603, 29604, 29605, 29606, 29610, 29611, 29612, 29613, 29614, 29615, 29616, 29640, 29641, 29642, 29643, 29644, 29645, 29646, 29650, 29651, 29652, 29653, 29654, 29655, 29656, 29660, 29661, 29662, 29663, 29664, 29665, 29666, 2967, 29680, 29681, 29682, 29689, 29690, 29699, F3010, F3011, F3012, F3013, F302, F303, F304, F308, F309, F310, F3110, F3111, F3112, F3113, F312, F3130, F3131, F3132, F314, F315, F3160, F3161, F3162, F3163, F3164, F3170, F3171, F3172, F3173, F3174, F3175, F3176, F3177, F3178, F3181, F3189, F319, F338, F3481, F3489, F349, F39
Cataract	36601, 36602, 36603, 36604, 36609, 36610, 36612, 36613, 36614, 36615, 36616, 36617, 36618, 36619, 36620, 36621, 36622, 36623, 36630, 36645, 36646, 36650, 36651, 36652, 36653, 3668, 3669, 37926, 37931, 37939, 74330, 74331, 74332, 74333, H25011, H25012, H25013, H25019, H25031, H25032, H25033, H25039, H25041, H25042, H25043, H25049, H25091, H25092, H25093, H25099, H2510, H2511, H2512, H2513, H2520, H2521, H2522, H2523, H25811, H25812, H25813, H25819, H2589, H259, H26011, H26012, H26013, H26019, H26031, H26032, H26033, H26039, H26041, H26042, H26043, H26049, H26051, H26052, H26053, H26059, H26061, H26062, H26063, H26069, H2609, H26101, H26102, H26103, H26109, H26111, H26112, H26113, H26119, H26121, H26122, H26123, H26129, H26131, H26132, H26133, H26139, H2620, H26211, H26212, H26213, H26219, H2630, H2631, H2632, H2633, H2640, H26411, H26412, H26413, H26419, H26491, H26492, H26493, H26499, H268, H269, Q120, V431, Z961
Cerebral Palsy	33371, 343, 3430, 3431, 3432, 3433, 3434, 3438, 3439, G800, G801, G802, G803, G804, G808, G809
Chronic Kidney Disease	01600, 01601, 01602, 01603, 01604, 01605, 01606, 0954, 1890, 1899, 2230, 23691, 24940, 24941, 25040, 25041, 25042, 25043, 2714, 27410, 28311, 40301, 40311, 40391, 40402, 40403, 40412, 40413, 40492, 40493, 4401, 4421, 5724, 5800, 5804, 58081, 58089, 5809, 5810, 5811, 5812, 5813, 58181, 58189, 5819, 5820, 5821, 5822, 5824, 58281, 58289, 5829, 5830, 5831, 5832, 5834, 5836, 5837, 58381, 58389, 5839, 5845, 5846, 5847, 5848, 5849, 5851, 5852, 5853, 5854, 5855, 5856, 5859, 586, 587, 5880, 5881, 58881, 58889, 5889, 591, 75312, 75313, 75314, 75315, 75316, 75317, 75319, 75320, 75321, 75322, 75323, 75329, 7944, A1811, A5275, B520, C641, C642, C649, C689, D3000, D3001, D3002, D4100, D4101, D4102, D4110, D4111, D4112, D4120, D4121, D4122, D593, E0821, E0822, E0829, E0865, E0921, E0922, E0929, E1021, E1022, E1029, E1065, E1121, E1122, E1129, E1165, E1321, E1322, E1329, E748, I120, I129, I130, I1310, I1311, I132, I701, I722, K767, M1030, M10311, M10312, M10319, M10321, M10322, M10329, M10331, M10332, M10339, M10341, M10342, M10349, M10351, M10352, M10359, M10361, M10362, M10369, M10371, M10372, M10379, M1038, M1039, M3214, M3215, M3504, N000, N001, N002, N003, N004, N005, N006, N007, N008, N009, N010, N011, N012, N013, N014, N015, N016, N017, N018, N019, N020, N021, N022, N023, N024, N025, N026, N027, N028, N029, N030, N031, N032, N033, N034, N035, N036, N037, N038, N039, N040, N041, N042, N043, N044, N045, N046, N047, N048, N049, N050, N051, N052, N053, N054, N055, N056, N057, N058, N059, N060, N061, N062, N063, N064, N065, N066, N067, N068, N069, N070, N071, N072, N073, N074, N075, N076, N077, N078, N079, N08, N131, N132, N1330, N1339, N140, N141, N142, N143, N144, N150, N158, N159, N16, N170, N171, N172, N178, N179, N181, N182, N183, N184, N185, N186, N189, N19, N250, N251, N2581, N2589, N259, N261, N269, Q6102, Q6111, Q6119, Q612, Q613, Q614, Q615, Q618, Q620, Q6210, Q6211, Q6212, Q622, Q6231, Q6232, Q6239, R944
Chronic Obstructive Pulmonary Disease and	490, 4910, 4911, 49120, 49121, 49122, 4918, 4919, 4920, 4928, 4940, 4941, 496, J40, J410, J411, J418, J42, J430, J431, J432, J438, J439, J440, J441, J449, J470, J471, J479

Bronchiectasis	
Colorectal Cancer	1530, 1531, 1532, 1533, 1534, 1535, 1536, 1537, 1538, 1539, 1540, 1541, 2303, 2304, C180, C181, C182, C183, C184, C185, C186, C187, C188, C189, C19, C20, D010, D011, D012, V1005, V1006, Z85038, Z85040, Z85048
Cystic Fibrosis and Other Metabolic Developmental Disorders	243, 2552, 2692, 2701, 2702, 2703, 2704, 2706, 2707, 2711, 2770, 27700, 27701, 27702, 27703, 27709, 2776, 27781, 27785, D81810, D841, E000, E001, E002, E009, E030, E031, E250, E258, E259, E569, E700, E701, E7020, E7021, E7029, E7030, E70310, E70311, E70318, E70319, E70320, E70321, E70328, E70329, E70330, E70331, E70338, E70339, E7039, E705, E708, E709, E710, E71110, E71111, E71118, E7119, E712, E71310, E71311, E71312, E71313, E71314, E71318, E7132, E7141, E7210, E7211, E7212, E7219, E7220, E7221, E7222, E7223, E7229, E723, E724, E7250, E7251, E7259, E728, E7420, E7421, E7429, E840, E8411, E8419, E848, E849
Depression	29620, 29621, 29622, 29623, 29624, 29625, 29626, 29630, 29631, 29632, 29633, 29634, 29635, 29636, 29651, 29652, 29653, 29654, 29655, 29656, 29660, 29661, 29662, 29663, 29664, 29665, 29666, 29689, 2980, 3004, 3091, 311, F3130, F3131, F3132, F314, F315, F3160, F3161, F3162, F3163, F3164, F3175, F3176, F3177, F3178, F3181, F320, F321, F322, F323, F324, F325, F329, F330, F331, F332, F333, F3340, F3341, F3342, F338, F339, F341, F4321, F4323
Depressive Disorders	29620, 29621, 29622, 29623, 29624, 29625, 29626, 29630, 29631, 29632, 29633, 29634, 29635, 29636, 3004, 311, F320, F321, F322, F323, F324, F325, F3289, F329, F330, F331, F332, F333, F3340, F3341, F3342, F338, F339, F341, V790

Diabetes	24900, 24901, 24910, 24911, 24920, 24921, 24930, 24931, 24940, 24941, 24950, 24951, 24960, 24961, 24970, 24971, 24980, 24981, 24990, 24991, 25000, 25001, 25002, 25003, 25010, 25011, 25012, 25013, 25020, 25021, 25022, 25023, 25030, 25031, 25032, 25033, 25040, 25041, 25042, 25043, 25050, 25051, 25052, 25053, 25060, 25061, 25062, 25063, 25070, 25071, 25072, 25073, 25080, 25081, 25082, 25083, 25090, 25091, 25092, 25093, 3572, 36201, 36202, 36203, 36204, 36205, 36206, 36641, E0800, E0801, E0810, E0811, E0821, E0822, E0829, E08311, E08319, E08321, E083211, E083212, E083213, E083219, E08329, E083291, E083292, E083293, E083299, E08331, E083311, E083312, E083313, E083319, E08339, E083391, E083392, E083393, E083399, E08341, E083411, E083412, E083413, E083419, E08349, E083491, E083492, E083493, E083499, E08351, E083511, E083512, E083513, E083519, E083521, E083522, E083523, E083529, E083531, E083532, E083533, E083539, E083541, E083542, E083543, E083549, E083551, E083552, E083553, E083559, E08359, E083591, E083592, E083593, E083599, E0836, E0837X1, E0837X2, E0837X3, E0837X9, E0839, E0840, E0841, E0842, E0843, E0844, E0849, E0851, E0852, E0859, E08610, E08618, E08620, E08621, E08622, E08628, E08630, E08638, E08641, E08649, E0865, E0869, E088, E089, E0900, E0901, E0910, E0911, E0921, E0922, E0929, E09311, E09319, E09321, E093211, E093212, E093213, E093219, E09329, E093291, E093292, E093293, E093299, E09331, E093311, E093312, E093313, E093319, E09339, E093391, E093392, E093393, E093399, E09341, E093411, E093412, E093413, E093419, E09349, E093491, E093492, E093493, E093499, E09351, E093511, E093512, E093513, E093519, E093521, E093522, E093523, E093529, E093531, E093532, E093533, E093539, E093541, E093542, E093543, E093549, E093551, E093552, E093553, E093559, E09359, E093591, E093592, E093593, E093599, E0936, E0937X1, E0937X2, E0937X3, E0937X9, E0939, E0940, E0941, E0942, E0943, E0944, E0949, E0951, E0952, E0959, E09610, E09618, E09620, E09621, E09622, E09628, E09630, E09638, E09641, E09649, E0965, E0969, E098, E099, E1010, E1011, E1021, E1022, E1029, E10311, E10319, E10321, E103211, E103212, E103213, E103219, E10329, E103291, E103292, E103293, E103299, E10331, E103311, E103312, E103313, E103319, E10339, E103391, E103392, E103393, E103399, E10341, E103411, E103412, E103413, E103419, E10349, E103491, E103492, E103493, E103499, E10351, E103511, E103512, E103513, E103519, E10359, E1036, E1037X1, E1037X2, E1037X3, E1037X9, E1039, E1040, E1041, E1042, E1043, E1044, E1049, E1051, E1052, E1059, E10610, E10618, E10620, E10621, E10622, E10628, E10630, E10638, E10641, E10649, E1065, E1069, E108, E109, E1100, E1101, E1110, E1111, E1121, E1122, E1129, E11311, E11319, E11321, E113211, E113212, E113213, E113219, E11329, E113291, E113292, E113293, E113299, E11331, E113311, E113312, E113313, E113319, E11339, E113391, E113392, E113393, E113399, E11341, E113411, E113412, E113413, E113419, E11349, E113491, E113492, E113493, E113499, E11351, E113511, E113512, E113513, E113519, E113521, E113522, E113523, E113529, E113531, E113532, E113533, E113539, E113541, E113542, E113543, E113549, E113551, E113552, E113553, E113559, E11359, E113591, E113592, E113593, E113599, E1136, E1137X1, E1137X2, E1137X3, E1137X9, E1139, E1140, E1141, E1142, E1143, E1144, E1149, E1151, E1152, E1159, E11610, E11618, E11620, E11621, E11622, E11628, E11630, E11638, E11641, E11649, E1165, E1169, E118, E119, E1300, E1301, E1310, E1311, E1321, E1322, E1329, E13311, E13319, E13321, E133211, E133212, E133213, E133219, E13329, E133291, E133292, E133293, E133299, E13331, E133311, E133312, E133313, E133319, E13339, E133391, E133392, E133393, E133399, E13341, E133411, E133412, E133413, E133419, E13349, E133491, E133492, E133493, E133499, E13351, E133511, E133512, E133513, E133519, E133521, E133522, E133523, E133529, E133531, E133532, E133533, E133539, E133541, E133542, E133543, E133549, E133551, E133552, E133553, E133559, E13359, E1336, E1339, E1340, E1341, E1342, E1343, E1344, E1349, E1351, E1352, E1359, E13610, E13618, E13620, E13621, E13622, E13628, E13630, E13638, E13641, E13649, E1365, E1369, E138, E139

Drug Use Disorders	2920, 29211, 29212, 2922, 29281, 29282, 29283, 29284, 29285, 29289, 2929, 30400, 30401, 30402, 30410, 30411, 30412, 3042, 30420, 30421, 30422, 3043, 30430, 30431, 30432, 3044, 30440, 30441, 30442, 3045, 30450, 30451, 30452, 3046, 30460, 30461, 30462, 3047, 30470, 30471, 30472, 3048, 30480, 30481, 30482, 3049, 30490, 30491, 30492, 3052, 30520, 30521, 30522, 3053, 30530, 30531, 30532, 3054, 30540, 30541, 30542, 3055, 30550, 30551, 30552, 3056, 30560, 30561, 30562, 3057, 30570, 30571, 30572, 3058, 30580, 30581, 30582, 3059, 30590, 30591, 30592, 6483, 64830, 64831, 64832, 64833, 64834, 6555, 65550, 65551, 65553, 76072, 76073, 76075, 7795, 9650, 96500, 96501, 96502, 96509, E8500, E8501, E8502, E8541, E9350, E9351, F1110, F11120, F11121, F11122, F11129, F1114, F11150, F11151, F11159, F11181, F11182, F11188, F1119, F1120, F11220, F11221, F11222, F11229, F1123, F1124, F11250, F11251, F11259, F11281, F11282, F11288, F1129, F1190, F11920, F11921, F11922, F11929, F1193, F1194, F11950, F11951, F11959, F11981, F11982, F11988, F1199, F1210, F12120, F12121, F12122, F12129, F12150, F12151, F12159, F12180, F12188, F1219, F1220, F12220, F12221, F12222, F12229, F12250, F12251, F12259, F12280, F12288, F1229, F1290, F12920, F12921, F12922, F12929, F12950, F12951, F12959, F12980, F12988, F1299, F1310, F13120, F13121, F13129, F1314, F13150, F13151, F13159, F13180, F13181, F13182, F13188, F1319, F1320, F13220, F13221, F13229, F13230, F13231, F13232, F13239, F1324, F13250, F13251, F13259, F1326, F1327, F13280, F13281, F13282, F13288, F1329, F1390, F13920, F13921, F13929, F13930, F13931, F13932, F13939, F1394, F13950, F13951, F13959, F1396, F1397, F13980, F13981, F13982, F13988, F1399, F1410, F14120, F14121, F14122, F14129, F1414, F14150, F14151, F14159, F14180, F14181, F14182, F14188, F1419, F1420, F14220, F14221, F14222, F14229, F1423, F1424, F14250, F14251, F14259, F14280, F14281, F14282, F14288, F1429, F1490, F14920, F14921, F14922, F14929, F1494, F14950, F14951, F14959, F14980, F14981, F14982, F14988, F1499, F1510, F15120, F15121, F15122, F15129, F1514, F15150, F15151, F15159, F15180, F15181, F15182, F15188, F1519, F1520, F15220, F15221, F15222, F15229, F1523, F1524, F15250, F15251, F15259, F15280, F15281, F15282, F15288, F1529, F1590, F15920, F15921, F15922, F15929, F1593, F1594, F15950, F15951, F15959, F15980, F15981, F15982, F15988, F1599, F1610, F16120, F16121, F16122, F16129, F1614, F16150, F16151, F16159, F16180, F16183, F16188, F1619, F1620, F16220, F16221, F16229, F1624, F16250, F16251, F16259, F16280, F16283, F16288, F1629, F1690, F16920, F16921, F16929, F1694, F16950, F16951, F16959, F16980, F16983, F16988, F1699, F17203, F17208, F17209, F17213, F17218, F17219, F17223, F17228, F17229, F17293, F17298, F17299, F1810, F18120, F18121, F18129, F1814, F18150, F18151, F18159, F1817, F18180, F18188, F1819, F1820, F18220, F18221, F18229, F1824, F18250, F18251, F18259, F1827, F18280, F18288, F1829, F1890, F18920, F18921, F18929, F1894, F18950, F18951, F18959, F1897, F18980, F18988, F1899, F1910, F19120, F19121, F19122, F19129, F1914, F19150, F19151, F19159, F1916, F1917, F19180, F19181, F19182, F19188, F1919, F1920, F19220, F19221, F19222, F19229, F19230, F19231, F19232, F19239, F1924, F19250, F19251, F19259, F1926, F1927, F19280, F19281, F19282, F19288, F1929, F1990, F19920, F19921, F19922, F19929, F19930, F19931, F19932, F19939, F1994, F19950, F19951, F19959, F1996, F1997, F19980, F19981, F19982, F19988, F1999, F550, F551, F552, F553, F554, F558, O355XX0, O355XX1, O355XX2, O355XX3, O355XX4, O355XX5, O355XX9, O99320, O99321, O99322, O99323, O99324, O99325, P0441, P0449, P961, P962, T400X1A, T400X2A, T400X3A, T400X4A, T400X5A, T400X5S, T401X1A, T401X2A, T401X3A, T401X4A, T402X1A, T402X2A, T402X3A, T402X4A, T403X1A, T403X2A, T403X3A, T403X4A, T403X5A, T403X5S, T404X1A, T404X2A, T404X3A, T404X4A, T40601A, T40602A, T40603A, T40604A, T40691A, T40692A, T40693A, T40694A, T407X1A, T408X1A, T40901A, T40991A, V6542, Z7141, Z7142, Z7151, Z7152, Z716
Endometrial Cancer	1820, 2332, C541, C542, C543, C548, C549, D070, V1042, Z8542

Epilepsy	345, 3450, 34500, 34501, 3451, 34510, 34511, 3452, 3453, 3454, 34540, 34541, 3455, 34550, 34551, 3456, 34560, 34561, 3457, 34570, 34571, 3458, 34580, 34581, 3459, 34590, 34591, G40001, G40009, G40011, G40019, G40101, G40109, G40111, G40119, G40201, G40209, G40211, G40219, G40301, G40309, G40311, G40319, G40401, G40409, G40411, G40419, G40501, G40509, G40801, G40802, G40803, G40804, G40811, G40812, G40813, G40814, G40821, G40822, G40823, G40824, G4089, G40901, G40909, G40911, G40919, G40A01, G40A09, G40A11, G40A19, G40B01, G40B09, G40B11, G40B19
Female / Male Breast Cancer	1740, 1741, 1742, 1743, 1744, 1745, 1746, 1748, 1749, 1750, 1759, 2330, C50011, C50012, C50019, C50021, C50022, C50029, C50111, C50112, C50119, C50121, C50122, C50129, C50211, C50212, C50219, C50221, C50222, C50229, C50311, C50312, C50319, C50321, C50322, C50329, C50411, C50412, C50419, C50421, C50422, C50429, C50511, C50512, C50519, C50521, C50522, C50529, C50611, C50612, C50619, C50621, C50622, C50629, C50811, C50812, C50819, C50821, C50822, C50829, C50911, C50912, C50919, C50921, C50922, C50929, D0500, D0501, D0502, D0510, D0511, D0512, D0580, D0581, D0582, D0590, D0591, D0592, V103, Z853
Fibromyalgia, Chronic Pain and Fatigue	3382, 33821, 33822, 33823, 33829, 3383, 3384, 7291, 7292, 7807, 78071, G8921, G8922, G8928, G8929, G893, G894, M5410, M5411, M5412, M5413, M5414, M5415, M5416, M5417, M5418, M6080, M60811, M60812, M60819, M60821, M60822, M60829, M60831, M60832, M60839, M60841, M60842, M60849, M60851, M60852, M60859, M60861, M60862, M60869, M60871, M60872, M60879, M6088, M6089, M609, M791, M7910, M7911, M7912, M7918, M792, M797, R5382

Glaucoma	36285, 36500, 36501, 36502, 36503, 36504, 36510, 36511, 36512, 36513, 36515, 36520, 36521, 36522, 36523, 36524, 36531, 36532, 36541, 36542, 36543, 36551, 36552, 36559, 36560, 36561, 36562, 36563, 36564, 36565, 36581, 36582, 36583, 36589, 3659, 37714, H40001, H40002, H40003, H40009, H40011, H40012, H40013, H40019, H40031, H40032, H40033, H40039, H40041, H40042, H40043, H40049, H40051, H40052, H40053, H40059, H4010X0, H4010X1, H4010X2, H4010X3, H4010X4, H401110, H401111, H401112, H401113, H401114, H401120, H401121, H401122, H401123, H401124, H401130, H401131, H401132, H401133, H401134, H401190, H401191, H401192, H401193, H401194, H4011X0, H4011X1, H4011X2, H4011X3, H4011X4, H401210, H401211, H401212, H401213, H401214, H401220, H401221, H401222, H401223, H401224, H401230, H401231, H401232, H401233, H401234, H401290, H401291, H401292, H401293, H401294, H401310, H401311, H401312, H401313, H401314, H401320, H401321, H401322, H401323, H401324, H401330, H401331, H401332, H401333, H401334, H401390, H401391, H401392, H401393, H401394, H401410, H401411, H401412, H401413, H401414, H401420, H401421, H401422, H401423, H401424, H401430, H401431, H401432, H401433, H401434, H401490, H401491, H401492, H401493, H401494, H40151, H40152, H40153, H40159, H4020X0, H4020X1, H4020X2, H4020X3, H4020X4, H40211, H40212, H40213, H40219, H402210, H402211, H402212, H402213, H402214, H402220, H402221, H402222, H402223, H402224, H402230, H402231, H402232, H402233, H402234, H402290, H402291, H402292, H402293, H402294, H40231, H40232, H40233, H40239, H40241, H40242, H40243, H40249, H4030X0, H4030X1, H4030X2, H4030X3, H4030X4, H4031X0, H4031X1, H4031X2, H4031X3, H4031X4, H4032X0, H4032X1, H4032X2, H4032X3, H4032X4, H4033X0, H4033X1, H4033X2, H4033X3, H4033X4, H4040X0, H4040X1, H4040X2, H4040X3, H4040X4, H4041X0, H4041X1, H4041X2, H4041X3, H4041X4, H4042X0, H4042X1, H4042X2, H4042X3, H4042X4, H4043X0, H4043X1, H4043X2, H4043X3, H4043X4, H4050X0, H4050X1, H4050X2, H4050X3, H4050X4, H4051X0, H4051X1, H4051X2, H4051X3, H4051X4, H4052X0, H4052X1, H4052X2, H4052X3, H4052X4, H4053X0, H4053X1, H4053X2, H4053X3, H4053X4, H4060X0, H4060X1, H4060X2, H4060X3, H4060X4, H4061X0, H4061X1, H4061X2, H4061X3, H4061X4, H4062X0, H4062X1, H4062X2, H4062X3, H4062X4, H4063X0, H4063X1, H4063X2, H4063X3, H4063X4, H40811, H40812, H40813, H40819, H40821, H40822, H40823, H40829, H40831, H40832, H40833, H40839, H4089, H409, H42, H44511, H44512, H44513, H44519, H47231, H47232, H47233, H47239, Q150 ,
Heart Failure	39891, 40201, 40211, 40291, 40401, 40403, 40411, 40413, 40491, 40493, 4280, 4281, 42820, 42821, 42822, 42823, 42830, 42831, 42832, 42833, 42840, 42841, 42842, 42843, 4289, I0981, I110, I130, I132, I501, I5020, I5021, I5022, I5023, I5030, I5031, I5032, I5033, I5040, I5041, I5042, I5043, I50810, I50811, I50812, I50813, I50814, I5082, I5083, I5084, I5089, I509

Hip/Pelvic Fracture	73314, 73315, 73396, 73397, 73398, 8080, 8081, 8082, 8083, 80841, 80842, 80843, 80844, 80849, 80851, 80852, 80853, 80854, 80859, 8088, 8089, 82000, 82001, 82002, 82003, 82009, 82010, 82011, 82012, 82013, 82019, 82020, 82021, 82022, 82030, 82031, 82032, 8208, 8209, M80051A, M80052A, M80059A, M80851A, M80852A, M80859A, M84350A, M84351A, M84352A, M84353A, M84359A, M84451A, M84452A, M84453A, M84459A, M84550A, M84551A, M84552A, M84553A, M84559A, M84650A, M84651A, M84652A, M84653A, M84659A, S32301A, S32301B, S32302A, S32302B, S32309A, S32309B, S32311A, S32311B, S32312A, S32312B, S32313A, S32313B, S32314A, S32314B, S32315A, S32315B, S32316A, S32316B, S32391A, S32391B, S32392A, S32392B, S32399A, S32399B, S32401A, S32401B, S32402A, S32402B, S32409A, S32409B, S32411A, S32411B, S32412A, S32412B, S32413A, S32413B, S32414A, S32414B, S32415A, S32415B, S32416A, S32416B, S32421A, S32421B, S32422A, S32422B, S32423A, S32423B, S32424A, S32424B, S32425A, S32425B, S32426A, S32426B, S32431A, S32431B, S32432A, S32432B, S32433A, S32433B, S32434A, S32434B, S32435A, S32435B, S32436A, S32436B, S32441A, S32441B, S32442A, S32442B, S32443A, S32443B, S32444A, S32444B, S32445A, S32445B, S32446A, S32446B, S32451A, S32451B, S32452A, S32452B, S32453A, S32453B, S32454A, S32454B, S32455A, S32455B, S32456A, S32456B, S32461A, S32461B, S32462A, S32462B, S32463A, S32463B, S32464A, S32464B, S32465A, S32465B, S32466A, S32466B, S32471A, S32471B, S32472A, S32472B, S32473A, S32473B, S32474A, S32474B, S32475A, S32475B, S32476A, S32476B, S32481A, S32481B, S32482A, S32482B, S32483A, S32483B, S32484A, S32484B, S32485A, S32485B, S32486A, S32486B, S32491A, S32491B, S32492A, S32492B, S32499A, S32499B, S32501A, S32501B, S32502A, S32502B, S32509A, S32509B, S32511A, S32511B, S32512A, S32512B, S32519A, S32519B, S32591A, S32591B, S32592A, S32592B, S32599A, S32599B, S32601A, S32601B, S32602A, S32602B, S32609A, S32609B, S32611A, S32611B, S32612A, S32612B, S32613A, S32613B, S32614A, S32614B, S32615A, S32615B, S32616A, S32616B, S32691A, S32691B, S32692A, S32692B, S32699A, S32699B, S32810A, S32810B, S32811A, S32811B, S3282XA, S3282XB, S3289XA, S3289XB, S329XXA, S329XXB, S72001A, S72001B, S72001C, S72002A, S72002B, S72002C, S72009A, S72009B, S72009C, S72011A, S72011B, S72011C, S72012A, S72012B, S72012C, S72019A, S72019B, S72019C, S72021A, S72021B, S72021C, S72022A, S72022B, S72022C, S72023A, S72023B, S72023C, S72024A, S72024B, S72024C, S72025A, S72025B, S72025C, S72026A, S72026B, S72026C, S72031A, S72031B, S72031C, S72032A, S72032B, S72032C, S72033A, S72033B, S72033C, S72034A, S72034B, S72034C, S72035A, S72035B, S72035C, S72036A, S72036B, S72036C, S72041A, S72041B, S72041C, S72042A, S72042B, S72042C, S72043A, S72043B, S72043C, S72044A, S72044B, S72044C, S72045A, S72045B, S72045C, S72046A, S72046B, S72046C, S72051A, S72051B, S72051C, S72052A, S72052B, S72052C, S72059A, S72059B, S72059C, S72061A, S72061B, S72061C, S72062A, S72062B, S72062C, S72063A, S72063B, S72063C, S72064A, S72064B, S72064C, S72065A, S72065B, S72065C, S72066A, S72066B, S72066C, S72091A, S72091B, S72091C, S72092A, S72092B, S72092C, S72099A, S72099B, S72099C, S72101A, S72101B, S72101C, S72102A, S72102B, S72102C, S72109A, S72109B, S72109C, S72111A, S72111B, S72111C, S72112A, S72112B, S72112C, S72113A, S72113B, S72113C, S72114A, S72114B, S72114C, S72115A, S72115B, S72115C, S72116A, S72116B, S72116C, S72121A, S72121B, S72121C, S72122A, S72122B, S72122C, S72123A, S72123B, S72123C, S72124A, S72124B, S72124C, S72125A, S72125B, S72125C, S72126A, S72126B, S72126C, S72131A, S72131B, S72131C, S72132A, S72132B, S72132C, S72133A, S72133B, S72133C, S72134A, S72134B, S72134C, S72135A, S72135B, S72135C, S72136A, S72136B, S72136C, S72141A, S72141B, S72141C, S72142A, S72142B, S72142C, S72143A, S72143B, S72143C, S72144A, S72144B, S72144C, S72145A, S72145B, S72145C, S72146A, S72146B, S72146C, S7221XA, S7221XB, S7221XC, S7222XA, S7222XB, S7222XC, S7223XA, S7223XB, S7223XC, S7224XA, S7224XB, S7224XC, S7225XA, S7225XB, S7225XC, S7226XA, S7226XB, S7226XC, S79001A, S79002A, S79009A, S79011A, S79012A, S79019A, S79091A, S79092A, S79099A
Human Immunodeficiency Virus and/or Acquired Immunodeficiency Syndrome	042, 0420, 0421, 0422, 0429, 043, 0431, 0432, 0433, 0439, 044, 0440, 0449, 07953, 79571, B20, B9735, R75, V08, Z21

(HIV/AIDS)	
Hyperlipidemia	2720, 2721, 2722, 2723, 2724, E780, E7800, E7801, E781, E782, E783, E784, E7841, E7849, E785
Hypertension	36211, 4010, 4011, 4019, 40200, 40201, 40210, 40211, 40290, 40291, 40300, 40301, 40310, 40311, 40390, 40391, 40400, 40401, 40402, 40403, 40410, 40411, 40412, 40413, 40490, 40491, 40492, 40493, 40501, 40509, 40511, 40519, 40591, 40599, 4372, H35031, H35032, H35033, H35039, I10, I110, I119, I120, I129, I130, I1310, I1311, I132, I150, I151, I152, I158, I159, I674, N262
Intellectual Disabilities and Related Conditions	317, 318, 3180, 3181, 3182, 319, 758, 7580, 7581, 7582, 7583, 75831, 75832, 75833, 75839, 7585, 7597, 75981, 75983, 75989, 76071, E7871, E7872, F70, F71, F72, F73, F78, F79, P043, Q860, Q871, Q872, Q873, Q875, Q8781, Q8789, Q897, Q898, Q900, Q901, Q902, Q909, Q910, Q911, Q912, Q913, Q914, Q915, Q916, Q917, Q920, Q921, Q922, Q925, Q9261, Q9262, Q927, Q928, Q929, Q930, Q931, Q932, Q933, Q934, Q935, Q9351, Q93529, Q937, Q9381, Q9388, Q9389, Q939, Q952, Q953, Q992
Ischemic Heart Disease	41000, 41001, 41002, 41010, 41011, 41012, 41020, 41021, 41022, 41030, 41031, 41032, 41040, 41041, 41042, 41050, 41051, 41052, 41060, 41061, 41062, 41070, 41071, 41072, 41080, 41081, 41082, 41090, 41091, 41092, 4110, 4111, 41181, 41189, 412, 4130, 4131, 4139, 41400, 41401, 41402, 41403, 41404, 41405, 41406, 41407, 41412, 4142, 4143, 4144, 4148, 4149, I200, I201, I208, I209, I2101, I2102, I2109, I2111, I2119, I2121, I2129, I213, I214, I21A1, I21A9, I220, I221, I222, I228, I229, I230, I231, I232, I233, I234, I235, I236, I237, I238, I240, I241, I248, I249, I2510, I25110, I25111, I25118, I25119, I252, I253, I2541, I2542, I255, I256, I25700, I25701, I25708, I25709, I25710, I25711, I25718, I25719, I25720, I25721, I25728, I25729, I25730, I25731, I25738, I25739, I25750, I25751, I25758, I25759, I25760, I25761, I25768, I25769, I25790, I25791, I25798, I25799, I25810, I25811, I25812, I2582, I2583, I2584, I2589, I259
Learning Disabilities	315, 31501, 31502, 31509, 3151, 3152, 31531, 31532, 31534, 31535, 31539, 3154, F800, F801, F802, F804, F8081, F8082, F8089, F809, F810, F812, F8181, F8189, F819, F82, H9325, R480
Leukemias and Lymphomas	2000, 20000, 20001, 20002, 20003, 20004, 20005, 20006, 20007, 20008, 2001, 20010, 20011, 20012, 20013, 20014, 20015, 20016, 20017, 20018, 2002, 20020, 20021, 20022, 20023, 20024, 20025, 20026, 20027, 20028, 2003, 20030, 20031, 20032, 20033, 20034, 20035, 20036, 20037, 20038, 2004, 20040, 20041, 20042, 20043, 20044, 20045, 20046, 20047, 20048, 2005, 20050, 20051, 20052, 20053, 20054, 20055, 20056, 20057, 20058, 2006, 20060, 20061, 20062, 20063, 20064, 20065, 20066, 20067, 20068, 2007, 20070, 20071, 20072, 20073, 20074, 20075, 20076, 20077, 20078, 2008, 20080, 20081, 20082, 20083, 20084, 20085, 20086, 20087, 20088, 2010, 20100, 20101, 20102, 20103, 20104, 20105, 20106, 20107, 20108, 2011, 20110, 20111, 20112, 20113, 20114, 20115, 20116, 20117, 20118, 2012, 20120, 20121, 20122, 20123, 20124, 20125, 20126, 20127, 20128, 2014, 20140, 20141, 20142, 20143, 20144, 20145, 20146, 20147, 20148, 2015, 20150, 20151, 20152, 20153, 20154, 20155, 20156, 20157, 20158, 2016, 20160, 20161, 20162, 20163, 20164, 20165, 20166, 20167, 20168, 2017, 20170, 20171, 20172, 20173, 20174, 20175, 20176, 20177, 20178, 2019, 20190, 20191, 20192, 20193, 20194, 20195, 20196, 20197, 20198, 2020, 20200, 20201, 20202, 20203, 20204, 20205, 20206, 20207, 20208, 2021, 20210, 20211, 20212, 20213, 20214, 20215, 20216, 20217, 20218, 2022, 20220, 20221, 20222, 20223, 20224, 20225, 20226, 20227, 20228, 2024, 20240, 20241, 20242, 20243, 20244, 20245, 20246, 20247, 20248, 2027, 20270, 20271, 20272, 20273, 20274, 20275, 20276, 20277, 20278, 2028, 20280, 20281, 20282, 20283, 20284, 20285, 20286, 20287, 20288, 2029, 20290, 20291, 20292, 20293, 20294, 20295, 20296, 20297, 20298, 2031, 20310, 20311, 20312, 2040, 20400,

	20401, 20402, 2041, 20410, 20411, 20412, 2042, 20420, 20421, 20422, 2048, 20480, 20481, 20482, 2049, 20490, 20491, 20492, 2050, 20500, 20501, 20502, 2051, 20510, 20511, 20512, 2052, 20520, 20521, 20522, 2053, 20530, 20531, 20532, 2058, 20580, 20581, 20582, 2059, 20590, 20591, 20592, 2060, 20600, 20601, 20602, 2061, 20610, 20611, 20612, 2062, 20620, 20621, 20622, 2068, 20680, 20681, 20682, 2069, 20690, 20691, 20692, 2070, 20700, 20701, 20702, 2071, 20710, 20711, 20712, 2072, 20720, 20721, 20722, 2078, 20780, 20781, 20782, 2080, 20800, 20801, 20802, 2081, 20810, 20811, 20812, 2082, 20820, 20821, 20822, 2088, 20880, 20881, 20882, 2089, 20890, 20891, 20892, C8100, C8101, C8102, C8103, C8104, C8105, C8106, C8107, C8108, C8109, C8110, C8111, C8112, C8113, C8114, C8115, C8116, C8117, C8118, C8119, C8120, C8121, C8122, C8123, C8124, C8125, C8126, C8127, C8128, C8129, C8130, C8131, C8132, C8133, C8134, C8135, C8136, C8137, C8138, C8139, C8140, C8141, C8142, C8143, C8144, C8145, C8146, C8147, C8148, C8149, C8170, C8171, C8172, C8173, C8174, C8175, C8176, C8177, C8178, C8179, C8190, C8191, C8192, C8193, C8194, C8195, C8196, C8197, C8198, C8199, C8200, C8201, C8202, C8203, C8204, C8205, C8206, C8207, C8208, C8209, C8210, C8211, C8212, C8213, C8214, C8215, C8216, C8217, C8218, C8219, C8220, C8221, C8222, C8223, C8224, C8225, C8226, C8227, C8228, C8229, C8230, C8231, C8232, C8233, C8234, C8235, C8236, C8237, C8238, C8239, C8240, C8241, C8242, C8243, C8244, C8245, C8246, C8247, C8248, C8249, C8250, C8251, C8252, C8253, C8254, C8255, C8256, C8257, C8258, C8259, C8260, C8261, C8262, C8263, C8264, C8265, C8266, C8267, C8268, C8269, C8280, C8281, C8282, C8283, C8284, C8285, C8286, C8287, C8288, C8289, C8290, C8291, C8292, C8293, C8294, C8295, C8296, C8297, C8298, C8299, C8300, C8301, C8302, C8303, C8304, C8305, C8306, C8307, C8308, C8309, C8310, C8311, C8312, C8313, C8314, C8315, C8316, C8317, C8318, C8319, C8330, C8331, C8332, C8333, C8334, C8335, C8336, C8337, C8338, C8339, C8350, C8351, C8352, C8353, C8354, C8355, C8356, C8357, C8358, C8359, C8370, C8371, C8372, C8373, C8374, C8375, C8376, C8377, C8378, C8379, C8380, C8381, C8382, C8383, C8384, C8385, C8386, C8387, C8388, C8389, C8390, C8391, C8392, C8393, C8394, C8395, C8396, C8397, C8398, C8399, C8400, C8401, C8402, C8403, C8404, C8405, C8406, C8407, C8408, C8409, C8410, C8411, C8412, C8413, C8414, C8415, C8416, C8417, C8418, C8419, C8440, C8441, C8442, C8443, C8444, C8445, C8446, C8447, C8448, C8449, C8460, C8461, C8462, C8463, C8464, C8465, C8466, C8467, C8468, C8469, C8470, C8471, C8472, C8473, C8474, C8475, C8476, C8477, C8478, C8479, C8490, C8491, C8492, C8493, C8494, C8495, C8496, C8497, C8498, C8499, C84A0, C84A1, C84A2, C84A3, C84A4, C84A5, C84A6, C84A7, C84A8, C84A9, C84Z0, C84Z1, C84Z2, C84Z3, C84Z4, C84Z5, C84Z6, C84Z7, C84Z8, C84Z9, C8510, C8511, C8512, C8513, C8514, C8515, C8516, C8517, C8518, C8519, C8520, C8521, C8522, C8523, C8524, C8525, C8526, C8527, C8528, C8529, C8580, C8581, C8582, C8583, C8584, C8585, C8586, C8587, C8588, C8589, C8590, C8591, C8592, C8593, C8594, C8595, C8596, C8597, C8598, C8599, C860, C861, C862, C863, C864, C865, C866, C884, C9010, C9011, C9012, C9100, C9101, C9102, C9110, C9111, C9112, C9130, C9131, C9132, C9140, C9141, C9142, C9150, C9151, C9152, C9160, C9161, C9162, C9190, C9191, C9192, C91A0, C91A1, C91A2, C91Z0, C91Z1, C91Z2, C9200, C9201, C9202, C9210, C9211, C9212, C9220, C9221, C9222, C9230, C9231, C9232, C9240, C9241, C9242, C9250, C9251, C9252, C9260, C9261, C9262, C9290, C9291, C9292, C92A0, C92A1, C92A2, C92Z0, C92Z1, C92Z2, C9300, C9301, C9302, C9310, C9311, C9312, C9330, C9331, C9332, C9390, C9391, C9392, C93Z0, C93Z1, C93Z2, C9400, C9401, C9402, C9420, C9421, C9422, C9430, C9431, C9432, C9480, C9481, C9482, C9500, C9501, C9502, C9510, C9511, C9512, C9590, C9591, C9592, C964, C969, C96Z, D45, V 1063, V106, V1060, V1061, V1062, V1069, V107, V1071, V1072, V1079, Z85231, Z856, Z8571, Z8579
Liver Disease, Cirrhosis and Other Liver Conditions (except Viral Hepatitis)	570, 571, 5710, 5711, 5712, 5713, 5715, 5716, 5718, 5719, 572, 5720, 5721, 5722, 5723, 5724, 5728, 573, 5730, 5734, 5735, 5738, 5739, 5761, 7891, K700, K7010, K7011, K702, K7030, K7031, K7040, K7041, K709, K710, K7111, K717, K718, K719, K7200, K7201, K7210, K7211, K7290, K7291, K740, K741, K742, K743, K744, K745, K7460, K7469, K750, K751, K7581, K7589, K759, K760, K761, K762, K763, K765, K766, K767, K7681, K7689, K769, K77, K8030, K8031, K8032, K8033, K8034, K8035, K8036, K8037, K830, R160, R162, V427, Z4823, Z944

Lung Cancer	1622, 1623, 1624, 1625, 1628, 1629, 2312, C3400, C3401, C3402, C3410, C3411, C3412, C342, C3430, C3431, C3432, C3480, C3481, C3482, C3490, C3491, C3492, D0220, D0221, D0222, V1011, Z85110, Z85118
Migraine and Chronic Headache	339, 3390, 33900, 33901, 33902, 33903, 33904, 33905, 33909, 3391, 33910, 33911, 33912, 3392, 33920, 33921, 33922, 3393, 3394, 33941, 33942, 33943, 33944, 3398, 33981, 33982, 33983, 33984, 33985, 33989, 346, 3460, 34600, 34601, 34602, 34603, 3461, 34610, 34611, 34612, 34613, 3462, 34620, 34621, 34622, 34623, 3463, 34630, 34631, 34632, 34633, 3464, 34640, 34641, 34642, 34643, 3465, 34650, 34651, 34652, 34653, 3466, 34660, 34661, 34662, 34663, 3467, 34670, 34671, 34672, 34673, 3468, 34680, 34681, 34682, 34683, 3469, 34690, 34691, 34692, 34693, G43001, G43009, G43011, G43019, G43101, G43109, G43111, G43119, G43401, G43409, G43411, G43419, G43501, G43509, G43511, G43519, G43601, G43609, G43611, G43619, G43701, G43709, G43711, G43719, G43801, G43809, G43811, G43819, G43821, G43829, G43831, G43839, G43901, G43909, G43911, G43919, G43A0, G43A1, G43B0, G43B1, G43C0, G43C1, G43D0, G43D1, G44001, G44009, G44011, G44019, G44021, G44029, G44031, G44039, G44041, G44049, G44051, G44059, G44091, G44099, G441, G44201, G44209, G44211, G44219, G44221, G44229, G44301, G44309, G44311, G44319, G44321, G44329, G4440, G4441, G4451, G4452, G4453, G4459, G4481, G4482, G4483, G4484, G4485, G4489
Mobility Impairments	3341, 34200, 34201, 34202, 34210, 34211, 34212, 34280, 34281, 34282, 34290, 34291, 34292, 344, 3440, 34400, 34401, 34402, 34403, 34404, 34409, 3441, 3442, 3443, 34430, 34431, 34432, 3444, 34440, 34441, 34442, 3445, 3446, 34460, 34461, 3448, 34481, 34489, 3449, 43820, 43821, 43822, 43830, 43831, 43832, 43840, 43841, 43842, 43850, 43851, 43852, 43853, G041, G114, G8100, G8101, G8102, G8103, G8104, G8110, G8111, G8112, G8113, G8114, G8190, G8191, G8192, G8193, G8194, G8220, G8221, G8222, G8250, G8251, G8252, G8253, G8254, G830, G8310, G8311, G8312, G8313, G8314, G8320, G8321, G8322, G8323, G8324, G8330, G8331, G8332, G8333, G8334, G834, G835, G8381, G8382, G8383, G8384, G8389, G839, I69031, I69032, I69033, I69034, I69039, I69041, I69042, I69043, I69044, I69049, I69051, I69052, I69053, I69054, I69059, I69061, I69062, I69063, I69064, I69065, I69069, I69131, I69132, I69133, I69134, I69139, I69141, I69142, I69143, I69144, I69149, I69151, I69152, I69153, I69154, I69159, I69161, I69162, I69163, I69164, I69165, I69169, I69231, I69232, I69233, I69234, I69239, I69241, I69242, I69243, I69244, I69249, I69251, I69252, I69253, I69254, I69259, I69261, I69262, I69263, I69264, I69265, I69269, I69331, I69332, I69333, I69334, I69339, I69341, I69342, I69343, I69344, I69349, I69351, I69352, I69353, I69354, I69359, I69361, I69362, I69363, I69364, I69365, I69369, I69831, I69832, I69833, I69834, I69839, I69841, I69842, I69843, I69844, I69849, I69851, I69852, I69853, I69854, I69859, I69861, I69862, I69863, I69864, I69865, I69869, I69931, I69932, I69933, I69934, I69939, I69941, I69942, I69943, I69944, I69949, I69951, I69952, I69953, I69954, I69959, I69961, I69962, I69963, I69964, I69965,<br>I69969<br><br>,
Multiple Sclerosis and Transverse Myelitis	340, 341, 3410, 3412, 34120, 34121, 34122, 3418, 3419, G35, G360, G361, G368, G369, G371, G372, G373, G374, G378, G379
Muscular Dystrophy	359, 3590, 3591, G710, G7100, G7101, G7102, G7109, G7111, G712
Obesity	2780, 27800, 27801, 27803, E6601, E6609, E661, E662, E668, E669, V853, V8530, V8531, V8532, V8533, V8534, V8535, V8536, V8537, V8538, V8539, V854, V8541, V8542, V8543, V8544, V8545, Z6830, Z6831, Z6832, Z6833, Z6834, Z6835, Z6836, Z6837, Z6838, Z6839, Z6841, Z6842, Z6843, Z6844, Z6845

Opioid Use Disorder	11288, 3040, 30400, 30401, 30402, 3047, 30470, 30471, 30472, 3055, 30550, 30551, 30552, 76072, 9650, 96500, 96501, 96502, 96509, 9701, E8500, E8501, E8502, E9350, E9351, E9352, E9401, F1110, F11120, F11121, F11122, F11129, F1114, F11150, F11151, F11159, F11181, F11182, F11188, F1119, F1120, F11220, F11221, F11222, F11229, F1123, F1124, F11250, F11251, F11259, F11281, F11282, F11288, F1129, F1190, F11920, F11921, F11922, F11929, F1193, F1194, F11950, F11951, F11959, F11981, F11982, F11988, F1199, J0571, J0572, J0573, J0574, J0575, J1230, J2315, S0109, T400X1A, T400X1D, T400X1S, T400X2A, T400X2D, T400X2S, T400X3A, T400X3D, T400X3S, T400X4A, T400X4D, T400X4S, T400X5A, T400X5D, T400X5S, T401X1A, T401X1D, T401X1S, T401X2A, T401X2D, T401X2S, T401X3A, T401X3D, T401X3S, T401X4A, T401X4D, T401X4S, T402X1A, T402X1D, T402X1S, T402X2A, T402X2D, T402X2S, T402X3A, T402X3D, T402X3S, T402X4A, T402X4D, T402X4S, T402X5A, T402X5D, T402X5S, T403X1A, T403X1D, T403X1S, T403X2A, T403X2D, T403X2S, T403X3A, T403X3D, T403X3S, T403X4A, T403X4D, T403X4S, T403X5A, T403X5D, T403X5S, T404X1A, T404X1D, T404X1S, T404X2A, T404X2D, T404X2S, T404X3A, T404X3D, T404X3S, T404X4A, T404X4D, T404X4S, T404X5A, T404X5D, T404X5S, T40601A, T40601D, T40601S, T40602A, T40602D, T40602S, T40603A, T40603D, T40603S, T40604A, T40604D, T40604S, T40605A, T40605D, T40605S, T40691A, T40691D, T40691S, T40692A, T40692D, T40692S, T40693A, T40693D, T40693S, T40694A, T40694D, T40694S, T40695A, T40695D, T40695S
Osteoporosis	73300, 73301, 73302, 73303, 73309, M810, M816, M818
Other Developmental Delays	3155, 3158, 3159, F819, F82, F88, F89
Peripheral Vascular Disease (PVD)	4400, 4401, 4402, 44020, 44021, 44022, 44023, 44029, 4404, 4438, 44381, 44382, 44389, 4439, E0851, E0852, E0951, E0952, E1051, E1052, E1151, E1152, E1351, E1352, I700, I701, I70201, I70202, I70203, I70208, I70209, I70211, I70212, I70213, I70218, I70219, I70221, I70222, I70223, I70228, I70229, I70231, I70232, I70233, I70234, I70235, I70238, I70239, I70241, I70242, I70243, I70244, I70245, I70248, I70249, I7025, I70291, I70292, I70293, I70298, I70299, I7092, I7381, I7389, I739, I791, I798
Personality Disorders	3010, 30110, 30111, 30112, 30113, 30120, 30121, 30122, 3013, 3014, 30150, 30151, 30159, 3016, 3017, 30181, 30182, 30183, 30184, 30189, 3019, F21, F340, F341, F600, F601, F602, F603, F604, F605, F606, F607, F6081, F6089, F609, F6810, F6811, F6812, F6813, F69
Post-Traumatic Stress Disorder (PTSD)	30981, F4310, F4311, F4312

Pressure and Chronic Ulcers	7070, 70700, 70701, 70702, 70703, 70704, 70705, 70706, 70707, 70709, 7071, 70710, 70711, 70712, 70713, 70714, 70715, 70719, 7072, 70722, 70723, 70724, 70725, 7078, 7079, I70231, I70232, I70233, I70234, I70235, I70238, I70239, I70241, I70242, I70243, I70244, I70245, I70248, I70249, I7025, I70331, I70332, I70333, I70334, I70335, I70338, I70339, I70341, I70342, I70343, I70344, I70345, I70348, I70349, I7035, I70431, I70432, I70433, I70434, I70435, I70438, I70439, I70441, I70442, I70443, I70444, I70445, I70448, I70449, I7045, I70531, I70532, I70533, I70534, I70535, I70538, I70539, I70541, I70542, I70543, I70544, I70545, I70548, I70549, I7055, I70631, I70632, I70633, I70634, I70635, I70638, I70639, I70641, I70642, I70643, I70644, I70645, I70648, I70649, I7065, I70731, I70732, I70733, I70734, I70735, I70738, I70739, I70741, I70742, I70743, I70744, I70745, I70748, I70749, I7075, L89000, L89001, L89002, L89003, L89004, L89009, L89010, L89011, L89012, L89013, L89014, L89019, L89020, L89021, L89022, L89023, L89024, L89029, L89100, L89101, L89102, L89103, L89104, L89109, L89110, L89111, L89112, L89113, L89114, L89119, L89120, L89121, L89122, L89123, L89124, L89129, L89130, L89131, L89132, L89133, L89134, L89139, L89140, L89141, L89142, L89143, L89144, L89149, L89150, L89151, L89152, L89153, L89154, L89159, L89200, L89201, L89202, L89203, L89204, L89209, L89210, L89211, L89212, L89213, L89214, L89219, L89220, L89221, L89222, L89223, L89224, L89229, L89300, L89301, L89302, L89303, L89304, L89309, L89310, L89311, L89312, L89313, L89314, L89319, L89320, L89321, L89322, L89323, L89324, L89329, L8940, L8941, L8942, L8943, L8944, L8945, L89500, L89501, L89502, L89503, L89504, L89509, L89510, L89511, L89512, L89513, L89514, L89519, L89520, L89521, L89522, L89523, L89524, L89529, L89600, L89601, L89602, L89603, L89604, L89609, L89610, L89611, L89612, L89613, L89614, L89619, L89620, L89621, L89622, L89623, L89624, L89629, L89810, L89811, L89812, L89813, L89814, L89819, L89890, L89891, L89892, L89893, L89894, L89899, L8990, L8991, L8992, L8993, L8994, L8995, L97101, L97102, L97103, L97104, L97105, L97106, L97108, L97109, L97111, L97112, L97113, L97114, L97115, L97116, L97118, L97119, L97121, L97122, L97123, L97124, L97125, L97126, L97128, L97129, L97201, L97202, L97203, L97204, L97205, L97206, L97208, L97209, L97211, L97212, L97213, L97214, L97215, L97216, L97218, L97219, L97221, L97222, L97223, L97224, L97225, L97226, L97228, L97229, L97301, L97302, L97303, L97304, L97305, L97306, L97308, L97309, L97311, L97312, L97313, L97314, L97315, L97316, L97318, L97319, L97321, L97322, L97323, L97324, L97325, L97326, L97328, L97329, L97401, L97402, L97403, L97404, L97405, L97406, L97408, L97409, L97411, L97412, L97413, L97414, L97415, L97416, L97418, L97419, L97421, L97422, L97423, L97424, L97425, L97426, L97428, L97429, L97501, L97502, L97503, L97504, L97505, L97506, L97508, L97509, L97511, L97512, L97513, L97514, L97515, L97516, L97518, L97519, L97521, L97522, L97523, L97524, L97525, L97526, L97528, L97529, L97801, L97802, L97803, L97804, L97805, L97806, L97808, L97809, L97811, L97812, L97813, L97814, L97815, L97816, L97818, L97819, L97821, L97822, L97823, L97824, L97825, L97826, L97828, L97829, L97901, L97902, L97903, L97904, L97905, L97906, L97908, L97909, L97911, L97912, L97913, L97914, L97915, L97916, L97918, L97919, L97921, L97922, L97923, L97924, L97925, L97926, L97928, L97929, L98411, L98412, L98413, L98414, L98415, L98416, L98418, L98419, L98421, L98422, L98423, L98424, L98425, L98426, L98428, L98429, L98491, L98492, L98493, L98494, L98495, L98496, L98498, L98499 ,
Prostate Cancer	185, 2334, C61, D075, V1046, Z8546
RA/OA (Rheumatoid Arthritis/ Osteoarthritis)	7140, 7141, 7142, 71430, 71431, 71432, 71433, 71500, 71504, 71509, 71510, 71511, 71512, 71513, 71514, 71515, 71516, 71517, 71518, 71520, 71521, 71522, 71523, 71524, 71525, 71526, 71527, 71528, 71530, 71531, 71532, 71533, 71534, 71535, 71536, 71537, 71538, 71580, 71589, 71590, 71591, 71592, 71593, 71594, 71595, 71596, 71597, 71598, 7200, 7210, 7211, 7212, 7213, 72190, 72191, M0500, M05011, M05012, M05019, M05021, M05022, M05029, M05031, M05032, M05039, M05041, M05042, M05049, M05051, M05052, M05059, M05061, M05062, M05069, M05071, M05072, M05079, M0509, M0520, M05211, M05212, M05219, M05221, M05222, M05229, M05231, M05232, M05239, M05241, M05242, M05249, M05251, M05252, M05259, M05261, M05262, M05269, M05271, M05272, M05279, M0529, M0530, M05311, M05312, M05319, M05321, M05322, M05329, M05331, M05332, M05339,

	M05341, M05342, M05349, M05351, M05352, M05359, M05361, M05362, M05369, M05371, M05372, M05379, M0539, M0540, M05411, M05412, M05419, M05421, M05422, M05429, M05431, M05432, M05439, M05441, M05442, M05449, M05451, M05452, M05459, M05461, M05462, M05469, M05471, M05472, M05479, M0549, M0550, M05511, M05512, M05519, M05521, M05522, M05529, M05531, M05532, M05539, M05541, M05542, M05549, M05551, M05552, M05559, M05561, M05562, M05569, M05571, M05572, M05579, M0559, M0560, M05611, M05612, M05619, M05621, M05622, M05629, M05631, M05632, M05639, M05641, M05642, M05649, M05651, M05652, M05659, M05661, M05662, M05669, M05671, M05672, M05679, M0569, M0570, M05711, M05712, M05719, M05721, M05722, M05729, M05731, M05732, M05739, M05741, M05742, M05749, M05751, M05752, M05759, M05761, M05762, M05769, M05771, M05772, M05779, M0579, M0580, M05811, M05812, M05819, M05821, M05822, M05829, M05831, M05832, M05839, M05841, M05842, M05849, M05851, M05852, M05859, M05861, M05862, M05869, M05871, M05872, M05879, M0589, M059, M0600, M06011, M06012, M06019, M06021, M06022, M06029, M06031, M06032, M06039, M06041, M06042, M06049, M06051, M06052, M06059, M06061, M06062, M06069, M06071, M06072, M06079, M0608, M0609, M061, M0620, M06211, M06212, M06219, M06221, M06222, M06229, M06231, M06232, M06239, M06241, M06242, M06249, M06251, M06252, M06259, M06261, M06262, M06269, M06271, M06272, M06279, M0628, M0629, M0630, M06311, M06312, M06319, M06321, M06322, M06329, M06331, M06332, M06339, M06341, M06342, M06349, M06351, M06352, M06359, M06361, M06362, M06369, M06371, M06372, M06379, M0638, M0639, M0680, M06811, M06812, M06819, M06821, M06822, M06829, M06831, M06832, M06839, M06841, M06842, M06849, M06851, M06852, M06859, M06861, M06862, M06869, M06871, M06872, M06879, M0688, M0689, M069, M0800, M08011, M08012, M08019, M08021, M08022, M08029, M08031, M08032, M08039, M08041, M08042, M08049, M08051, M08052, M08059, M08061, M08062, M08069, M08071, M08072, M08079, M0808, M0809, M081, M0820, M08211, M08212, M08219, M08221, M08222, M08229, M08231, M08232, M08239, M08241, M08242, M08249, M08251, M08252, M08259, M08261, M08262, M08269, M08271, M08272, M08279, M0828, M0829, M083, M0840, M08411, M08412, M08419, M08421, M08422, M08429, M08431, M08432, M08439, M08441, M08442, M08449, M08451, M08452, M08459, M08461, M08462, M08469, M08471, M08472, M08479, M0848, M0880, M08811, M08812, M08819, M08821, M08822, M08829, M08831, M08832, M08839, M08841, M08842, M08849, M08851, M08852, M08859, M08861, M08862, M08869, M08871, M08872, M08879, M0888, M0889, M0890, M08911, M08912, M08919, M08921, M08922, M08929, M08931, M08932, M08939, M08941, M08942, M08949, M08951, M08952, M08959, M08961, M08962, M08969, M08971, M08972, M08979, M0898, M0899, M150, M151, M152, M153, M154, M158, M159, M160, M1610, M1611, M1612, M162, M1630, M1631, M1632, M164, M1650, M1651, M1652, M166, M167, M169, M170, M1710, M1711, M1712, M172, M1730, M1731, M1732, M174, M175, M179, M180, M1810, M1811, M1812, M182, M1830, M1831, M1832, M184, M1850, M1851, M1852, M189, M19011, M19012, M19019, M19021, M19022, M19029, M19031, M19032, M19039, M19041, M19042, M19049, M19071, M19072, M19079, M19111, M19112, M19119, M19121, M19122, M19129, M19131, M19132, M19139, M19141, M19142, M19149, M19171, M19172, M19179, M19211, M19212, M19219, M19221, M19222, M19229, M19231, M19232, M19239, M19241, M19242, M19249, M19271, M19272, M19279, M1990, M1991, M1992, M1993, M450, M451, M452, M453, M454, M455, M456, M457, M458, M459, M47011, M47012, M47013, M47014, M47015, M47016, M47019, M47021, M47022, M47029, M4710, M4711, M4712, M4713, M4720, M4721, M4722, M4723, M4724, M4725, M4726, M4727, M4728, M47811, M47812, M47813, M47814, M47815, M47816, M47817, M47818, M47819, M47891, M47892, M47893, M47894, M47895, M47896, M47897, M47898, M47899, M479, M488X1, M488X2, M488X3, M488X4, M488X5, M488X6, M488X7, M488X8, M488X9 ,
Schizophrenia	29500, 29501, 29502, 29503, 29504, 29505, 29510, 29511, 29512, 29513, 29514, 29515, 29520, 29521, 29522, 29523, 29524, 29525, 29530, 29531, 29532, 29533, 29534, 29535, 29540, 29541, 29542, 29543, 29544, 29545, 29550, 29551, 29552, 29553, 29554, 29555, 29560, 29561, 29562, 29563, 29564, 29565, 29570, 29571, 29572, 29573, 29574, 29575, 29580, 29581, 29582, 29583, 29584, 29585, 29590, 29591, 29592, 29593, 29594, 29595, F200, F201, F202, F203, F205, F2081, F2089, F209, F250, F251, F258, F259

Schizophrenia and Other Psychotic Disorders	29381, 29382, 29500, 29501, 29502, 29503, 29504, 29505, 29510, 29511, 29512, 29513, 29514, 29515, 29520, 29521, 29522, 29523, 29524, 29525, 29530, 29531, 29532, 29533, 29534, 29535, 29540, 29541, 29542, 29543, 29544, 29545, 29550, 29551, 29552, 29553, 29554, 29555, 29560, 29561, 29562, 29563, 29564, 29565, 29570, 29571, 29572, 29573, 29574, 29575, 29580, 29581, 29582, 29583, 29584, 29585, 29590, 29591, 29592, 29593, 29594, 29595, 2970, 2971, 2972, 2973, 2978, 2979, 2980, 2981, 2982, 2983, 2984, 2988, 2989, F060, F062, F200, F201, F202, F203, F205, F2081, F2089, F209, F21, F22, F23, F24, F250, F251, F258, F259, F28, F29, F323, F333, F4489
Sensory - Blindness and Visual Impairment	369, 3690, 36900, 36901, 36902, 36903, 36904, 36905, 36906, 36907, 36908, 3691, 36910, 36911, 36912, 36913, 36914, 36915, 36916, 36917, 36918, 3692, 36920, 36921, 36922, 36923, 36924, 36925, 3693, 3694, H540, H540X33, H540X34, H540X35, H540X43, H540X44, H540X45, H540X53, H540X54, H540X55, H5410, H5411, H541131, H541132, H541141, H541142, H541151, H541152, H5412, H541213, H541214, H541215, H541223, H541224, H541225, H542, H542X11, H542X12, H542X21, H542X22, H543, H548
Sensory – Deafness and Hearing Impairment	389, 3891, 38910, 38911, 38912, 38913, 38914, 38915, 38916, 38917, 38918, 3892, 38920, 38921, 38922, 3897, 3898, 3899, H903, H9041, H9042, H905, H906, H9071, H9072, H908, H90A21, H90A22, H90A31, H90A32, H9101, H9102, H9103, H9109, H913, H918X1, H918X2, H918X3, H918X9, H9190, H9191, H9192, H9193
Sickle Cell Disease	28241, 28242, 28260, 28261, 28262, 28263, 28264, 28268, 28269, D5700, D5701, D5702, D571, D5720, D57211, D57212, D57219, D5740, D57411, D57412, D57419, D5780, D57811, D57812, D57819
Spina Bifida and Other Congenital Anomalies of the Nervous System	7400, 7401, 7402, 741, 7410, 74100 74101, 74102, 74103, 7419, 74190, 74191 74192, 74193, 7420, 7421, 7422, 7423, 7424, 7425, 74251, 74253, 74259, 7428, 7429, G901, Q000, Q001, Q002, Q010, Q011, Q012, Q018, Q019, Q02, Q030, Q031, Q038, Q039, Q040, Q041, Q042, Q043, Q044, Q045, Q046, Q048, Q049, Q050, Q051, Q052, Q053, Q054, Q055, Q056, Q057, Q058, Q059, Q060, Q061, Q062, Q063, Q064, Q068, Q069, Q0700, Q0701, Q0702, Q0703, Q078, Q079

Spinal Cord Injury	15, 34939, 80600 80601, 80602, 80603, 80604, 80605, 80606, 80607, 80608, 80609, 80610, 80611, 80612, 80613, 80614, 80615, 80616, 80617, 80618, 80619, 80620, 80621, 80622, 80623, 80624, 80625, 80626, 80627, 80628, 80629, 80630, 80631, 80632, 80633, 80634, 80635, 80636, 80637, 80638, 80639, 8064, 8065, 80660, 80661, 80662, 80669, 80670, 80671, 80672, 80679, 8068, 8069, 9072, 952, 95200, 95201, 95202, 95203, 95204, 95205, 95206, 95207, 95208, 95209, 95210, 95211, 95212, 95213, 95214, 95216, 95217, 95218, 95219, 9522, 9523, 9524, 9528, 9529, G9611, S12000A, S12000B, S12001A, S12001B, S12100A, S12100B, S12101A, S12101B, S12200A, S12200B, S12201A, S12201B, S12300A, S12300B, S12301A, S12301B, S12400A, S12400B, S12401A, S12401B, S12500A, S12500B, S12501A, S12501B, S12600A, S12600B, S12601A, S12601B, S129XXA, S13113A, S140XXA, S140XXS, S14101A, S14101S, S14102A, S14102S, S14103A, S14103S, S14104A, S14104S, S14105A, S14105S, S14106A, S14106S, S14107A, S14107S, S14108A, S14108S, S14109A, S14109S, S14111A, S14111S, S14112A, S14112S, S14113S, S14114A, S14114S, S14115A, S14115S, S14116A, S14116S, S14117A, S14117S, S14118A, S14118S, S14119A, S14119S, S14121A, S14121S, S14122A, S14122S, S14123A, S14123S, S14124A, S14124S, S14125A, S14125S, S14126A, S14126S, S14127A, S14127S, S14128A, S14128S, S14129A, S14129S, S14131A, S14131S, S14132A, S14132S, S14133A, S14133S, S14134A, S14134S, S14135A, S14135S, S14136A, S14136S, S14137A, S14137S, S14138A, S14138S, S14139A, S14139S, S14141A, S14141S, S14142A, S14142S, S14143A, S14143S, S14144A, S14144S, S14145A, S14145S, S14146A, S14146S, S14147A, S14147S, S14148A, S14148S, S14149A, S14149S, S14151A, S14151S, S14152A, S14152S, S14153A, S14153S, S14154A, S14154S, S14155A, S14155S, S14156A, S14156S, S14157A, S14157S, S14158A, S14158S, S14159A, S14159S, S22009A, S22009B, S22019A, S22019B, S22029A, S22029B, S22039A, S22039B, S22049A, S22049B, S22059A, S22059B, S22069A, S22069B, S22079A, S22079B, S22089A, S22089B, S240XXA, S240XXS, S24101A, S24101S, S24102A, S24102S, S24103A, S24103S, S24104A, S24104S, S24109A, S24109S, S24111A, S24111S, S24112A, S24112S, S24113A, S24113S, S24114A, S24114S, S24119A, S24119S, S24131A, S24131S, S24132A, S24132S, S24133A, S24133S, S24134A, S24134S, S24139A, S24139S, S24141A, S24141S, S24142A, S24142S, S24143A, S24143S, S24144A, S24144S, S24149A, S24149S, S24151A, S24151S, S24152A, S24152S, S24153A, S24153S, S24154A, S24154S, S24159A, S24159S, S32009A, S32009B, S32019A, S32019B, S32029A, S32029B, S32039A, S32039B, S32049A, S32049B, S32059A, S32059B, S3210XA, S3210XB, S322XXA, S322XXB, S3401XA, S3401XS, S3402XA, S3402XS, S34101A, S34101S, S34102A, S34102S, S34103A, S34103S, S34104A, S34104S, S34105A, S34105S, S34109A, S34109S, S34111A, S34111S, S34112A, S34112S, S34113A, S34113S, S34114A, S34114S, S34115A, S34115S, S34119A, S34119S, S34121A, S34121S, S34122A, S34122S, S34123A, S34123S, S34124A, S34124S, S34125A, S34125S, S34129A, S34129S, S34131A, S34131S, S34132A, S34132S, S34139A, S34139S, S343XXA
Stroke / Transient Ischemic Attack	430, 431, 43301, 43311, 43321, 43331, 43381, 43391, 43400, 43401, 43410, 43411, 43490, 43491, 4350, 4351, 4353, 4358, 4359, 436, 99702, G450, G451, G452, G458, G459, G460, G461, G462, G463, G464, G465, G466, G467, G468, G9731, G9732, I6000, I6001, I6002, I6010, I6011, I6012, I6020, I6021, I6022, I6030, I6031, I6032, I604, I6050, I6051, I6052, I606, I607, I608, I609, I610, I611, I612, I613, I614, I615, I616, I618, I619, I6300, I63011, I63012, I63013, I63019, I6302, I63031, I63032, I63039, I6309, I6310, I63111, I63112, I63119, I6312, I63131, I63132, I63139, I6319, I6320, I63211, I63212, I63213, I63219, I6322, I63231, I63232, I63233, I63239, I6329, I6330, I63311, I63312, I63313, I63319, I63321, I63322, I63323, I63329, I63331, I63332, I63333, I63339, I63341, I63342, I63343, I63349, I6339, I6340, I63411, I63412, I63413, I63419, I63421, I63422, I63423, I63429, I63431, I63432, I63433, I63439, I63441, I63442, I63443, I63449, I6349, I6350, I63511, I63512, I63513, I63519, I63521, I63522, I63523, I63529, I63531, I63532, I63533, I63539, I63541, I63542, I63543, I63549, I6359, I636, I638, I639, I6601, I6602, I6603, I6609, I6611, I6612, I6613, I6619, I6621, I6622, I6623, I6629, I663, I668, I669, I67841, I67848, I6789, I97810, I97811, I97820, I97821

Tobacco Use	3051, 64900, 64901, 64902, 64903, 64904, 98984, F17200, F17201, F17203, F17208, F17209, F17210, F17211, F17213, F17218, F17219, F17220, F17221, F17223, F17228, F17229, F17290, F17291, F17293, F17298, F17299, O99330, O99331, O99332, O99333, O99334, O99335, T65211A, T65212A, T65213A, T65214A, T65221A, T65222A, T65223A, T65224A, T65291A, T65292A, T65293A, T65294A, Z720
Traumatic Brain Injury and Nonpsychotic Mental Disorders due to Brain Damage	310, 3100, 3101, 3102, 3108, 31081, 31089, 907, 9070, 9071, F070, F0781, F0789, F482, S04011S, S04012S, S04019S, S0402XS, S04031S, S04032S, S04039S, S04041S, S04042S, S04049S, S0410XS, S0411XS, S0412XS, S0420XS, S0421XS, S0422XS, S0430XS, S0431XS, S0432XS, S0440XS, S0441XS, S0442XS, S0450XS, S0451XS, S0452XS, S0460XS, S0461XS, S0462XS, S0470XS, S0471XS, S0472XS, S04811S, S04812S, S04819S, S04891S, S04892S, S04899S, S049XXS, S060X0S, S060X1S, S060X2S, S060X3S, S060X4S, S060X5S, S060X6S, S060X7S, S060X8S, S060X9S, S061X0S, S061X1S, S061X2S, S061X3S, S061X4S, S061X5S, S061X6S, S061X7S, S061X8S, S061X9S, S062X0S, S062X1S, S062X2S, S062X3S, S062X4S, S062X5S, S062X6S, S062X7S, S062X8S, S062X9S, S06300S, S06301S, S06302S, S06303S, S06304S, S06305S, S06306S, S06307S, S06308S, S06309S, S06310S, S06311S, S06312S, S06313S, S06314S, S06315S, S06316S, S06317S, S06318S, S06319S, S06320S, S06321S, S06322S, S06323S, S06324S, S06325S, S06326S, S06327S, S06328S, S06329S, S06330S, S06331S, S06332S, S06333S, S06334S, S06335S, S06336S, S06337S, S06338S, S06339S, S06340S, S06341S, S06342S, S06343S, S06344S, S06345S, S06346S, S06347S, S06348S, S06349S, S06350S, S06351S, S06352S, S06353S, S06354S, S06355S, S06356S, S06357S, S06358S, S06359S, S06360S, S06361S, S06362S, S06363S, S06364S, S06365S, S06366S, S06367S, S06368S, S06369S, S06370S, S06371S, S06372S, S06373S, S06374S, S06375S, S06376S, S06377S, S06378S, S06379S, S06380S, S06381S, S06382S, S06383S, S06384S, S06385S, S06386S, S06387S, S06388S, S06389S, S064X0S, S064X1S, S064X2S, S064X3S, S064X4S, S064X5S, S064X6S, S064X7S, S064X8S, S064X9S, S065X0S, S065X1S, S065X2S, S065X3S, S065X4S, S065X5S, S065X6S, S065X7S, S065X8S, S065X9S, S066X0S, S066X1S, S066X2S, S066X3S, S066X4S, S066X5S, S066X6S, S066X7S, S066X8S, S066X9S, S06810S, S06811S, S06812S, S06813S, S06814S, S06815S, S06816S, S06817S, S06818S, S06819S, S06820S, S06821S, S06822S, S06823S, S06824S, S06825S, S06826S, S06827S, S06828S, S06829S, S06890S, S06891S, S06892S, S06893S, S06894S, S06895S, S06896S, S06897S, S06898S, S06899S, S069X0S, S069X1S, S069X2S, S069X3S, S069X4S, S069X5S, S069X6S, S069X7S, S069X8S, S069X9S
Viral Hepatitis (General)	0700, 0701, 0702, 07020, 07021, 07022, 07023, 0703, 07030, 07031, 07032, 07033, 0704, 07041, 07042, 07043, 07049, 0705, 07051, 07052, 07053, 07054, 07059, 0706, 0707, 07070, 07071, 0709, B150, B159, B160, B161, B162, B169, B170, B1710, B1711, B172, B178, B179, B180, B181, B182, B188, B189, B190, B1910, B1911, B1920, B1921, B199, V026, V0260, V0261, V0262, V0269, Z2250, Z2251, Z2252, Z2259
Sleep disorders	F13282, 78058, Z73811, F13288, G4722, 78051, F13982, 32726, P283, G4733, G47411, 30744, 34701, 34710, 32737, G47429, F515, G4711, 32720, 30746, 4672, 32734, 32743, 32730, 34711, F514, 32712, F19282, F14182, G4752, 78055, 29285, G4709, G4734, 30749, G4720, G4737, 32727, G47421, F11182, 32729, F14282, F5103, 32736, G4723, G4753, F13182, 30745, 30741, G4712, Z73819, G4730, G4701, F5112, F5105, G4726, 29182, G4739, Z73812, 32724, 30743, 78054, G4762, G478, 30742, F10282, F15182, F19982, V694, 32701, G4763, 32721, F513, 78056, 78057, 32700, F5104, 32725, G4724, 32722, 32732, 32739, G4769, 32740, 32731, 32711, 32709, 32723, Z72820, 78052, A8183, 32702, 32735, 32742, F10982, F5101, 30740, F11282, F519, Z73810, G4725, F14982, G4727, F11982, 30747, G4729, 78053, F5109, F518, 32733, G4721, G4700, F15982, 32759, G47419, G4731, F10182, 78059, G4736, F15282, 3278, F5102, 30748, 34700, G479, Z72821

Periodontitis	K05319, 52342, K056, 52341, K08121, K08124, K05212, K05223, K08122, K0530, K054, 5239, 52331, K044, K08421, K05222, K05313, K05312, K05323, K08429, K05321, K05329, 52330, K05211, K05221, 5238, K08424, K05311, K08129, 5226, K055, K05229, K08422, K05213, 5235, K05219, K0520, 52340, 52333, K045, K05322, 5224, 52332, K08123, K08423
Menopause	E28319, 6274, 6270, E28310, N924, 25631
