# Supplementary material for: High-throughput target trial emulation for Alzheimer’s disease drug repurposing with real-world data
Source: Nat Commun. 2023 Dec 11;14:8180. doi: 10.1038/s41467-023-43929-1 (PMC10713627; doi:10.1038/s41467-023-43929-1)
Supplement: Supplementary file 3 — Reporting Summary [file 41467_2023_43929_MOESM3_ESM.pdf]

nature portfolio

Corresponding author(s): Fei Wang

Last updated by author(s): Oct 30, 2023

Reporting Summary

Nature Portfolio wishes to improve the reproducibility of the work that we publish. This form provides structure for consistency and transparency in reporting. For further information on Nature Portfolio policies, see our Editorial Policies and the Editorial Policy Checklist.

Statistics

For all statistical analyses, confirm that the following items are present in the figure legend, table legend, main text, or Methods section.

n/a	Confirmed	
☐	☒	The exact sample size (*n*) for each experimental group/condition, given as a discrete number and unit of measurement
☐	☒	A statement on whether measurements were taken from distinct samples or whether the same sample was measured repeatedly
☐	☒	The statistical test(s) used AND whether they are one- or two-sided<br>*Only common tests should be described solely by name; describe more complex techniques in the Methods section.*
☐	☒	A description of all covariates tested
☐	☒	A description of any assumptions or corrections, such as tests of normality and adjustment for multiple comparisons
☐	☒	A full description of the statistical parameters including central tendency (e.g. means) or other basic estimates (e.g. regression coefficient) AND variation (e.g. standard deviation) or associated estimates of uncertainty (e.g. confidence intervals)
☐	☒	For null hypothesis testing, the test statistic (e.g. *F*, *t*, *r*) with confidence intervals, effect sizes, degrees of freedom and *P* value noted<br>*Give P values as exact values whenever suitable.*
☒	☐	For Bayesian analysis, information on the choice of priors and Markov chain Monte Carlo settings
☒	☐	For hierarchical and complex designs, identification of the appropriate level for tests and full reporting of outcomes
☒	☐	Estimates of effect sizes (e.g. Cohen's *d*, Pearson's *r*), indicating how they were calculated

Our web collection on statistics for biologists contains articles on many of the points above.

Software and code

Policy information about availability of computer code

Data collection	No particular software was used for collecting the data.
Data analysis	We implemented our high-throughput target trial emulation system for drug repurposing using Python 3.9 and Pytorch 1.8 and trained deep learning models by Adam optimizer60 on a Linux server with two GeForce RTX 2080 Ti GPUs and 16 CPU cores. We used the Python package lifelines-0.26 for survival analysis,61 scikit-learn-0.23 for machine learning models including regularized logistic regression,62 and lightgbm-3.2 for the gradient boosting machine.17 Python package gcastle 1.0.3 for stable PC algorithm. We followed Liu et al. for their LSTM-PS implementations.7 We randomly partitioned each emulated trial into complementary training and testing data sets with a ratio of 80:20, and 10-fold cross-validations were conducted on the training set. Please refer to our python package for more details. For reproducibility, we open-sourced our Python code package at https://github.com/calvin-zcx/RWD4Drug with DOI: 10.5281/zenodo.10070359.

For manuscripts utilizing custom algorithms or software that are central to the research but not yet described in published literature, software must be made available to editors and reviewers. We strongly encourage code deposition in a community repository (e.g. GitHub). See the Nature Portfolio guidelines for submitting code & software for further information.

Data

Policy information about availability of data

All manuscripts must include a data availability statement. This statement should provide the following information, where applicable:

- Accession codes, unique identifiers, or web links for publicly available datasets
- A description of any restrictions on data availability
- For clinical datasets or third party data, please ensure that the statement adheres to our policy

The OneFlorida data can be requested through https://onefloridaconsortium.org/front-door/. Since the OneFlorida data is a HIPAA-limited data set, a data use agreement needs to be established with the OneFlorida network. The MarketScan dataset is available from IBM at https://www.ibm.com/products/marketscan-research-databases. The relevant raw data for each figure and table are provided in the Source Data file.

Research involving human participants, their data, or biological material

Policy information about studies with human participants or human data. See also policy information about sex, gender (identity/presentation), and sexual orientation and race, ethnicity and racism.

Reporting on sex and gender	This is a retrospective secondary analysis of de-identified patient records from two RWDs including an electronic health record (EHR) cohort and a general claims cohort. Summary statistics on self-reported gender distributions within different cohorts were reported in Supplement Table S1.
Reporting on race, ethnicity, or other socially relevant groupings	We didn't report these information because the MarketScan claim data didn't have information on race, ethnicity or other socially relevant groupings.
Population characteristics	The OneFlorida database contains robust patient-level electronic health record data for nearly 15 million (14,883,388) patients majorly from Florida and selected cities in Georgia and Alabama from January 2012 to April 2020, and the IBM MarketScan database (formerly known as Truven) contains administrative claim records from January 2009 to June 2020 for over 164 million (164,148,434) enrollees across the US, serving as a nationally representative database of the US population (See Supplementary Tables 1 for the population characteristics of two databases). We included all patients with at least one mild cognitive impairment (MCI) diagnosis between January 2012 and April 2020 recorded in the OneFlorida database (January 2009 to Jun 2020 in the MarketScan data). Patients required with age ≥ 50 years old at MCI diagnosis, no history of AD or AD-related dementia diagnoses within five years before the index date, the first MCI diagnosis date should be before the index date, and the baseline period captured in the database should ≥ one year without an upper limit. We defined the index date as the date of initiation of the trial drug, and at baseline, all of the above criteria should have been met.
Recruitment	This is a retrospective secondary analysis of EHR data and no patient recruitment activities are involved.
Ethics oversight	This study was approved by the Institutional Review Board of Weill Cornell Medicine with protocol number 21-07023759. The use of OneFlorida data for this study is approved under the University of Florida IRB number IRB202001888. Access to the MarketScan data analyzed in this manuscript is provided by the University of Kentucky.

Note that full information on the approval of the study protocol must also be provided in the manuscript.

Field-specific reporting

Please select the one below that is the best fit for your research. If you are not sure, read the appropriate sections before making your selection.

- [x] Life sciences
- [ ] Behavioural & social sciences
- [ ] Ecological, evolutionary & environmental sciences

For a reference copy of the document with all sections, see nature.com/documents/nr-reporting-summary-flat.pdf

Life sciences study design

All studies must disclose on these points even when the disclosure is negative.

Sample size	The OneFlorida database contains robust patient-level electronic health record data for nearly 15 million (14,883,388) patients majorly from Florida and selected cities in Georgia and Alabama from January 2012 to April 2020, and the IBM MarketScan database (formerly known as Truven) contains administrative claim records from January 2009 to June 2020 for over 164 million (164,148,434) enrollees across the US, serving as a nationally representative database of the US population (See Supplementary Tables S1 for the population characteristics of two databases). We started from all the recorded patients as mentioned above, and after the cohorts selection (eligibility criteria below, or in the method section), the OneFlorida database (see Data Section) as our discovery set, we included 73,927 patients with MCI diagnosis from 2012 to 2020 (Fig. 1a). Regarding MarketScan, we identified a total of 424,961 MCI patients from 2009 to 2020 and among which, there were 2,489 unique drug ingredients. Our analyses covered both large-scale and longitudinal EHR and administrative claims.
Data exclusions	We included patients with at least one mild cognitive impairment (MCI) diagnosis between January 2012 and April 2020 in the OneFlorida database (January 2009 to Jun 2020 in the MarketScan data). Patients required with age ≥ 50 years old at MCI diagnosis, no history of AD or AD-related dementia diagnoses within five years before the index date, the first MCI diagnosis date should be before the index date, and the baseline period captured in the database should ≥ one year without an upper limit. We defined the index date as the date of initiation of the trial drug, and at baseline, all of the above criteria should have been met.

Replication	The main analysis was conducted on the OneFlorida+ cohort and a replication analysis was done on the MarketScan cohort. We tried to use exactly the same analyses pipeline in the replication analysis as in the primary analysis, including the same eligibility criteria, clinical phenotyping algorithms, adjustment analyses, sensitivity analyses etc as in our primary analyses. We grouped drug prescriptions coded as National Drug Code (NDC) or RXNORM codes into their major active ingredients coded in RXNORM defined in Unified Medical Language System52 for the OneFlorida and into the Medi-Span Generic Product Identifier (GPI)53 by their first 8 digits for the MarketScan data. All attempts at replication were successful.
Randomization	This is a retrospective analysis based on clustering and no randomization procedure was involved as no treatment effect was assessed. We assumed that the treated group and comparison group were exchangeable at baseline conditional on baseline covariates, including age, gender, baseline comorbidities, medications, and time from the MCI diagnosis date to the drug initiation date. The baseline comorbidities consisted of selected comorbidities from Chronic Conditions Data Warehouse53 and established risk factors for AD selected by experts, resulting in 64 covariates (Supplementary Table 3); each defined by a set of selected ICD-9/10 codes. We grouped drug prescriptions coded as National Drug Code (NDC) or RXNORM codes into their major active ingredients coded in RXNORM defined in Unified Medical Language System54 for the OneFlorida case and into the Medi-Span Generic Product Identifier (GPI)55 by their first 8 digits for the MarketScan data. We used the top 200 prevalent prescribed drug ingredients for the covariates for the medication history. The age and the time from the MCI diagnosis date to the drug initiation date were encoded as continuous variables, and the gender, comorbidities, and medication uses were encoded as binary variables. In total, there were 267 covariates to adjust for. In addition to the 267 baseline covariates, we also considered the sequences of each of the comorbidities and medications variables over time for the deep long short-term memory network with attention mechanisms-based PS calculation.<br><br>On the other hand, following the arguments that bad controls should not be adjusted for,35 we also built the baseline covariates by considering hypothetical causal diagrams built by both existing knowledge and data-driven causal discovery algorithms. Specifically, based on the best available knowledge, we selected a subset of baseline variables that are risk factors for or associated with AD, including age (the single most significant factor), gender, hypertension, hyperlipidemia, obesity, diabetes, heart failure, stroke, ischemic heart disease, traumatic brain injury due to brain damage, anxiety disorders, sleep disorders, alcohol use disorders, menopause, and periodontitis.3,36 Next, we applied the constraint-based causal structure learning algorithm stable PC-algorithm 37 to each emulated trial to learn its likely underlying directed acyclic graph. For each emulated trial, we excluded detected colliders (including M-colliders) and mediators and assumed that the remaining covariates are more likely to be confounders of the treatment assignment and the AD onset to adjust for. We used corrected significance level 2.9×10^(-4) and Fisher-z's test for the stable PC-algorithm.
Blinding	This is a retrospective analysis and there is no blinding to the treatment status. This is real-world data analyses and patients were aware of their treatment or exposures.

Reporting for specific materials, systems and methods

We require information from authors about some types of materials, experimental systems and methods used in many studies. Here, indicate whether each material, system or method listed is relevant to your study. If you are not sure if a list item applies to your research, read the appropriate section before selecting a response.

Materials & experimental systems

n/a	Involved in the study
☒	☐ Antibodies
☒	☐ Eukaryotic cell lines
☒	☐ Palaeontology and archaeology
☒	☐ Animals and other organisms
☒	☐ Clinical data
☒	☐ Dual use research of concern
☒	☐ Plants

Methods

n/a	Involved in the study
☒	☐ ChIP-seq
☒	☐ Flow cytometry
☒	☐ MRI-based neuroimaging
